# Cathepsin D promotes acute myeloid leukemia progression through stabilization of the anti-apoptotic proteins

**DOI:** 10.1038/s41419-025-07949-7

**Published:** 2025-08-12

**Authors:** Huimin Jiang, Yongjian Wang, Churan Wang, Lu Yang, Shujuan Wang, Feng Wang, Situ Xue, Zhuan Zhang, Haigen Fu, Ting Dong, Jian Yuan, Zhuorong Li, Ke Li

**Affiliations:** 1 State Key Laboratory of Bioactive Substance and Function of Natural Medicines, NHC Key Laboratory of Biotechnology of Antibiotics, Institute of Medicinal Biotechnology, Chinese Academy of Medical Sciences and Peking Union Medical College, Beijing, China; 2 Shanghai Institute of Hematology, Blood and Marrow Transplantation Center, Collaborative Innovation Center of Hematology, Department of Hematology, Ruijin Hospital, Shanghai Jiao Tong University School of Medicine, Shanghai, China; 3 Department of Hematology, The First Affiliated Hospital of Zhengzhou University, Zhengzhou, China; 4 State Key Laboratory of Cardiology and Research Center for Translational Medicine, Shanghai East Hospital, Tongji University School of Medicine, Shanghai, China

**Keywords:** Acute myeloid leukaemia, Apoptosis, Ubiquitylation, Virtual screening

## Abstract

Cathepsin D (CTSD) is a lysosomal aspartic protease that plays vital roles in regulating the properties of solid tumors, including proliferation, apoptosis, migration, metastasis, and angiogenesis. However, the function of CTSD in haematological malignancies remains largely elusive. Here we show that CTSD is highly expressed in acute myeloid leukemia (AML) and that high CTSD expression is associated with unfavourable prognosis. Knockdown of *CTSD* in AML cells inhibits cell proliferation and anti-apoptotic activity. Mechanistically, CTSD decreased the expression of the E3 ubiquitin ligase TRIM21, which mediates the ubiquitination and degradation of anti-apoptotic proteins BCL2, BCL-XL, and MCL1. Inhibition of CTSD expression via genetics or the small-molecule inhibitor **N-8** decreases the protein levels of BCL2, BCL-XL, and MCL1 through accelerating their degradation. **N-8** shows significant efficacy in eradicating AML in both venetoclax-sensitive and -resistant models. Collectively, our study reveals the role of CTSD in leukemia progression and highlights targeting CTSD as a potential therapeutic strategy in AML.

## Introduction

Acute myeloid leukemia (AML), one of the most common types of adult leukemia, is characterised by the abnormal growth and differentiation of haematopoietic stem cells. This abnormal behaviour results in the excessive accumulation of immature myeloid precursors in the bone marrow and peripheral blood [1]. While AML is primarily classified based on genetic abnormalities in the latest World Health Organization (WHO) and European Leukemia Network (ELN) guidelines [2, 3], the French-American-British (FAB) system still retains clinical utility, particularly for rapid morphological assessment and historical comparisons. According to the FAB classification, AML is divided into eight subtypes based on the morphology and differentiation level of leukemia cells, with subtypes M0 to M7 reflecting a progressive increase in differentiation [4]. Acute monocytic leukemia, which accounts for 10% of AML cases, is associated with poor survival and shorter duration of remission [5]. The current standard treatment for AML is the 7 + 3 regimen, which consists of cytarabine (Ara-C) and daunorubicin. However, this approach has limited tolerability and only results in modest improvements in patient survival [6]. The development of targeted therapies has provided new treatment options for patients with AML. ABT-199 (venetoclax), an FDA-approved BCL2 inhibitor, has shown efficacy and good tolerability [7–9]. Despite the significantly improved response rates, venetoclax is not curative, and resistance typically develops over time [8]. The factors contributing to resistance include *BCL2* mutations [10], elevated levels of MCL1 and BCL-XL [11], and *TP53* aberrations [12]. Therefore, there is an urgent need to identify novel and effective therapeutic targets.

Cathepsin D (CTSD), a member of the aspartic protease family, is an acidic enzyme that is predominantly active in cellular environments with low pH, such as lysosomes [13]. It plays a critical role in protein degradation and processing, influencing various physiological and pathological processes [14]. In response to external stimuli or environmental changes, CTSD can translocate to the cytoplasm or other subcellular compartments [13]. This translocation allows CTSD to regulate key cellular processes, including apoptosis, cell cycle progression, and autophagy [15, 16]. Recent findings have indicated that inhibition of CTSD enhances anticancer drug-induced apoptosis through the RNF183-mediated destabilization of BCL-XL in cancer cells [17]. Furthermore, CTSD is highly expressed in breast, gastric, and ovarian cancers, where it is closely linked to cancer proliferation, angiogenesis, and metastasis [18–21]. In mammary epithelial cells, CTSD deficiency disrupts mTORC1 signalling and delays breast cancer progression [22]. Notably, immunomodulatory antibodies targeting CTSD are a promising immunotherapy strategy for patients with triple-negative breast cancer (TNBC) [23]. Despite these findings, the exact role of CTSD in the progression of AML remains unclear.

Anti-apoptotic BCL2 family members, including BCL2, BCL-XL, and MCL1, suppress apoptosis and are upregulated in cancer cells, including leukemia cells, where their expression is associated with poor prognosis [24–26]. Ubiquitination, mediated by E3 ligases, is pivotal for regulating BCL2 members’ protein stability and function [27]. For example, GSK3β phosphorylates MCL1, targeting it for FBXW7-mediated ubiquitination and degradation [28, 29]. Mcl-1 ubiquitin ligase E3 (MULE) also binds MCL1 to decrease its stability [30]. Similarly, the pro-apoptotic tumor suppressor ATRS recruits the E3 ubiquitin ligase XIAP to ubiquitinate BCL2 at K17, destabilizing it [31]. Interestingly, the inhibition of both BCL2 and XIAP has been shown to benefit AML treatment [32]. The ubiquitination of BCL-XL is mediated by E3 ligases such as RNF183 and PARK2, thereby increasing apoptosis [33, 34]. As evidenced by the critical roles of the BCL2 family in apoptosis, using ubiquitination and degradation to target anti-apoptotic proteins represents an effective anticancer strategy [35]. However, there is no known common E3 ligase that degrades BCL2, BCL-XL, and MCL1. Identifying a shared degradation mechanism and developing targeted molecules for anti-apoptosis protein degradation are crucial for cancer therapy.

In this study, we show that CTSD is highly expressed in AML, especially in the monocytic AML, which promotes AML progression by maintaining the stability of the anti-apoptotic proteins BCL2, BCL-XL, and MCL1. Mechanistically, we show that CTSD suppresses the ubiquitination and degradation of BCL2, BCL-XL, and MCL1 mediated by the E3 ubiquitin ligase TRIM21, ultimately promoting the proliferation and survival of AML cells. In addition, we screen for small-molecule inhibitors of CTSD and evaluate the therapeutic potential of targeting CTSD in AML. Collectively, this study reveals that CTSD is a regulator of AML and a potential therapeutic target in AML.

## Methods

### Cell lines

Human leukemia cell lines HL60, Kasumi, U937, and MV4-11 were purchased from National Collection of Authenticated Cell Cultures (Shanghai, China). Human leukemia KG1 cells was purchased from ATCC (Manassas, Maryland, USA). OCI-AML3 and MOLM-13 were purchased from DSMZ (Braunschweig, Germany). Human leukemia NB4 cells was purchased from Bioleaf Biotech Co., Ltd (Shanghai, China). Lenti-X 293 T cell line was purchased from Takara Bio (Otsu, Shiga, Japan). All cell lines were recently authenticated by short tandem repeat (STR) profiling and tested negative for mycoplasma detection. KG1, Kasumi, HL60, NB4, OCI-AML3, U937, MOLM-13, and MV4-11 cells were cultured in RPMI 1640 (#03.4007 C, EallBio, Beijing, China) supplemented with 20% fetal bovine serum (FBS) (#03.U16001DC, EallBio, Beijing, China) and 1% penicillin-streptomycin antibiotics (#FG101-01, TransGene Biotech, Beijing, China). Lenti-X 293 T cells were maintained in Dulbecco’s modified Eagle’s medium (DMEM) (#03.1002 C, EallBio, Beijing, China) with 10% FBS and 1% penicillin-streptomycin. All cells were grown at 37 °C in a humidified atmosphere with 5% CO_2_.

### Mice

NOD-SCID IL2Rg-null (NSG) mice (6-8 weeks old, female; GemPharmatech, Nanjing, China) were used to establish the U937 xenograft model. C57BL/6 J mice (6-8 weeks old, female; HFK Bioscience, Beijing, China) were used for the *MLL-AF9* mouse model. Female mice were allowed to be used for leukemia studies. For animal studies, the mice were earmarked before grouping and then were randomly separated into groups by an independent person. However, no particular method of randomization was used. Sample size was predetermined empirically according to previous experience using the same strains and treatments. Generally, we used *n* ≥ 5 mice per condition. We ensured that the experimental groups were balanced in terms of animal age and weight. Euthanasia was performed using an approved method, to minimize animal distress. All animal procedures were conducted in accordance with the guidelines of the Institutional Committee for the Ethics of Animal Care and Treatment in Biomedical Research of Chinese Academy of Medical Sciences (CAMS) and Peking Union Medical College (PUMC). The animal study was conducted in accordance with the Animal Research: Reporting of In Vivo Experiments (ARRIVE) guidelines.

### Primary patient sample

Human primary AML patient sample was obtained from the Institutional Review Board of the First Affiliated Hospital of Zhengzhou University. Informed consent was obtained in accordance with the Declaration of Helsinki. Primary AML cells were freshly isolated from the bone morrow of a relapsed AML-M5b patient (Supplementary table 1) using human lymphocyte separation medium (#P8610, Solarbio, Beijing, China). The collected cells were thoroughly washed with PBS and subsequently cultured in RPMI 1640 medium supplemented with 20% FBS and 2% penicillin-streptomycin.

### Generation of Stable cell lines

The *CTSD* and *TRIM21* knockdown cell lines were generated by lentiviral transduction with *CTSD*-shRNA and *TRIM21*-shRNA plasmids. Three shRNA sequences were designed using the Broad Institute Portal (portals.broadinstitute.org) and shown in Supplementary Table 2. After 48 h of *CTSD*-shRNA viral infection, the cells were sorted by FACS Aria III and then cultured for three weeks. Knockdown efficiency was verified by western blot analysis. Among the three *CTSD* knockdown cell lines established, *CTSD-*shRNA2 was primarily used for subsequent experiments. Similarly, cells infected with *TRIM21*-shRNAs were selected using puromycin (#A1113802, Gibco, Massachusetts, USA), and shRNA3 was chosen for its superior knockdown efficiency in parallel studies.

### Selection of venetoclax-resistant cell line

To generate venetoclax-resistant MOLM-13 (MOLM-13-VEN-R) and MV4-11 (MV4-11-VEN-R) cell lines, the cells were cultured in RPMI 1640 medium supplemented with 0.1 μM venetoclax. The drug concentration was gradually increased to 2.5 μM over six months. At the end of this period, the cells exhibited stable growth in the presence of venetoclax, confirming the achievement of resistance. The MOLM-13-VEN-R and MV4-11-VEN-R cell lines were subsequently used to measure **N-8** IC_50_ and pro-apoptotic effects.

### Murine Xenograft Model of Leukemia

To evaluate the effect of *CTSD* knockdown on AML progression in vivo, NSG mice were intravenously injected with 1 × 10^6^ U937 *CON*-shRNA and *CTSD-*shRNA cells per mouse. Three weeks later, leukemia burden and apoptosis of leukemia cells in PB, BM, and spleen were analyzed by flow cytometry. For the U937 xenograft model, NSG mice were injected via the tail vein with 1 × 10^6^ U937 cells per mouse. Five days after transplantation, the mice were randomly separated into three groups (*n* = 5 per group) and treated with either the vehicle, **N-8**, and Ara-C. **N-8** was administrated orally (PO) at a dose of 20 mg/kg once daily (QD) for three weeks, while the positive control drug, Ara-C, was administered intraperitoneally at 10 mg/kg every other day (QOD). For the *MLL-AF9* leukemia model, bone marrow cells were extracted from 6–8 weeks C57BL/6 J mice. Lineage negative cells (Lin^-^) were enriched using a hematopoietic stem/progenitor cell enrichment kit (#19856A, StemCell, Vancouver, Canada) and infected twice with MSCV-*MLL-AF9*-IRES-GFP retroviruses. Infected cells (2 × 10^5^ cells/mouse) were then transplanted into irradiated (9.0 Gy) C57BL/6 mice via tail vein injection. For the second transplantation, 1 × 10^5^ spleen cells from primary leukemia mice were intravenously injected into irradiated (4.5 Gy) C57BL/6 J mice. Five days later, the mice were randomly divided into three groups (*n* = 5 per group) and treated with either the vehicle, **N-8** (30 mg/kg, PO, QD), and venetoclax (100 mg/kg, PO, QD). Three weeks post-transplantation, mice were scarified, and apoptosis of cells in the PB, BM, and spleen was analyzed by flow cytometry. Mice were considered to have successfully established the leukemia mouse models if the proportion of leukemia cells in their peripheral blood exceeded 1% five days after the injection of leukemia cells. Animals in which leukemia did not develop were excluded from the study.

### Cell proliferation assay

For the cell growth assay, cell lines were seeded at a density of 5000 cells per well. Starting on day 2, 10 μL of CCK-8 solution (#C0005, TargetMol, Shanghai, China) was added daily, followed by 2 h incubation at 37 °C and absorbance measurement at 450 nm. For the CCK-8 assay, cells were seeded at a density of 8000–10,000 cells per well, treated with compound **N-8** at the indicated concentrations and cultured for 48 h. All experiments were performed in six replicates.

### Coimmunoprecipitation and Western blotting

For co-immunoprecipitation (Co-IP), cells were harvested and lysed in Co-IP lysis buffer. To analyze ubiquitylation levels, cells were pretreated with MG132 (10 μM) for 4 h, and MG132 was also added to the lysis buffer. Cells were lysed on ice and the protein lysates were then incubated overnight at 4 °C with the indicated magnetic/gel bead-labeled antibodies or Protein A/G (#sc-2003, Santa Cruz Biotechnology, California, USA) plus antibodies, under constant rotation. The immunoprecipitates were washed five times with Co-IP wash buffer and denatured by boiling in 2 × loading buffer before subsequent western blot analysis. Another mothed for protein extraction from cells, BM, or spleen tissue is through RIPA lysis (#C1053, Applegen, Beijing, China). Protein concentration were determined using BCA Protein Assay Kit (#1511, Applegen, Beijing, China). Protein extracts were separated by SDS-PAGE, transferred onto PVDF membranes, and subjected to immunoblot analysis. Western blot images were captured using the Tanon 5200 chemiluminescent imaging system (Tanon, Shanghai, China). Immunoblot images have been cropped for presentation. Full and uncropped immunoblot images are provided in the Supplemental Material.

### Quantitative real-time PCR

Total RNA was extracted from cells using the RNA extraction Kit (#RN001, ES science, Shanghai, China), according to the manufacturer’s instruction. Reverse transcription of total cellular RNA was performed using the NovoScript Plus All-in-one 1st Strand cDNA Synthesis SuperMix (#E047, Novoprotein, Suzhou, China). PCR amplification was conducted in triplicate, with each reaction containing NovoStart SYBR qPCR SuperMix Plus (#E096-01A, Novoprotein, Suzhou, China), mixed primers, and template cDNA. The PCR was performed using a MyCycler thermal cycler (844-00553-2, qTOWER, Analytik Jena, German). The primer sequences are shown in Supplementary Table 3.

### Flow cytometry

For the analysis of AML progression in animal models, PB, BM, and spleen cells were processed following red blood lysis and stained with PE/Cyanine7 anti-human CD45 antibody (#368532, Biolegend, San Diego, USA) for 30 min at room temperature. For the apoptosis assay, 2 × 10^5^ cells were washed with PBS and incubated with Annexin V-APC (#AD11, Dojindo, kumamoto, Japan) for 30 min at room temperature. Before measuring the percentage of apoptotic cells, propidium iodide (PI) was added to the treated cells. Analysis was performed using a flow cytometer (A00-1-1102, Beckman Coulter, Brea, California, USA). Data was analyzed with FlowJo software (version 10.8.1).

### Molecular docking

The X-ray structure of the CTSD protein (PDB ID: 5UX4) was downloaded from the Protein Data Bank (https://www.rcsb.org). The protein was prepared according to standard protocols. Co-crystallized water molecules were removed, and potential issues in the protein structure were addressed using the “Clean Protein” and “Prepare Protein” tools in Discovery Studio, which included modeling missing loop regions, removing alternative conformations, adding hydrogen atoms, and generating the protonation state at pH 7.0. The active site was defined based on the original co-crystallized ligand, with the docking sphere radius set to 7 Å. The coordinates for the original ligand’s active site were defined as X = 43.218727 Å, Y = 7.777818 Å, Z = 17.708121 Å. These compounds underwent “full minimization”. Discovery Studio 3.0 was utilized to perform the LibDock method for docking-based virtual screening.

### Plasmids and transfection

CTSD-Myc (#HG12517-CM), BCL2-Flag (#HG10195-CF), BCL-XL-untagged (#HG10455-M), MCL1-untagged (#HG10240-M), TRIM21-Flag (#HG18010-CF), and UB-HA (#HG16831-NY) plasmids were purchased from Sino Biological Inc. (Beijing, China). pMD2.G (#12259) and psPAX2 (#12260) were purchased from Addgene (Cambridge, Massachusetts, USA). Additionally, the BCL-XL and MCL1 plasmids were subcloned into MIGR1 vectors using standard molecular cloning techniques, with HA, Flag, and Myc tags added. CTSD was subcloned into pMLV vector using standard molecular cloning protocol. Lenti-X 293 T cells were transfected with plasmids using PEI (#24765, Kyfora Bio, Beijing, China). U937, MV4-11, and MOLM-13 cells were transfected with *CTSD*-siRNA (#sc-29239, Santa Cruz Biotechnology, California, USA) using Lipofectamine RNAiMAX (#13778030, Thermo Fisher Scientific, Waltham, Massachusetts, USA) according to the manufacturer’s recommendations.

### Reagents and antibodies

CHX (#S7418) and MG132 (#S2619) were purchased from Selleck (Houston, Texas, USA). For the in vitro experiments, these agents were dissolved in DMSO (#D8372, Solarbio, Beijing, China) according to the solubility. For the in vivo experiments, Ara-C was dissolved in PBS (#LVN10022, Livning, Beijing, China). Venetoclax was dissolved in corn oil with 10% DMSO. **N-8** was dissolved in corn oil with 2% DMSO. Anti-CTSD (#ab75852) and anti-FBXW7 (#ab192328) antibodies were purchased from Abcam (Cambridge, UK). Anti-BCL2 (#A0208) antibody was purchased from Abclonal (Wuhan, China). Anti-BCL-XL (#10783-1-AP), anti-MCL1 (#16225-1-AP), anti-PELI1 (#12053-1-AP), anti-TRIM21 (#67136-1-Ig), and anti-β-Actin (#20536-1-AP) antibodies were purchased from Proteintech (Wuhan, China). Anti-ITCH (#D8Q6D) antibody was purchased from Cell Signaling Technology (Danvers, Massachusetts, USA). Anti-GAPDH (#TA-08) antibody was purchased from ZSGB-BIO (Beijing, China). Anti-Myc-tagged (#562), anti-DDDDK-tagged (#PM020), and anti-HA-tagged (#561) antibodies were purchased from MBL BIOTECH (Beijing, China).

### Statistics

Data are expressed as the mean ± standard error of the mean (S.E.M). Comparisons between two or more groups were performed using the unpaired Student’s *t-*test, one-way ANOVA, or two-way ANOVA. Correlation between groups was determined by Pearson’s correlation test. The survival rates were analyzed by the Kaplan–Meier method. The sample number (*n*) indicates the number of independent biological samples in each experiment. Sample numbers and experimental repeats are indicated in the figures and figure legends. Generally, all experiments were carried out with *n* ≥ 3 biological replicates. *P* < 0.05 was considered statistically significant. Analyses were performed using GraphPad Prism 10 software.

## Results

### CTSD is highly expressed in AML patients and correlates with poor prognosis

**Fig. 1:**
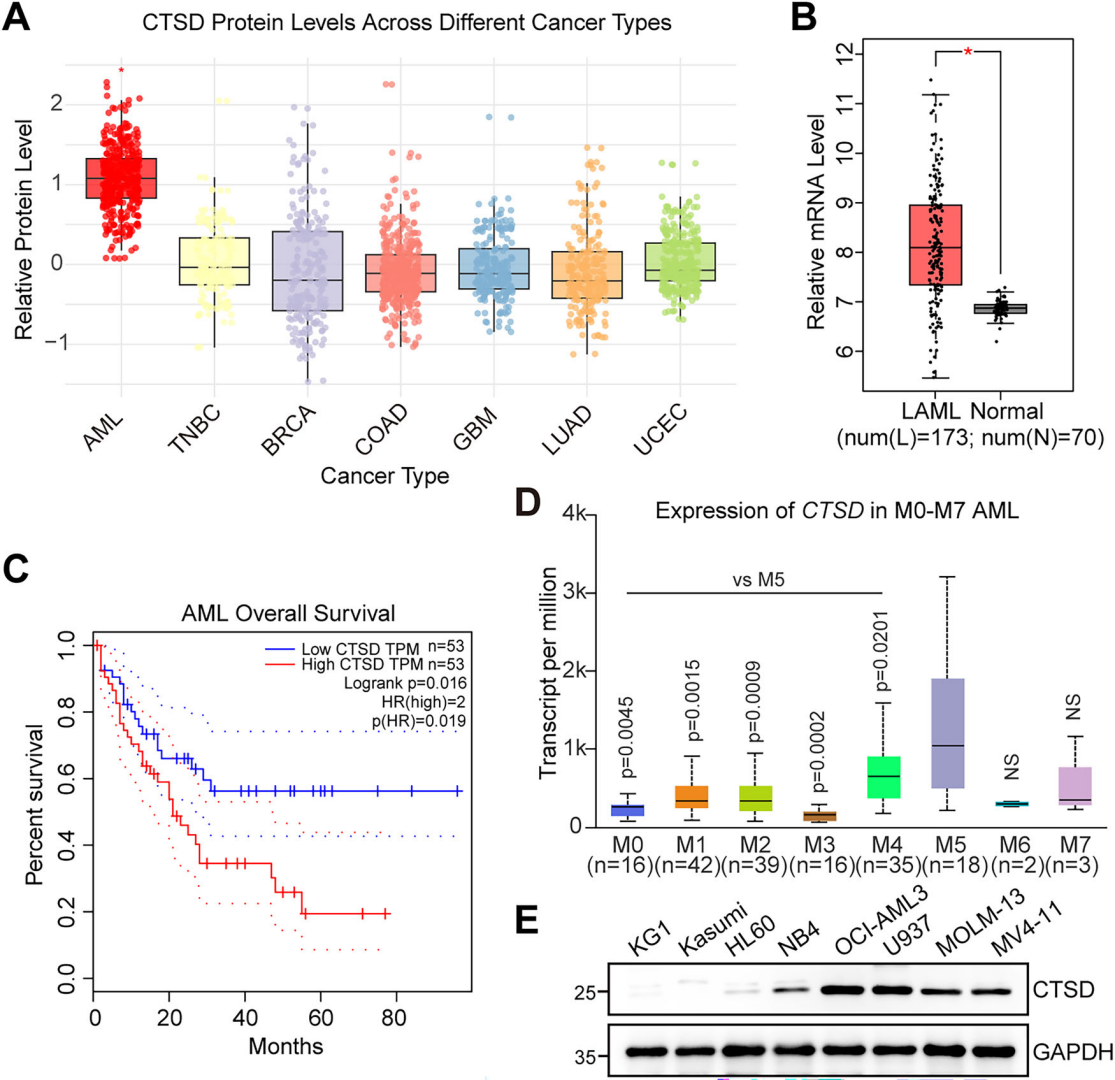
High CTSD expression is associated with poor prognosis in patients with AML. **A** The relative protein levels in different types of diverse cancer types were analyzed using the CPTAC database. AML, acute myeloid leukemia. TNBC, triple negative breast cancer. BRCA, breast invasive carcinoma. COAD, colon adenocarcinoma. GBM, glioblastoma multiforme. LUAD, lung adenocarcinoma. UCEC, uterine corpus endometrial carcinoma. **B** The relative mRNA levels of *CTSD* in patients with AML (LAML^a^, *n* = 173) and healthy donors (Normal, *n* = 70) were analyzed using the GEPIA database. ^a^LAML is the TCGA-standardized designation for Acute Myeloid Leukemia; the initial “L” denotes “Leukemia”, indicating its origin in the hematopoietic system. **C** Kaplan-Meier survival curves for patients with AML stratified by *CTSD* expression from the GEPIA database. **D** The mRNA levels of *CTSD* in different subtypes of AML were analyzed using the UALCAN database. **E** The protein levels of CTSD in eight AML cell lines were detected by western blotting.

To investigate the role of CTSD in cancer, we first examined the protein expression of CTSD in various cancer types using the Clinical Proteomic Tumor Analysis Consortium (CPTAC) database. CTSD expression was significantly higher in AML compared to other cancers, including TNBC, colon adenocarcinoma (COAD), and others (Fig. 1A). We then compared *CTSD* expression in AML patients and healthy donors using the Gene Expression Profiling Interactive Analysis (GEPIA) database (http://gepia.cancer-pku.cn) and found that *CTSD* levels were significantly elevated in AML patients (Fig. 1B). In addition, AML patients stratified into low- and high-*CTSD* groups by median *CTSD* expression demonstrated significantly shorter overall survival in the high-*CTSD* expression group compared to the low-*CTSD* expression group (Fig. 1C). Furthermore, higher *CTSD* expression was found in AML patients whose malignant cells have monocytic phenotypes (AML-M4 and -M5 subtypes) than those with the M0, M1, M2, and M3 subtypes of AML (Fig. 1D). We also detected the protein levels of CTSD in eight AML cell lines and found that CTSD was highly expressed in cell lines with monocytic differentiation, such as OCI-AML3, U937, MV4-11, and MOLM-13 cell lines (Fig. 1E). The genetic mutation information of the cell lines used were provided in the Supplementary Figure 1A. We also analyzed the correlation between *CTSD* expression and gene mutation landscape by using the University of Alabama at Birmingham Cancer data analysis (UALCAN) database (https://ualcan.path.uab.edu/index.html). No significant association was observed between *CTSD* expression and common mutations such as *FLT3*, *PML-RARA*, or *RAS* (Supplementary Fig. 1B). Collectively, these findings suggest that CTSD expression is associated with AML differentiation and may play a critical role in monocytic AML, including M4 and M5 subtypes.

### CTSD promotes the proliferation and survival of AML cells and the progression of AML

**Fig. 2:**
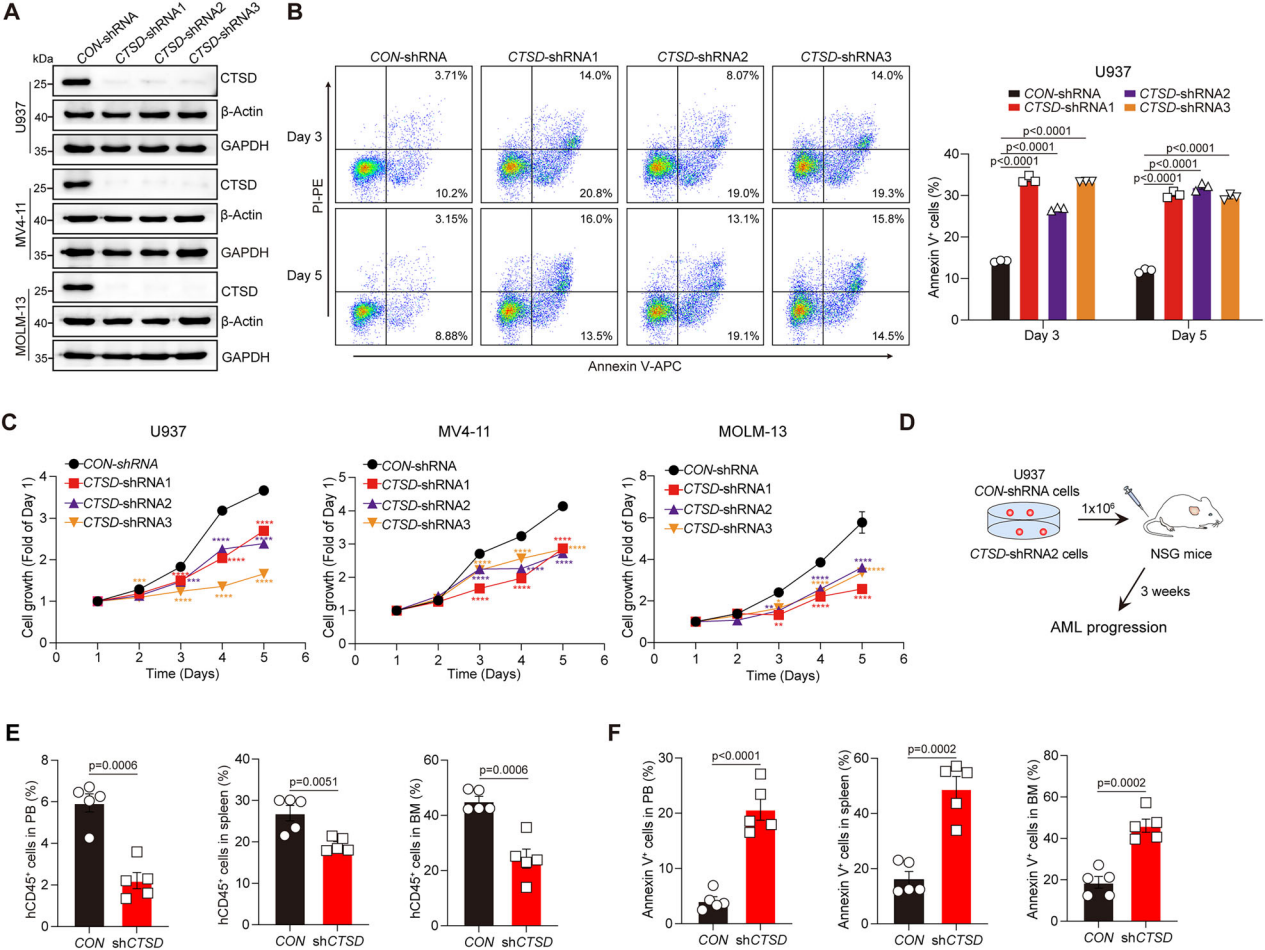
CTSD enhances the proliferation and survival of AML cells to promote AML progression. **A** The protein levels of CTSD in U937, MV4-11, and MOLM-13 cells with *CON*- or *CTSD*-shRNA were detected using western blotting. **B** Representative two-dimensional FACS plots (left) and flow cytometric analysis of apoptotic cell proportions in U937 cells with or without *CTSD* knockdown on days 3 and 5 (right). Annexin V^+^ cells were quantified using FlowJo software. Data are presented as the mean ± S.E.M. Statistical significance was calculated using a two-way ANOVA. **C** Growth curves of U937, MV4-11, and MOLM-13 cells with or without *CTSD* knockdown. Data are presented as the mean ± S.E.M. Statistical significance was calculated using a two-way ANOVA. **D** Schematic of the strategy applied for investigating the role of *CTSD* knockdown in AML progression. **E** Flow cytometry analysis of the percentage of leukemia cells in the PB, BM, and spleen of the indicated groups (*n* = 5 mice per group). hCD45^+^ cells were quantified using FlowJo software. Data are presented as the mean ± S.E.M. Statistical significance was calculated using a two-tailed Student’s *t*-test. **F** Flow cytometry analysis of the percentage of apoptotic cells in the PB, BM, and spleen of indicated groups (*n* = 5 mice per group). Annexin V^+^ cells were quantified using FlowJo software. Data are presented as the mean ± S.E.M. Statistical significance was calculated using a two-tailed Student’s *t*-test.

To investigate the role of CTSD in AML, we first used small interfering RNA (siRNA) to silence CTSD expression in U937, MV4-11, and MOLM-13 leukemia cell lines (Supplementary Fig. 2A). Flow cytometry revealed a significant increase in the percentage of apoptotic cells following *CTSD* knockdown in AML cells (Supplementary Fig. 2B). To rule out the off-target toxicity of *CTSD*-siRNAs, we performed a rescue experiment by reintroducing CTSD into U937 cells transfected with *CTSD*-siRNAs. *CTSD*-siRNA U937 cells induced the apoptosis of leukemia cells, whereas CTSD overexpression effectively reversed this effect, reducing apoptosis in the same context (Supplementary Fig. 2C, D). However, due to the high apoptosis rate ( > 80%), stable cell lines could not be established using siRNA transfection. For in vivo studies, we subsequently generated cell lines stably expressing *CTSD*-shRNAs or *CON*-shRNA (Fig. 2A). Consistent with the *CTSD*-siRNA results, the *CTSD*-shRNA stable cell lines persistently exhibited a higher and relatively stable apoptosis rate (approximately 20–30%) compared to the *CON*-shRNA group (Fig. 2B). In addition, *CTSD* knockdown significantly inhibited the proliferative capacity of U937, MV4-11, and MOLM-13 cells (Fig. 2C). We also evaluated the effects of *CTSD* knockdown in HL60 cells with low CTSD expression. Although *CTSD* knockdown led to reduced proliferation and increased apoptosis in HL60 cells, these effects were less pronounced compared to those observed in monocytic AML cells (Supplementary Fig. 2E–G), indicating the critical role of CTSD in the survival of monocytic AML cells. Furthermore, we explored the role of CTSD in leukemia progression in vivo. U937 cells with and without *CTSD* knockdown were transplanted into NSG mice. After three weeks, leukemia burden was assessed in the peripheral blood (PB), bone marrow (BM), and spleen (Fig. 2D). *CTSD* knockdown led to reduced leukemia cell infiltration and increased apoptosis in the PB, BM, and spleen (Fig. 2E, F). Collectively, these results suggest that CTSD may play a critical role in AML progression, particularly in monocytic AML.

### *CTSD* knockdown in AML cells enhances the TRIM21-mediated ubiquitination and degradation of BCL2, BCL-XL, and MCL1

**Fig. 3:**
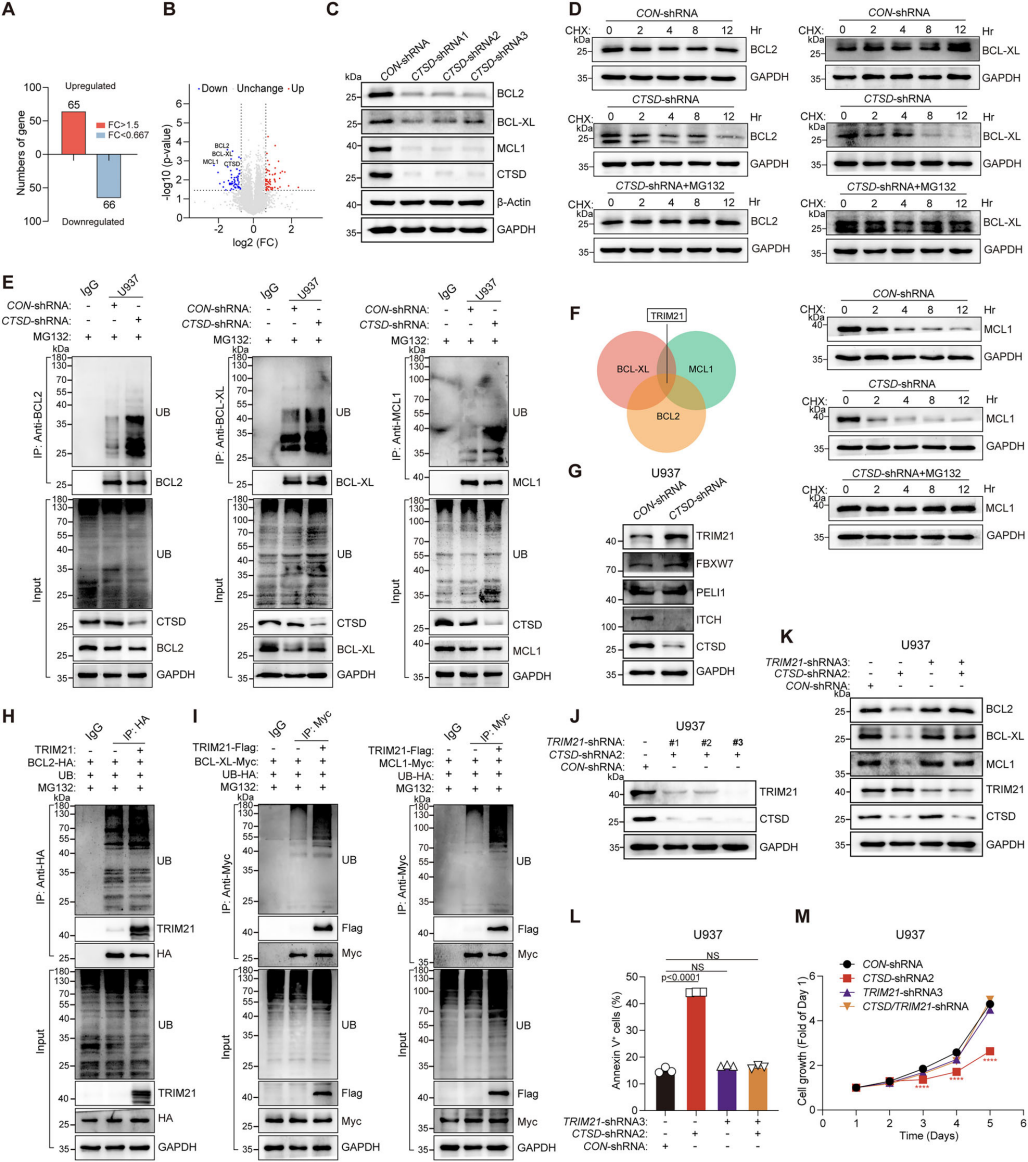
*CTSD* knockdown in AML cells enhances the TRIM21-mediated ubiquitination and degradation of BCL2, BCL-XL, and MCL1. **A** Numbers of upregulated and downregulated differentially expressed proteins (DEPs). **B** Volcano plot of the distribution of DEPs in the proteomic analysis. **C** The protein levels of BCL2, BCL-XL, and MCL1 in U937 cells with or without *CTSD* knockdown were measured using western blotting. **D** Effects of *CTSD* knockdown on the degradation of BCL2, BCL-XL, and MCL1. *CON*- or *CTSD*-shRNA U937 cells were incubated with CHX (20 μg/mL) or CHX plus MG132 (10 μM) for the indicated times, and proteins were detected using western blotting. **E** Effects of *CTSD* knockdown on the ubiquitination of BCL2, BCL-XL, and MCL1 in U937 cells. Protein lysates were immunoprecipitated (IP) with anti-BCL2, anti-BCL-XL, or anti-MCL1 Abs. Ubiquitinated BCL2, BCL-XL, or MCL1 were detected using immunoblotting. **F** Venn diagram showing the common proteins containing TRIM21 identified in the mass spectrometry analysis of BCL2, BCL-XL, and MCL1. **G** The protein levels of TRIM21, FBXW7, PELI1, and ITCH in U937 cells with or without *CTSD* knockdown were detected using western blotting. **H** Ubiquitination of BCL2 after co-transfection with or without E3 ligase plasmid TRIM21 was detected using immunoblotting. **I** Ubiquitination of BCL-XL and MCL1 after co-transfection with or without TRIM21 were detected using immunoblotting. **J** The protein levels of CTSD and TRIM21 in U937 cells transfected with indicated *CTSD*-shRNA2 or *TRIM21*-shRNAs were detected by western blotting. **K** The protein levels of BCL2, BCL-XL, and MCL1 in U937 cells from the indicated shRNA transfection groups were measured using western blotting. **L** Flow cytometric analysis of apoptotic cell proportions in U937 cells from the indicated shRNA transfection groups. Annexin V^+^ cells were quantified using FlowJo software. Data are presented as the mean ± S.E.M. Statistical significance was calculated using a one-way ANOVA. **M** Growth curves of U937 cells from the indicated shRNA transfection groups. Data are presented as the mean ± S.E.M. Statistical significance was calculated using a two-way ANOVA.

To investigate the potential mechanism by which *CTSD* knockdown suppresses AML progression, we conducted a quantitative proteomic analysis to identify changes in protein abundance in U937 cells with or without *CTSD* knockdown. After performing a comparative analysis using Student’s *t*-test, proteins exhibiting a > 1.5-fold change in abundance and *p*-values < 0.05 were considered significantly altered in expression compared to U937-*CON*-shRNA cells. As shown in Fig. 3A, based on these criteria, we identified 131 differentially expressed proteins (DEPs): 65 were upregulated and 66 were downregulated in the *CTSD* knockdown cells. Notably, the protein levels of anti-apoptotic proteins BCL2, BCL-XL, and MCL1 were significantly reduced in the *CTSD* knockdown cells (Fig. 3B). This reduction was further validated by western blot analysis in U937 cells with or without *CTSD* knockdown (Fig. 3C). However, *CTSD* knockdown had no effect on the mRNA expression of *BCL2*, *BCL-XL*, or *MCL1* (Supplementary Fig. 3A), suggesting post-transcriptional regulation. Further analysis revealed that *CTSD* knockdown shortened the half-life of BCL2, BCL-XL, and MCL1, which was reversed by the proteasome inhibitor MG132 (Fig. 3D). These findings indicate that CTSD regulates the stability of these proteins via the ubiquitin-proteasome system (UPS). As protein degradation through the UPS requires E3 ligase-mediated ubiquitin conjugation, we next explored the impact of CTSD on the ubiquitination of these proteins. *CTSD* knockdown markedly increased the ubiquitination of BCL2, as well as BCL-XL and MCL1 (Fig. 3E), while CTSD overexpression reduced their ubiquitination (Supplementary Fig. 3B). We then screened potential E3 ligases mediating the ubiquitination of BCL2, BCL-XL, and MCL1. TRIM21, a RING finger family E3 ubiquitin ligase, was identified via mass spectrometry (MS) as interacting with MCL1 and BCL-XL [36, 37]. Our co-immunoprecipitation mass spectrometry (Co-IP-MS) result also indicated that BCL2 can interact with TRIM21 (Fig. 3F). Previous studies have reported that TRIM21 triggers the ubiquitination and degradation of BCL2 [38], and FBXW7 mediates MCL1 ubiquitination [28]. E3 ligases PELI1 and ITCH are also involved in apoptosis regulation [39–41]. We first examined whether *CTSD* knockdown affected the expression of these E3 ligases. As shown in Fig. 3G, *CTSD* knockdown increased protein level of TRIM21, while having no effect on the expression of FBXW7 or PELI1. Although ITCH level was decreased, this change did not correspond to the reduced protein levels of BCL2, BCL-XL, and MCL1. Notably, *CTSD* knockdown did not alter the mRNA levels of *TRIM21*, *FBXW7*, *PELI1*, or *ITCH*, but specifically increased the protein stability of TRIM21 (Supplementary Fig. 3C, D). Further analysis revealed that among the tested E3 ligases, only TRIM21 promoted the ubiquitination of BCL2 (Fig. 3H and Supplementary Fig. 3E). Moreover, TRIM21 was found to interact with BCL-XL and MCL1, and its overexpression similarly enhanced their ubiquitination (Fig. 3I), suggesting that TRIM21 serves as a common E3 ligase for BCL2, BCL-XL, and MCL1. To further validate that CTSD regulates the expression of those anti-apoptotic proteins via TRIM21, we performed rescue experiments by simultaneously knocking down *TRIM21* in *CTSD*-knockdown U937 cells (Fig. 3J and Supplementary Fig. 3F). *CTSD* knockdown reduced both the protein levels and half-life of BCL2, BCL-XL, and MCL1. However, co-knockdown of *TRIM21* restored their expression and stability (Fig. 3K and Supplementary Fig. 3G). Functionally, *CTSD* knockdown suppressed cell proliferation and promoted apoptosis, while additional *TRIM21* knockdown reversed these effects, enhancing proliferative capacity and reducing apoptosis (Fig. 3L, M). We also detected protein levels of TRIM21 in eight AML cell lines and found that its expression was not consistent with the differential CTSD expression (Supplementary Fig. 3H and Fig. 1E). Furthermore, no significant correlation was observed between the baseline expression levels of CTSD and TRIM21 (Supplementary Fig. 3I). Taken together, these findings suggest that *CTSD* knockdown upregulates TRIM21 protein, which subsequently increases the ubiquitination of BCL2, BCL-XL, and MCL1 and accelerates their degradation.

### Virtual screening for potential CTSD inhibitors

**Fig. 4:**
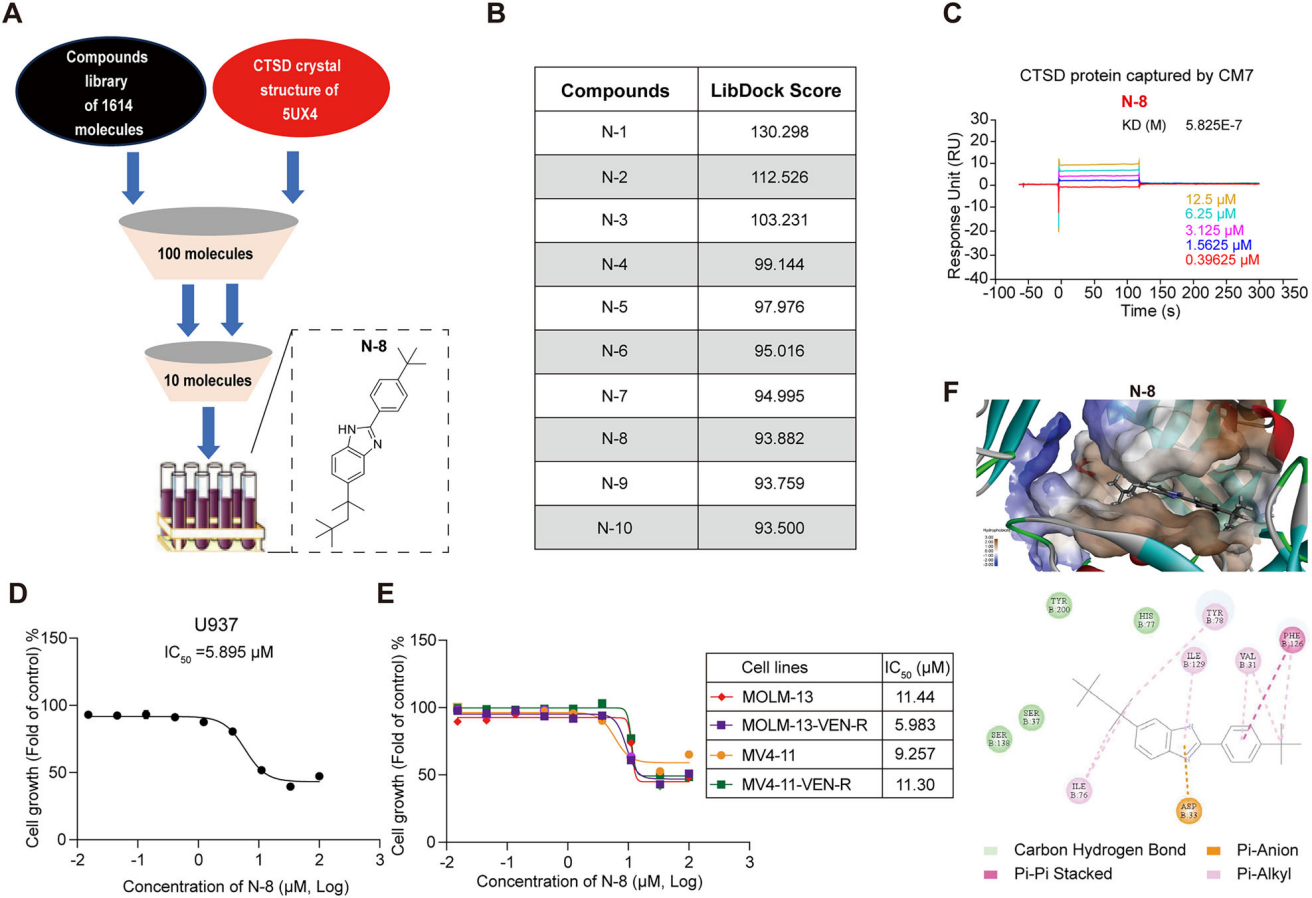
Virtual screening for potential CTSD inhibitors. **A** Screening of library compounds and the structure of **N-8**. **B** Ten compounds Score values. **C** Kinetics of the CTSD and **N-8** interaction determined by surface plasmon resonance (SPR) analysis. **D** Effect of **N-8** on the growth of U937 cells. Data are presented as the mean ± S.E.M. of three independent experiments. **E** Effect of **N-8** on the growth of MOLM-13, MV4-11, and their venetoclax-resistant cells (left) and a summary of IC_50_ values for **N-8** (right). Data are presented as the mean ± S.E.M. of three independent experiments. **F** Conformation of **N-8** in the docking site, 2D interaction between **N-8** and CTSD, and schematic diagram of the action.

Virtual screening, a component of computer-aided drug design, has a crucial role in modern drug development. By simulating the interactions between small-molecule compounds and target proteins, compounds with potential biological activity can be quickly identified. In the development of CTSD inhibitors, virtual screening provides a fast and efficient method to identify novel chemical scaffolds, paving the way for the optimisation and synthesis of new compound series. For this process, we utilized the co-crystallised structure of the CTSD active site and its ligand (PDB: 5UX4) as a reference. We performed molecular docking using a database of 2016 FDA-approved compounds and an internal library consisting of 1614 molecules (Fig. 4A). Using the Libdock module of Discovery Studio 3.0, we screened and identified 821 compounds capable of binding to CTSD. We then selected the top 100 compounds, as ranked by docking score within the binding pocket, and evaluated their chemical structure diversity and the interactions between the ligands and key amino acids in the binding site. Ultimately, we identified 10 high-scoring potential CTSD inhibitors (**N-1**–**N-10**, Fig. 4B). Nine of these virtual screening compounds (**N-1**–**N-7,** **N-9**–**N-10**) were purchased from a commercial supplier (TargetMol, China), whereas **N-8** was synthesised (structure shown in Fig. 4A and Supplementary Fig. 4A). Surface plasmon resonance (SPR) analysis revealed that compounds **N-2,** **N-4**, and **N-8** exhibited significant binding affinity to CTSD (Fig. 4C and Supplementary Fig. 4B). Among these, **N-8** demonstrated superior inhibitory activity in U937 cells compared to **N-2** and **N-4** (Fig. 4D and Supplementary Fig. 4C). Further evaluation confirmed that **N-8** effectively inhibited proliferation of MV4-11 and MOLM-13 cells with IC_50_ values in the micromolar range and exerted similar effects in venetoclax-resistant AML cells (MOLM-13-VEN-R and MV4-11-VEN-R) (Fig. 4E). Structurally, **N-8** features a novel CTSD inhibitor scaffold that is entirely distinct from previous inhibitor structures. We analyzed the interactions between the active compound **N-8** and the residues within the CTSD active site. Figure 4F illustrated potential conformations of the binding between **N-8** and CTSD. **N-8** can form various interactions with CTSD, including ‘alkyl’ and ‘pi–anion’ interactions (shown by orange lines), ‘pi–alkyl’ interactions (shown by pink lines) and ‘pi–pi stacked’ interactions (shown by purple lines). The benzimidazole scaffold interacts with the key binding residues Ile129 and Asp33, whereas the two alkyl portions also interact with amino acids within CTSD. The tert-butyl group forms alkyl-type interactions with Val31 and Phe126, whereas the 2,4,4-trimethylpentan-2-yl group interacts with Ile76 and Tyr78. These findings suggest that modifying the alkyl structure while maintaining the benzimidazole scaffold could significantly impact the compound’s activity. The binding models of compounds **N-1–N-7,** **N9–N10** with CTSD were also shown in Supplementary Fig. 4D. Given the structural novelty and favorable binding affinity of **N-8**, we selected it as the lead compound for subsequent activity evaluation and mechanistic studies.

### N-8 destabilizes BCL2, BCL-XL, and MCL1 to inhibit AML

**Fig. 5:**
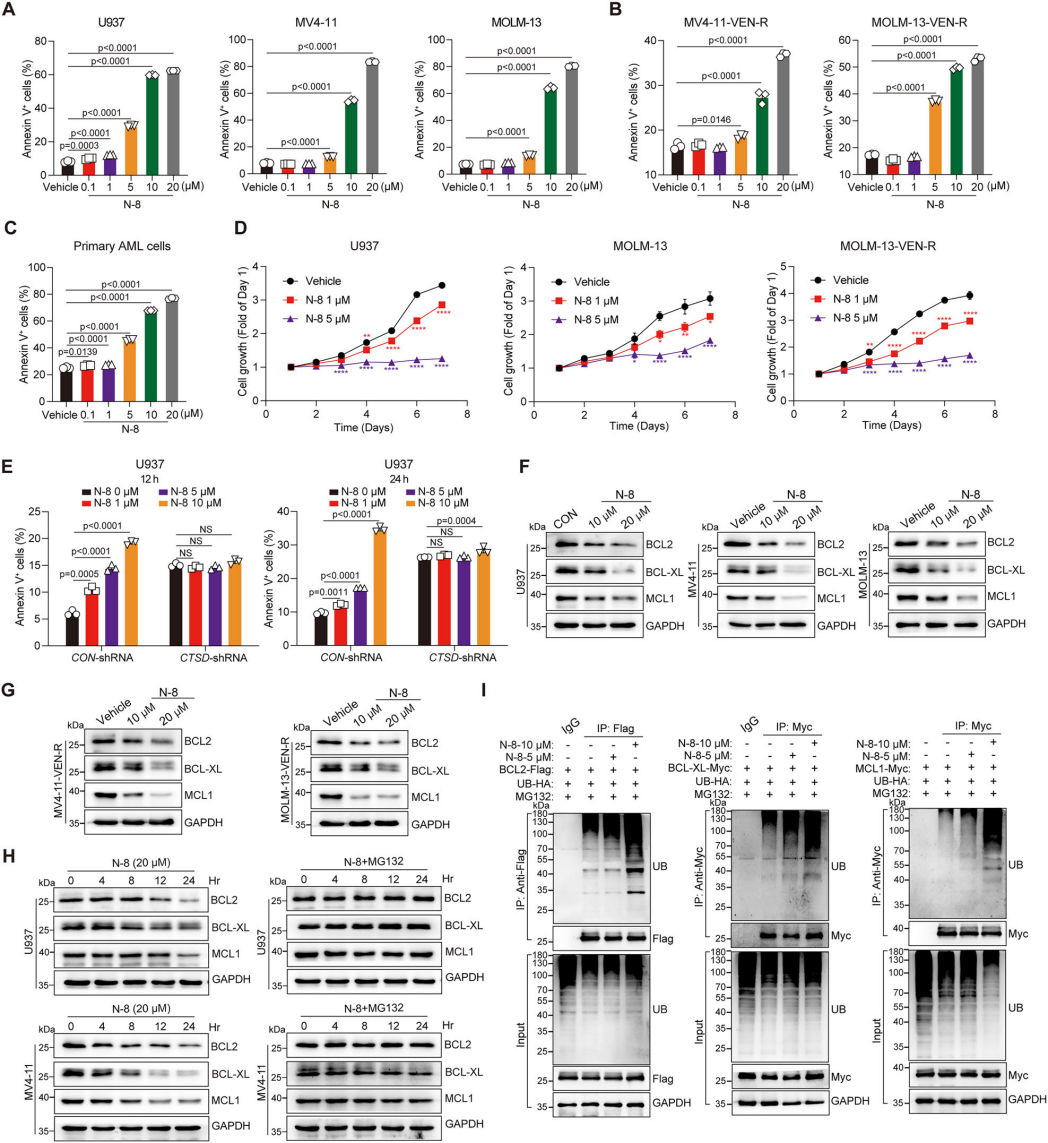
N-8 destabilizes BCL2, BCL-XL, and MCL1 to inhibit AML. Effect of **N-8** on apoptosis of U937, MV4-11, MOLM-13 (**A**), MV4-11-VEN-R, MOLM-13-VEN-R (**B**), and primary AML cells (**C**). Cells were treated with the indicated concentrations of **N-8**, evaluated after 48 h and stained with Annexin V/PI. The percentage of Annexin V^+^ cells was calculated using FlowJo software. Data are presented as the mean ± S.E.M. Statistical significance was calculated using a one-way ANOVA. **D** Effect of **N-8** on proliferation of U937, MOLM-13, and MOLM-13-VEN-R cells. Data are presented as the mean ± S.E.M. Statistical significance was calculated using a two-way ANOVA. **E** Effect of **N-8** on apoptosis of U937 cells with or without *CTSD* knockdown. U937 cells with or without *CTSD* knockdown were treated with the indicated concentrations of **N-8**, evaluated after 12 or 24 h and stained with Annexin V/PI. The percentage of Annexin V^+^ cells was calculated using FlowJo software. Data are presented as the mean ± S.E.M. Statistical significance was calculated using a two-way ANOVA. Effect of **N-8** on the protein levels of BCL2, BCL-XL, and MCL1 in U937, MV4-11, MOLM-13 (**F**), MV4-11-VEN-R, and MOLM-13-VEN-R cells (**G**). Cells were treated with the indicated concentrations of **N-8** for 24 h, and proteins were detected using western blotting. **H** Effect of **N-8** on the degradation of BCL2, BCL-XL, and MCL1 proteins. U937 and MV4-11 cells were incubated with CHX (20 μg/mL) or CHX plus MG132 (10 μM) for the indicated times, and proteins were detected using western blotting. **I** Effect of **N-8** on the ubiquitination of BCL2, BCL-XL, and MCL1. Lenti-X 293 T cells were transfected with the indicated plasmids for 24 h and treated with the indicated concentrations of **N-8**. After 24 h, ubiquitinated BCL2, BCL-XL, or MCL1 was detected using immunoblotting.

We then evaluated the effects of **N-8** on apoptosis and proliferation in leukemia cells. Flow cytometric analysis revealed that **N-8** induced apoptosis in both AML cells and venetoclax-resistant AML cells in a dose-dependent manner (Fig. 5A, B). Notably, it also showed significant pro-apoptotic activity in primary AML cells (Fig. 5C). In addition, **N-8** markedly inhibited leukemia cell proliferation (Fig. 5D), demonstrating potent anti-AML efficacy in vitro. To evaluate target specificity, we assessed **N-8** in *CTSD*-knockdown cells. Compared with the *CON*-shRNA group, no significant apoptosis was induced in *CTSD*-shRNA U937 cells (Fig. 5E). To avoid potential artifact caused by selection bias, we further tested the effect of **N-8** in HL60 cells, which exhibit low endogenous CTSD expression. HL60 cells displayed minimal sensitivity to **N-8**, with an IC_50_ exceeding 100 μM, and only a modest increase in apoptosis at 10 μM (Supplementary Fig. 5A, B). Notably, this pro-apoptotic activity was also eliminated upon *CTSD* knockdown (Supplementary Fig. 5C). Collectively, these findings indicate that the anti-AML efficacy of **N-8** is CTSD-dependent, effectively inhibiting proliferation and inducing apoptosis in leukemia cells with high CTSD expression.

We next detected the effect of **N-8** on the protein levels, stability, and ubiquitination of BCL2, BCL-XL, and MCL1. **N-8** reduced the expression of these three proteins in both AML cells and venetoclax-resistant AML cells in a dose-dependent manner (Fig. 5F, G), whereas this effect was attenuated in *CTSD* knockdown cells (Supplementary Fig. 5D). In addition, **N-8** shortened the half-life of BCL2, BCL-XL, and MCL1, an effect that was reversed by the proteasome inhibitor MG132 (Fig. 5H), indicating involvement of the ubiquitin–proteasome pathway. Consistently, **N-8** increased the ubiquitination of BCL2, BCL-XL, and MCL1 (Fig. 5I). We further observed that **N-8** slightly upregulated TRIM21 expression and enhanced its interaction with BCL2, BCL-XL, and MCL1 (Supplementary Fig. 5E, F). Notably, the pro-apoptotic effect of **N-8** was abolished upon *TRIM21* knockdown (Supplementary Fig. 5G). These results suggest that **N-8** inhibits AML by promoting TRIM21-mediated degradation of the anti-apoptotic proteins BCL2, BCL-XL, and MCL1.

### N-8 attenuates the progression of AML in vivo

**Fig. 6:**
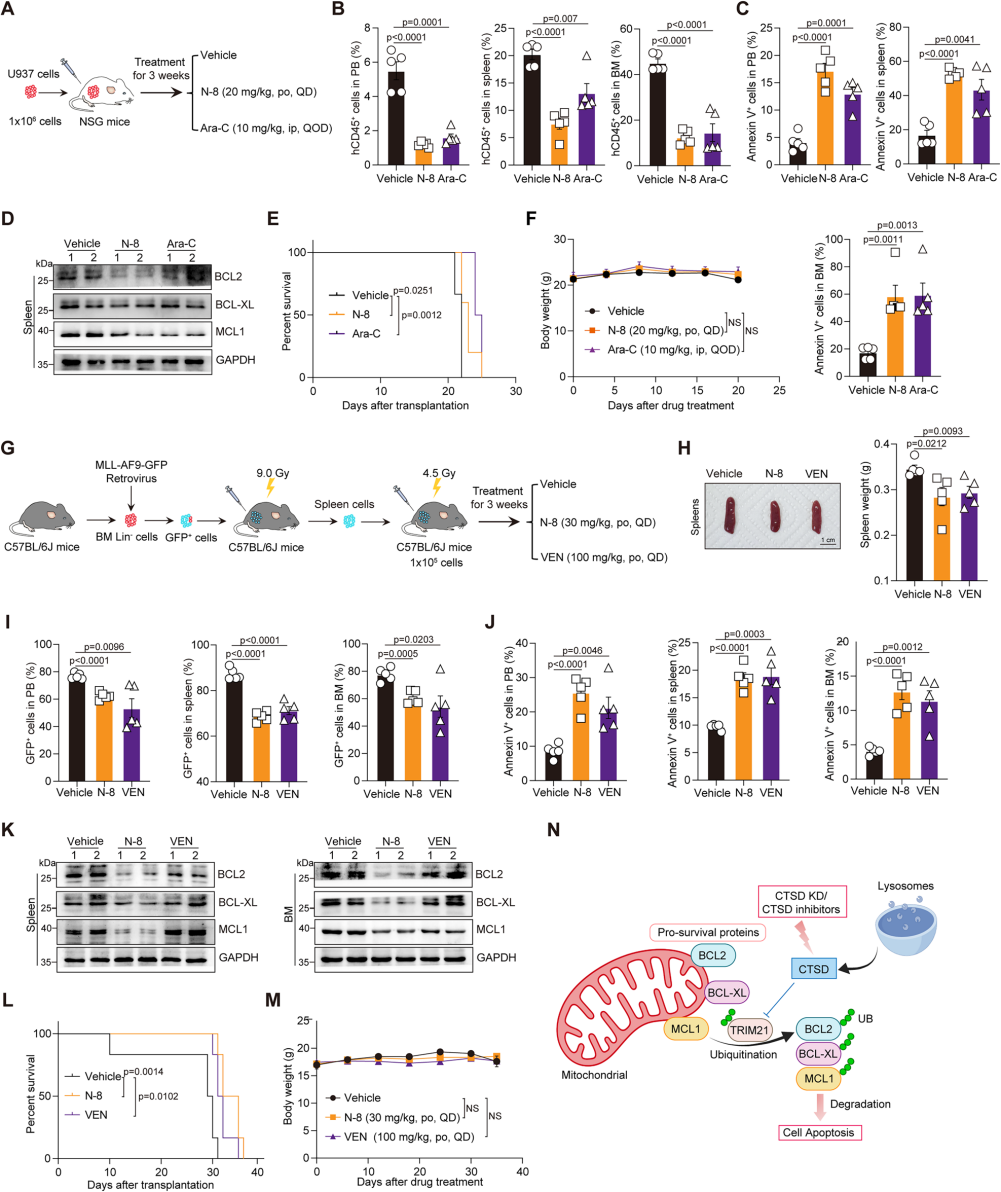
N-8 attenuates the progression of AML in vivo. **A** Schematic strategy for investigating the effect of **N-8** on the progression of AML in mice xenografted with U937 AML cells (AML mice). Ara-C, cytarabine. po, Per Os. ip, Intraperitoneal. QD, Quaque Die. QOD, Quaque Other Die. **B** Flow cytometry analysis of the percentage of leukemia cells in the PB, BM, and spleen of the indicated groups (*n* = 5 mice per group). hCD45^+^ cells were quantified using FlowJo software. Data are presented as the mean ± S.E.M. Statistical significance was calculated using a one-way ANOVA. **C** Flow cytometry analysis of the percentage of apoptotic cells in the PB, BM, and spleen of the indicated groups (*n* = 5 mice per group). Annexin V^+^ cells were quantified using FlowJo software. Data are presented as the mean ± S.E.M. Statistical significance was calculated using a one-way ANOVA. **D** The protein levels of BCL2, BCL-XL, and MCL1 in the spleen of AML mice treated with or without **N-8** were detected using western blotting. **E** Kaplan-Meier survival curves for the indicated mice (*n* = 5 mice per group). Statistical significance was calculated using a two-sided log-rank test. **F** The body weight curves of AML mice treated with the indicated agents (*n* = 5 mice per group). Data are presented as the mean ± S.E.M. Statistical significance was calculated using a two-way ANOVA. **G** Schematic of the strategy used to investigate the effect of **N-8** on the progression of AML in the *MLL-AF9* leukemia model. VEN, venetoclax. po, Per Os, QD, Quaque Die. **H** Representative spleen images (left) and statistical analysis of spleen weights (right) obtained from mice with the indicated treatment. Scale bar, 1 cm. **I** Flow cytometry analysis of the percentage of leukemia cells in the PB, BM, and spleen of the indicated groups (*n* = 5 mice per group). GFP^+^ cells were quantified using FlowJo software. Data are presented as the mean ± S.E.M. Statistical significance was calculated using a one-way ANOVA. **J** Flow cytometry analysis of the percentage of apoptotic cells in the PB, BM, and spleen of the indicated groups (*n* = 5 mice per group). Annexin V^+^ cells were quantified using FlowJo software. Data are presented as the mean ± S.E.M. Statistical significance was calculated using a one-way ANOVA. **K** The protein levels of BCL2, BCL-XL, and MCL1 in the spleen and BM of AML mice treated with or without **N-8** was detected using western blotting. **L** Kaplan-Meier survival curves for the indicated mice (*n* = 5 mice per group). Statistical significance was calculated using a two-sided log-rank test. **M** The body weight curves of leukemia mice treated with the indicated agents (*n* = 5 mice per group). Data are presented as the mean ± S.E.M. Statistical significance was calculated using a two-way ANOVA. **N** Schematic diagram illustrating the mechanism by which CTSD inhibition induces cell apoptosis.

We then investigated the therapeutic effect of **N-8** in mice xenografted with U937 AML cells (AML mice), which were resistant to venetoclax treatment [7] (Fig. 6A and Supplementary Fig. 6A). After 3 weeks of treatment, **N-8** significantly reduced leukemia cell infiltration in the PB, BM, and spleen (Fig. 6B) and increased the percentage of apoptotic cells (Fig. 6C). Consistent with its pro-apoptotic activity, the protein levels of BCL2, BCL-XL, and MCL1 in the spleen were lower in **N-8**-treated mice compared to the vehicle group (Fig. 6D). Additionally, **N-8** prolonged survival (Fig. 6E) with no obvious side effects, as demonstrated by stable body weight throughout the treatment period (Fig. 6F). Notably, we found that the therapeutic effect of **N-8** was comparable to that of Ara-C treatment (Fig. 6B–F).

Moreover, we established an *MLL-AF9* leukemia model to further explore the in vivo therapeutic effect of **N-8** (Fig. 6G). Venetoclax, which has high sensitivity in the *MLL-AF9* model, was used as the positive control drug. **N-8** treatment reduced spleen weight (Fig. 6H) and improved survival in AML mice (Fig. 6L). After 3-week **N-8** treatment, GFP^+^ leukemia cells in PB, BM, and spleen were greatly reduced (Fig. 6I), while the percentage of apoptotic AML cells increased (Fig. 6J). Additionally, **N-8** treatment reduced BCL2, BCL-XL, and MCL1 expression in spleen and BM cells, whereas venetoclax treatment had no significant effect (Fig. 6K). Finally, **N-8** treatment showed no apparent side effects, as demonstrated by stable body weight and unchanged levels of serum alanine transaminase (ALT), aspartate transferase (AST), cholesterol (CHO), triglycerides (TG), glucose (GLU), UREA, and creatinine (CRE) in xenografted mice (Fig. 6M and Supplementary figure 6B). In addition, given that BCL-XL inhibitors are known to cause thrombocytopenia due to their toxic effects on platelets [42], we monitored peripheral blood platelet counts during a 7-day **N-8** treatment course. The results showed that platelet levels remained unaffected (Supplementary Fig. 6C), suggesting low hematopoietic toxicity and a favourable in vivo safety profile.

## Discussion

CTSD has been reported to play a key role in regulating cancer invasion, migration, metastasis, and angiogenesis and serve as a promising target for the therapy of solid tumors [43–46]. However, the role of CTSD in haematological malignancies remains unclear. In this study, we show that CTSD is highly expressed in AML, especially in the monocytic subtype, and that high CTSD expression is associated with unfavourable prognosis. We show that *CTSD* knockdown in leukemia cells suppresses cell proliferation and the anti-apoptotic effects in vitro and alleviates AML progression in vivo.

In solid tumors, CTSD is required for the migration and invasion of gastric and breast cancer cells [45, 46]. However, its role in cell death remains unclear. Studies have shown that the effect of CTSD on cell death was dependent on both the stimulators used and the cell context. Oliveira et al. found that CTSD promoted the autophagy-independent degradation of damaged mitochondria to protect colorectal cancer cells from acetate-induced apoptosis [47]. Han et al. reported that CTSD activated autophagy in cancer cells, thereby inhibiting oxidative stress-induced cell death [48]. However, in other studies, CTSD acted as a sensitiser, increasing cancer cell death following treatment with chemotherapy drugs [49, 50]. Our study found that *CTSD* knockdown promoted apoptosis in leukemia cells by inducing the degradation of the anti-apoptosis proteins BCL2, BCL-XL, and MCL1 (Fig. 6N). This finding agrees with Seung et al., who reported that CTSD inhibition enhanced anticancer drug-induced apoptosis through RNF183-mediated destabilisation of BCL-XL [17]. CTSD is found mainly in low pH environments, such as lysosomes, but its localization can vary [51]. For example, CTSD is activated under the acidic conditions of the lysosome and, under specific conditions, may translocate from the lysosome to the cytoplasm. Moreover, Naoki et al. reported that CTSD harbours a mitochondrial targeting sequence and is localized in the mitochondria [52]. Therefore, the role of CTSD in cell death may depend on its localization, cellular state, and environmental conditions. While our findings establish CTSD’s critical role in AML cell survival, its clinical prognostic value requires further validation. The absence of detailed clinical annotations (e.g., age, cytogenetic risk, or treatment history) in public databases such as GEPIA limits our ability to control for potential confounding factors. Future studies combining mechanistic insights with comprehensively annotated clinical cohorts are needed to determine CTSD’s potential as an independent prognostic biomarker in AML.

The TRIM family, known for its E3 ubiquitin ligase activity, mediates the ubiquitination and degradation of various proteins [53]. Chiharu Ishikawa et al. reported that TRIM21 is overexpressed in AML and supports cell survival and proliferation [54]. Stefanie Göllner et al. found that TRIM21 may induce the ubiquitination and degradation of histone methyltransferase EZH2, contributing to drug resistance [55]. TRIM31, another member of the TRIM family, is also upregulated in AML, and its knockdown inhibits cell proliferation and induces apoptosis [56]. In contrast, Kai Zhang et al. found that TRIM31 may play a different role in regulating the degradation of CDK8, and its knockout accelerates *MLL-AF9* progression in vivo [57]. These studies demonstrate the critical role of the TRIM protein family in AML. The various functions of TRIM family members, however, may arise from their target proteins. Our research indicates that TRIM21 acts as a common E3 ubiquitin ligase for BCL2, BCL-XL, and MCL1, and its elevated expression promotes apoptosis in AML cells.

To date, the most clinically promising targeted agent for AML treatment is the BCL2 inhibitor venetoclax. Venetoclax, when combined with standard chemotherapy, exhibits impressive efficacy in patients with AML [58]. However, approximately 30% of patients with AML are resistant to venetoclax-based regimens. The main mechanisms of venetoclax resistance in AML include the elevation levels of MCL1 and BCL-XL, *BCL2* mutation, and *TP53* aberrations [59]. Our study found that CTSD inhibition promoted the degradation of MCL1, BCL-XL, and BCL2, which could help overcome venetoclax resistance in AML. We identified **N-8** as a potent small-molecule drug targeting CTSD, which resulted in excellent therapeutic effects on AML and venetoclax-resistant AML in vitro and in vivo, offering a potential strategy for their treatment.

In recent years, several peptide and non-peptide inhibitors of CTSD have been emerged. Despite exhibiting strong aspartic proteases inhibitory activity, their poor cell permeability, instability in microsomal assays, and broad-spectrum inhibitory activity limit their application and further development [60–62]. This study identifies **N-8** through screening as a potential small-molecule inhibitor targeting CTSD. **N-8** can be considered a novel anticancer scaffold (2-phenyl-1H-benzo[d]imidazole) that offers promise as a lead compound for drug development. Moreover, **N-8** may act as a valuable chemical biology tool to advance research on CTSD-targeted anticancer therapies. While our findings demonstrate **N-8**’s specificity for CTSD in *CTSD*-knockdown AML cells—evidenced by a reduced pro-apoptotic effect upon CTSD suppression—several questions remain. The specificity of **N-8** to CTSD should be further detected through enzyme activity assay. Moreover, the mechanism through which **N-8** targets CTSD requires further investigation. Although short-term toxic effects of **N-8** were not obvious in AML mice, the long-term toxicity, physicochemical stability, circulating plasma half-life, and target specificity of **N-8** in normal proliferating tissues should be carefully evaluated.

In conclusion, our study proposes CTSD as a novel therapeutic target for the treatment of AML, particularly in overcoming drug resistance. Targeting CTSD leads to the remission of AML by promoting the ubiquitination and degradation of anti-apoptotic proteins. We identified a small-molecule CTSD inhibitor, which demonstrated promising efficacy both in vitro and in vivo, showing potential for further development.

## Supplementary information

supplementary information

Original Western blots

## Data Availability

The quantitative proteomics data generated during this study have been deposited to the ProteomeXchange Consortium (http://proteomecentral.proteomexchange.org) via the iProX partner repository with the dataset identifier PXD067117.
