# Supplementary material for: Cathepsin D promotes acute myeloid leukemia progression through stabilization of the anti-apoptotic proteins
Source: Cell Death Dis. 2025 Aug 12;16(1):611. doi: 10.1038/s41419-025-07949-7 (PMC12343803; doi:10.1038/s41419-025-07949-7)
Supplement: Supplementary file 1 — supplementary information [file 41419_2025_7949_MOESM1_ESM.pdf]

Supplementary Information

Cathepsin D promotes acute myeloid leukemia progression through stabilization of the anti-apoptotic proteins

Running title: Cathepsin D Stabilizes Anti-Apoptotic Proteins in Acute Myeloid Leukemia

Huimin Jiang[1*], Yongjian Wang[1*], Churan Wang[2], Lu Yang[3], Shujuan Wang[3], Feng Wang[1], Situ Xue[1], Zhuan Zhang[1], Haigen Fu[1], Ting Dong[1], Jian Yuan[4], Zhuorong Li[1✉], Ke Li[1✉].

[1]State Key Laboratory of Bioactive Substance and Function of Natural Medicines, NHC Key Laboratory of Biotechnology of Antibiotics, Institute of Medicinal Biotechnology, Chinese Academy of Medical Sciences and Peking Union Medical College, Beijing, China.

[2]Shanghai Institute of Hematology, Blood and Marrow Transplantation Center, Collaborative Innovation Center of Hematology, Department of Hematology, Ruijin Hospital, Shanghai Jiao Tong University School of Medicine, Shanghai, China.

[3]Department of Hematology, The First Affiliated Hospital of Zhengzhou University, Zhengzhou, China.

[4]State Key Laboratory of Cardiology and Research Center for Translational Medicine, Shanghai East Hospital, Tongji University School of Medicine, Shanghai, China.

*These authors contributed equally to this work.

✉E-mail: like1986@163.com; lizhuorong@imb.pumc.edu.cn.

The authors declare no competing interests.

Contents of Supplementary Information

Supplementary Figure 1 4

Supplementary Figure 2 5

Supplementary Figure 3 7

Supplementary Figure 4 10

Supplementary Figure 5 12

Supplementary Figure 6 14

Chemistry supplementary information 15

1. ^{1}H-NMR spectra and MS data of compound **1** 15
2. ^{1}H-NMR spectra and MS data of compound **2** 16
3. ^{1}H-NMR spectra and MS data of compound **3** 17
4. ^{1}H- and ^{3}C-NMR spectra, HRMS, and HPLC data of compound **N-8** 18
5. The structures of reagents 2-chloroacetamide 19
6. The structures of reagents 4-(tert-butyl)benzaldehyde 19

Supplementary Table 1 21

Supplementary Table 2 22

Supplementary Table 3 23

Supplementary methods 24

Quantitative proteomic analysis 24

Chemistry 24

Surface plasmon resonance (SPR) analysis 28

Supplementary Figure 1

A

Cell lines	Gene mutations
KG1	Remain uncertain
Kasumi	*RUNX1-RUNX1T1* fusion
HL60	*P53* deletion
NB4	*PML-RARA* fusion
OCI-AML3	*DNMT3A* mutation, *NPM1* gene mutation type A
U937	*PICALM-MLLT10* fusion, *PTEN, PTPN11, and WT1* mutation
MOLM-13	*FLT3-ITD* fusion
MV4-11	*FLT3-ITD*, *KMT2A/MLLT2 (AF4)* fusion

B

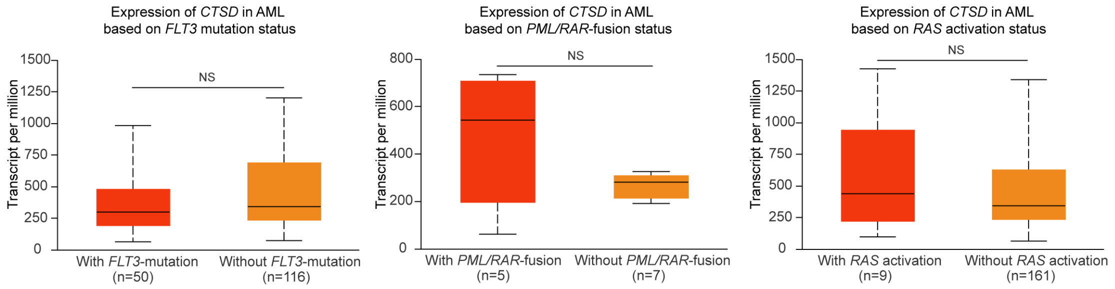

Supplementary Figure 1. High CTSD expression is associated with poor prognosis in patients with AML.

(A) Overview of genetic mutations in the eight AML cell lines.

(B) The mRNA levels of *CTSD* in AML patients with or without *FLT3*, *PML-RARA*, or *RAS* mutations were analyzed using the UALCAN database.

Supplementary Figure 2

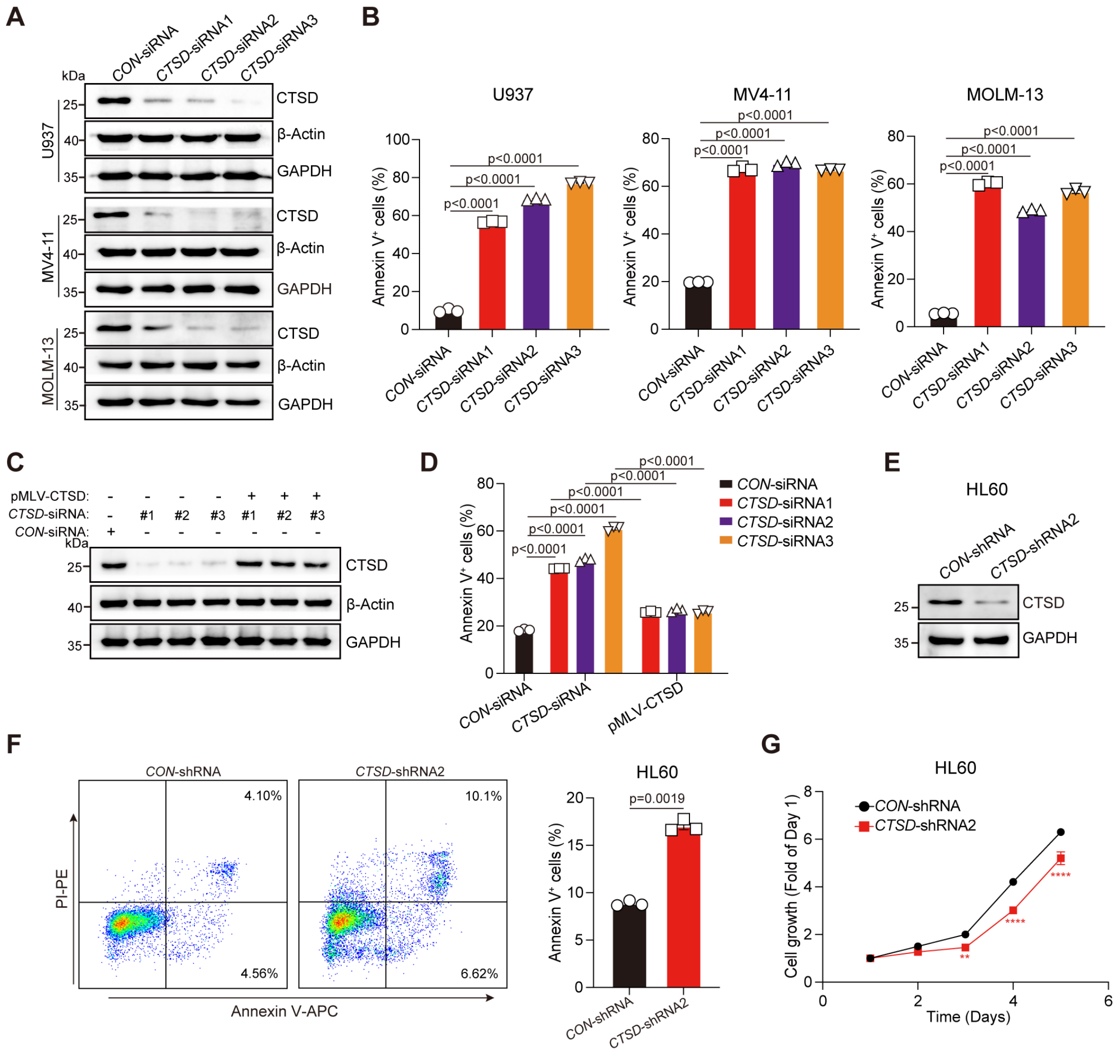

Supplementary Figure 2. CTSD enhances the proliferation and survival of AML cells to promote AML progression.

(A) The protein levels of CTSD in U937, MV4-11, and MOLM-13 cells with *CON*- or *CTSD*-siRNA detected using western blotting.

(B) Flow cytometric analysis of apoptotic cell proportions in U937, MV4-11, and MOLM-13 cells with *CON*- or *CTSD*-siRNA. Annexin V$^+$ cells were quantified using FlowJo software. Data are presented as the mean ± S.E.M. Statistical significance was calculated using a one-way ANOVA.

(C) The protein level of CTSD in U937 cells transfected with control siRNA (*CON*-

siRNA), three CTSD-targeting siRNAs (*CTSD*-siRNA #1-3), or CTSD overexpression plasmid (pMLV-CTSD) was detected using Western blotting. β-Actin and GAPDH were used as internal controls.

(D) Flow cytometric analysis of apoptotic cell proportions in U937 cells from the indicated groups. Annexin V^+ cells were quantified using FlowJo software. Data are presented as the mean ± S.E.M. Statistical significance was calculated using one-way ANOVA or two-way ANOVA.

(E) The protein level of CTSD in HL60 cells with or without *CTSD*-shRNA was detected using western blotting.

(F) Representative two-dimensional FACS plots (left) and flow cytometric analysis of apoptotic cell proportion in HL60 cells with or without *CTSD* knockdown (right). Annexin V^+ cells were quantified using FlowJo software. Data are presented as the mean ± S.E.M. Statistical significance was calculated using a two-tailed Student's t-test.

(G) Growth curves of HL60 cells with or without *CTSD* knockdown. Data are presented as the mean ± S.E.M. Statistical significance was calculated using a two-way ANOVA.

Supplementary Figure 3

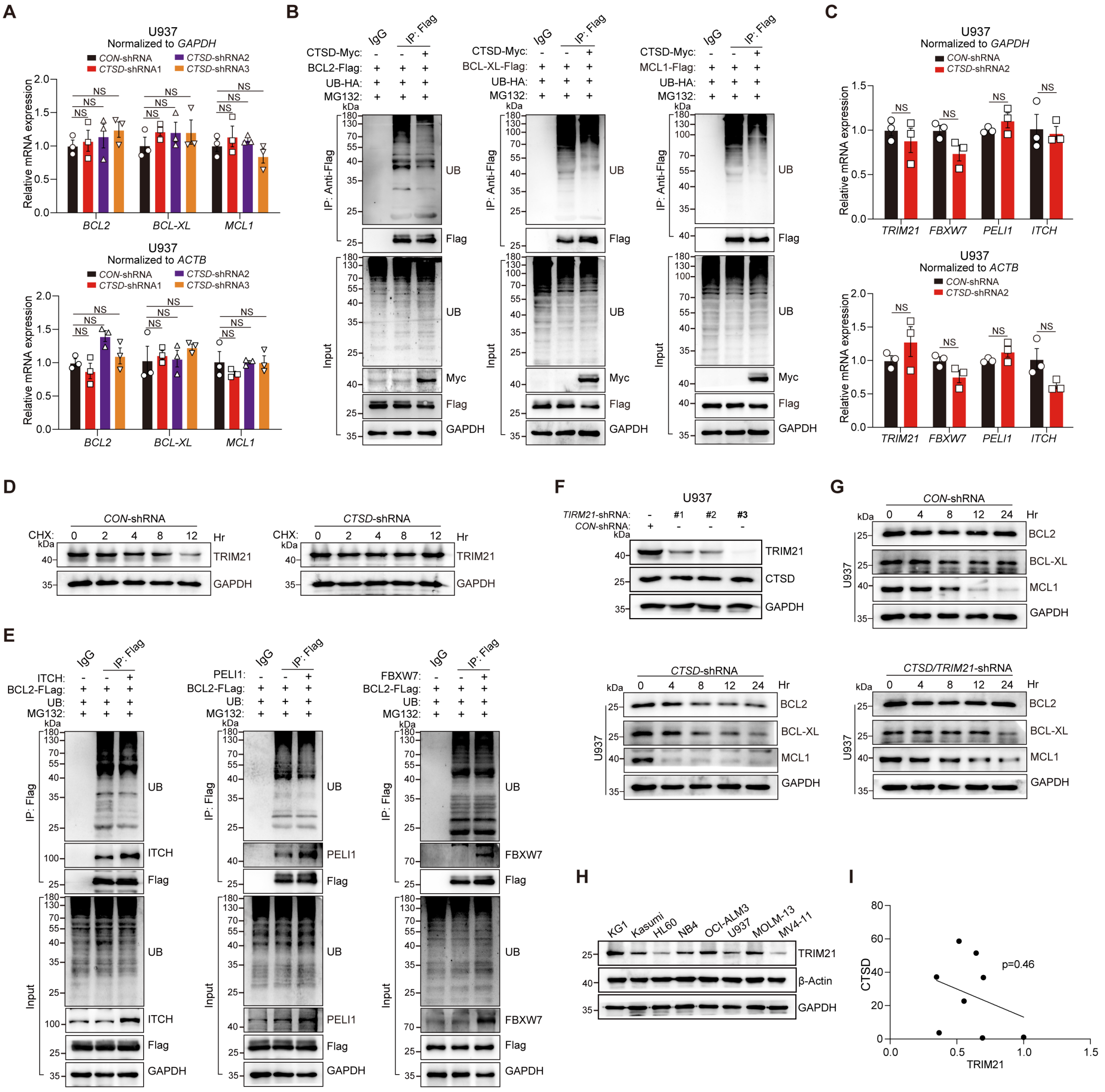

Supplementary Figure 3. *CTSD* knockdown in AML cells enhances the TRIM21-mediated ubiquitination and degradation of BCL2, BCL-XL, and MCL1.

(A) The relative mRNA levels of *BCL2*, *BCL-XL,* and *MCL1* in U937 cells with or without *CTSD* knockdown were detected by quantitative real-time PCR, normalized to *GAPDH* (up) and *ACTB* (down). Data are presented as the mean ± S.E.M. Statistical significance was calculated using a two-way ANOVA.

(B) Effect of CTSD overexpression on the ubiquitination of BCL2, BCL-XL, and MCL1. Lenti-X 293T cells were transfected with the indicated plasmids for 48 hours. Protein lysates were immunoprecipitated (IP) with an anti-Flag Ab. Ubiquitinated BCL2, BCL-

XL, and MCL1 were detected using immunoblotting.

(C) The relative mRNA levels of *TRIM21*, *FBXW7, PELI1*, and *ITCH* in U937 cells with or without *CTSD* knockdown were detected by quantitative real-time PCR, normalized to *GAPDH* (up) and *ACTB* (down). Data are presented as the mean ± S.E.M. Statistical significance was calculated using a two-way ANOVA.

(D) Effect of *CTSD* knockdown on the degradation of TRIM21. *CON*- or *CTSD*-shRNA U937 cells were incubated with CHX (20 μg/mL) for the indicated times, and proteins were detected using western blotting.

(E) Ubiquitination of BCL2 after co-transfection with or without E3 ligase plasmids ITCH, PELI, and FBXW7 were detected using immunoblotting.

(F) The protein level of TRIM21 in U937 cells transfected with *TRIM21*-shRNAs were detected by western blotting.

(G) Effects of *CTSD* knockdown or in combination with *TRIM21* knockdown on the degradation of BCL2, BCL-XL, and MCL1. *CON*-, *CTSD*-, or *CTSD/TRIM21*-shRNA U937 cells were incubated with CHX (20 μg/mL) for the indicated times, and proteins were detected using western blotting.

(H) The protein levels of TRIM21 in eight AML cell lines were detected by western blotting.

(I) Correlation analysis between CTSD and TRIM21 protein levels across AML cell lines.

Supplementary Figure 4

A

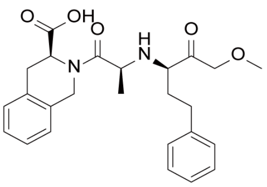

N-1

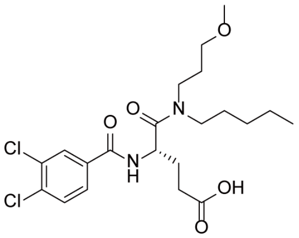

N-2

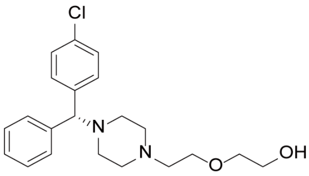

N-3

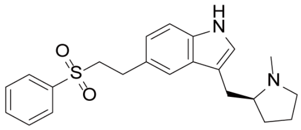

N-4

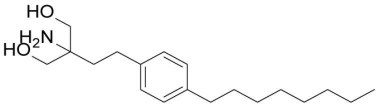

N-5

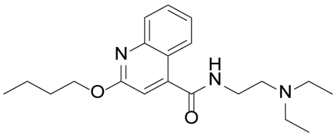

N-6

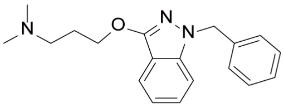

N-7

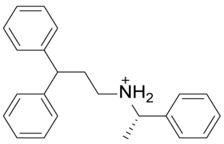

N-9

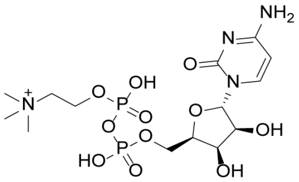

N-10

B

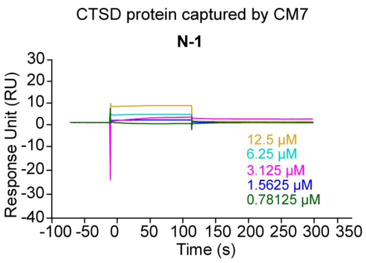

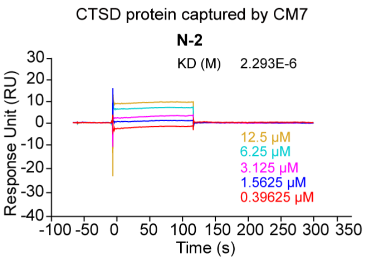

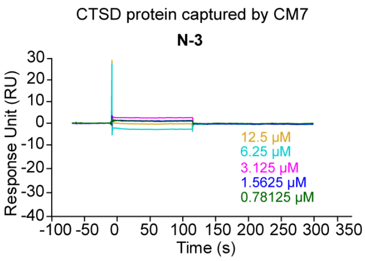

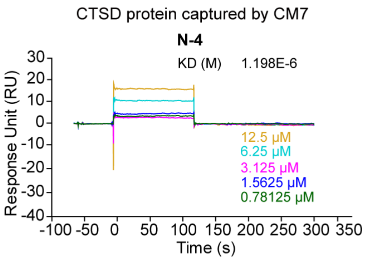

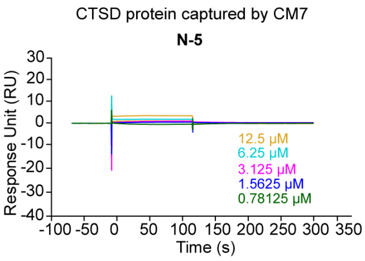

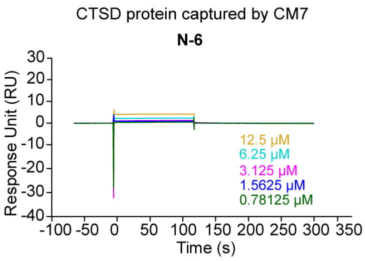

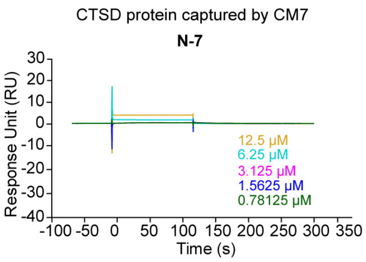

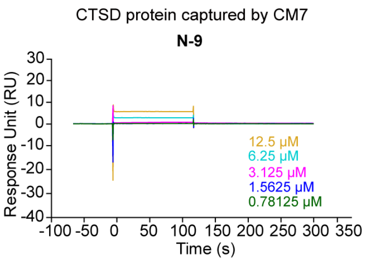

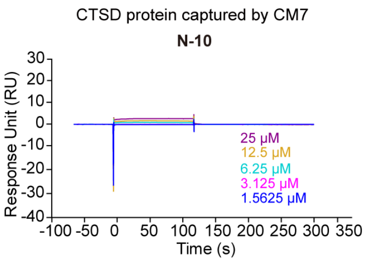

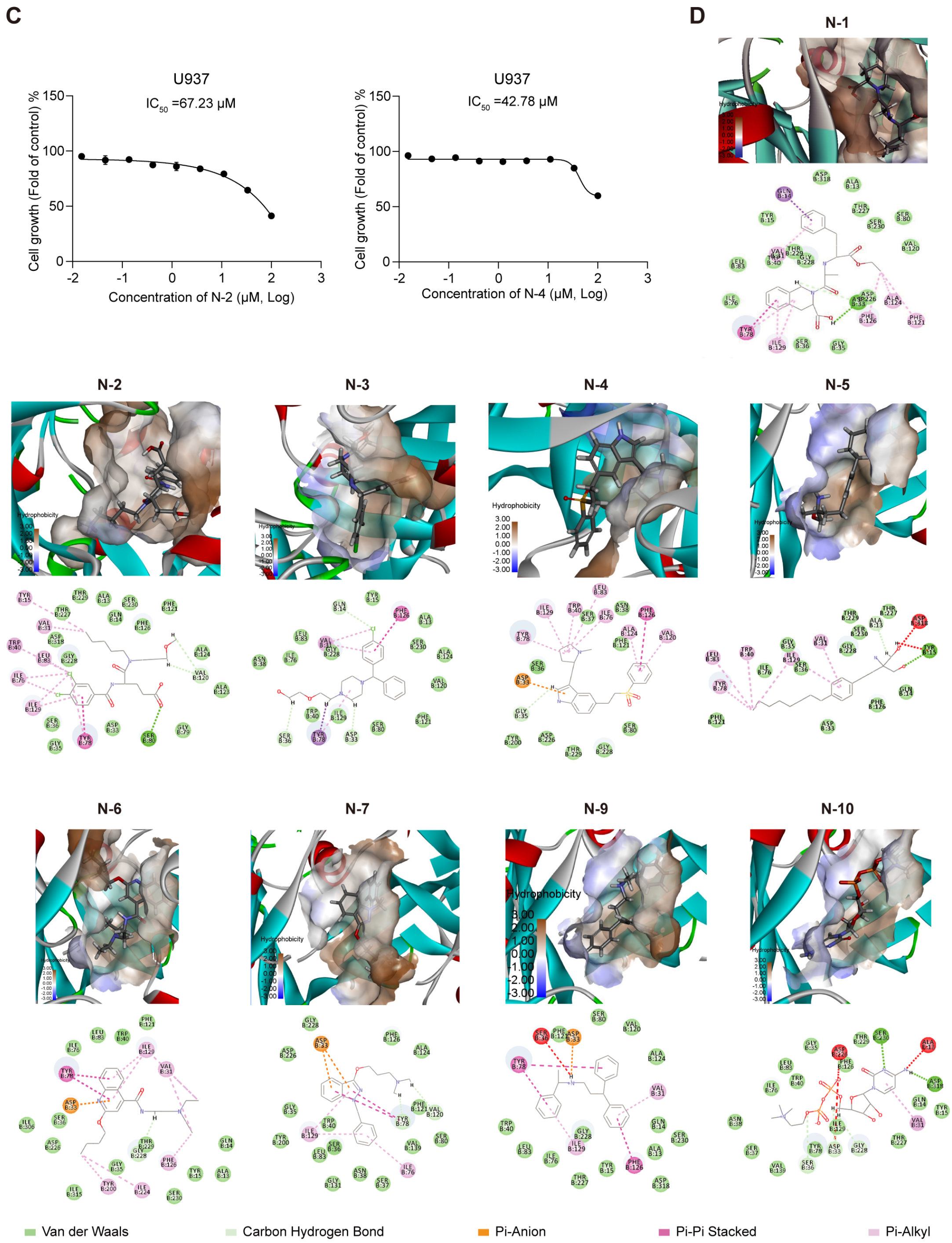

Supplementary Figure 4. Virtual screening for potential CTSD inhibitors.

(A) The structures of compound **N-1**–**N-7**, **N-9**–**N-10**.

(B) Kinetics of the CTSD and compounds (**N-1**–**N-7**, **N-9**–**N-10**) interaction determined by surface plasmon resonance (SPR) analysis.

(C) Effect of **N-2** and **N-4** on the growth of U937 cells. Data are presented as the mean

± S.E.M. of three independent experiments.

(D) Conformation of compounds (**N-1**–**N-7**, **N-9**–**N-10**) in the docking site, 2D interaction between compounds and CTSD, and schematic diagram of the action.

Supplementary Figure 5

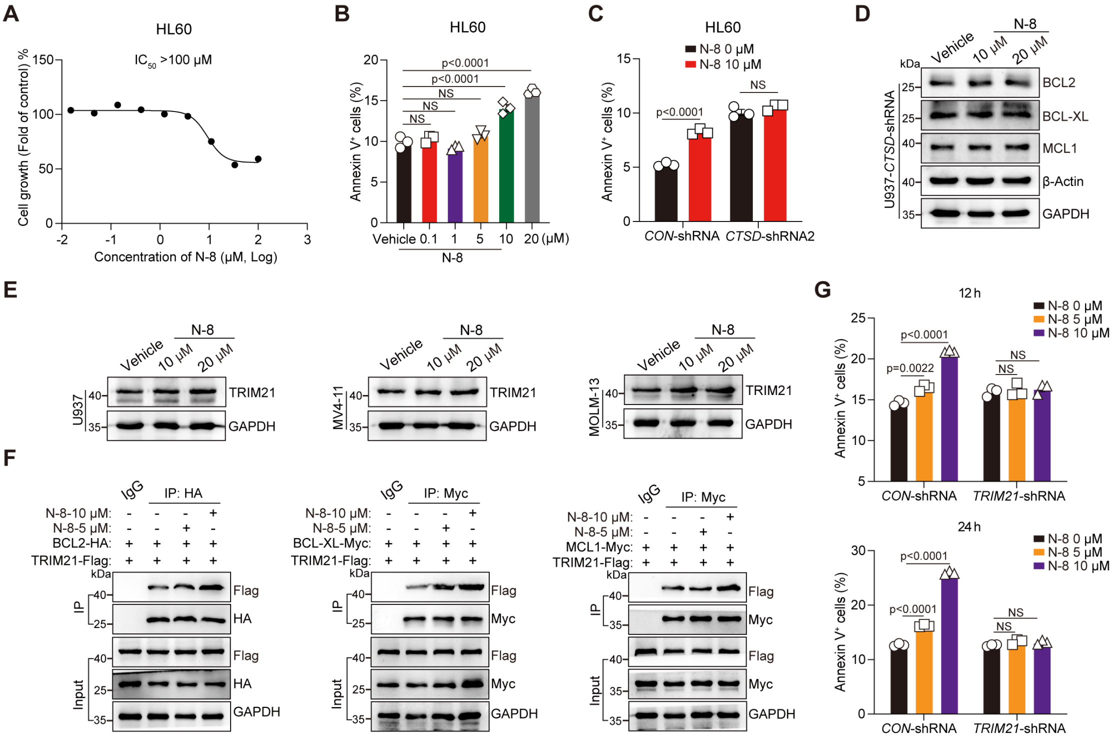

Supplementary Figure 5. N-8 binds with CTSD to destabilize BCL2, BCL-XL and MCL1 and then inhibits AML.

(A) Effect of **N-8** on the growth of HL60 cells. Data are presented as the mean ± S.E.M. of three independent experiments.

(B) Effect of **N-8** on apoptosis of HL60 cells. Cells were treated with the indicated concentrations of **N-8**, evaluated after 48 hours and stained with Annexin V/PI. The percentage of Annexin V^+ cells was calculated using FlowJo software. Data are presented as the mean ± S.E.M. Statistical significance was calculated using a one-way ANOVA.

(C) Effect of **N-8** on apoptosis of HL60 cells with or without *CTSD* knockdown. HL60 cells with or without *CTSD* knockdown were treated with the indicated concentrations of **N-8**, evaluated after 24 hours and stained with Annexin V/PI. The percentage of Annexin V^+ cells was calculated using FlowJo software. Data are presented as the

mean ± S.E.M. Statistical significance was calculated using a two-way ANOVA.

(D) Effect of **N-8** on the protein levels of BCL2, BCL-XL, and MCL1 in U937 cells with *CTSD* knockdown. Cells were treated with the indicated concentrations of **N-8** for 24 hours, and proteins were detected using western blotting.

(E) Effects of **N-8** on the protein levels of TRIM21 in U937, MV4-11, and MOLM-13 cells. Cells were treated with the indicated concentrations of **N-8** for 24 hours, and proteins were detected using western blotting.

(F) Effects of **N-8** on the interaction between TRIM21 and BCL2, BCL-XL, or MCL1 proteins. Lenti-X 293T cells were transfected with the indicated plasmids for 24 hours and treated with the indicated concentrations of **N-8**. After 24 hours, protein lysates were immunoprecipitated (IP) with anti-HA or -Myc Abs. The protein levels of TRIM21-Flag were detected using immunoblotting.

(G) Effect of **N-8** on apoptosis of U937 cells with or without *TRIM21* knockdown. U937 cells with or without *TRIM21* knockdown were treated with the indicated concentrations of **N-8**, evaluated after 12 or 24 hours and stained with Annexin V/PI. The percentage of Annexin V^+ cells was calculated using FlowJo software. Data are presented as the mean ± S.E.M. Statistical significance was calculated using a two-way ANOVA.

Supplementary Figure 6

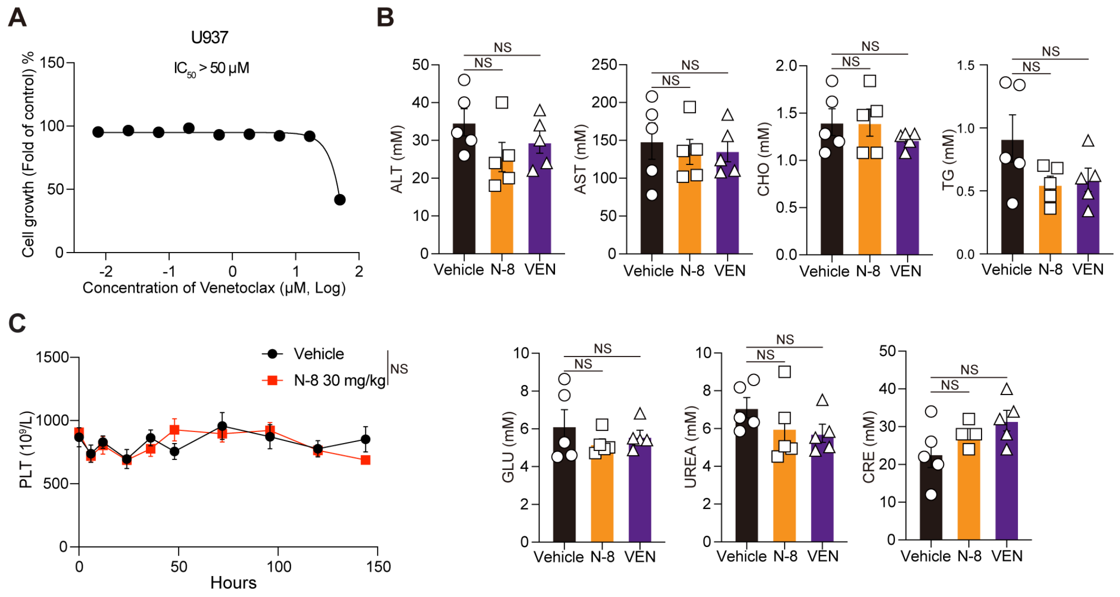

Supplementary Figure 6. N-8 attenuates the progression of AML *in vivo*.

(A) Effect of venetoclax on the growth of U937 cells. Data are presented as the mean ± S.E.M. of three independent experiments.

(B) The serum levels of ALT, AST, CHO, TG, GLU, UREA, or CRE levels in AML mice of the indicated groups (n = 5 mice per group). Data are presented as the mean ± S.E.M. Statistical significance was calculated using a one-way ANOVA. ALT, alanine transaminase. AST, aspartate transferase. CHO, cholesterol. TG, triglycerides. GLU, glucose. CRE, creatinine.

(C) Peripheral blood platelet (PLT) counts in C57BL/6J mice treated with vehicle or **N-8** were administrated by a five-part differential hematology instrument (NIHON KOHDEN Celltac E) for consecutive seven days (n = 5 mice per group). Data are presented as mean ± S.E.M. Statistical significance was calculated using a two-way ANOVA.

Chemistry supplementary information

1. ^{1}H-NMR spectra and MS data of compound **1**

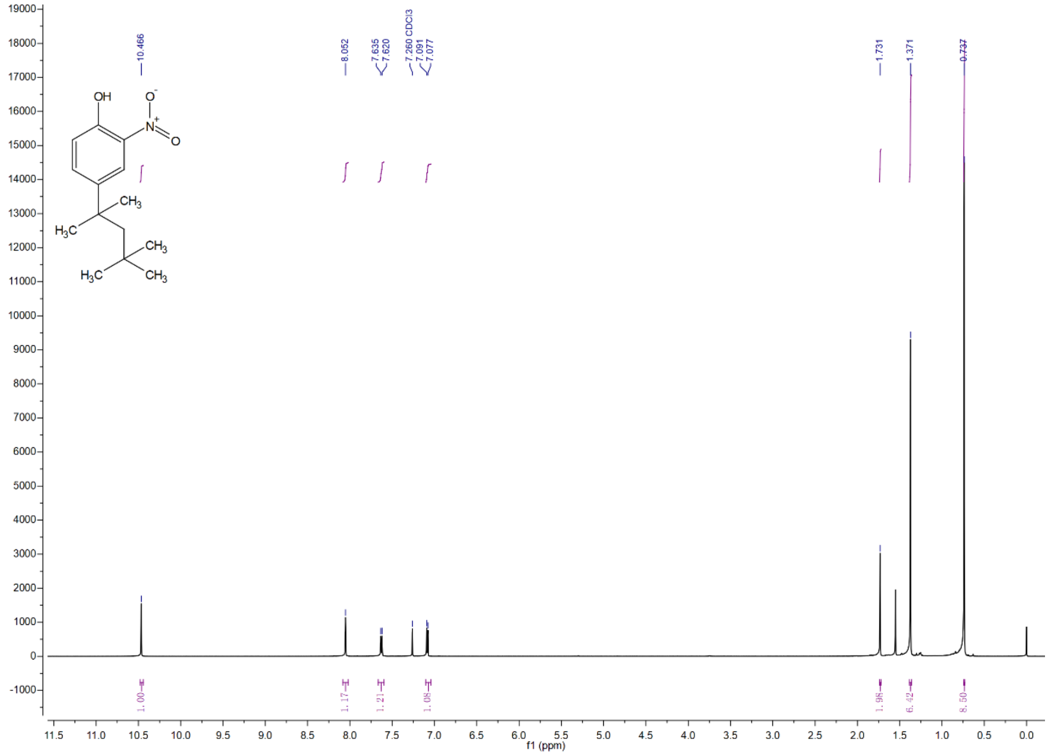

^{1}H-NMR spectrum copies of compound **1**

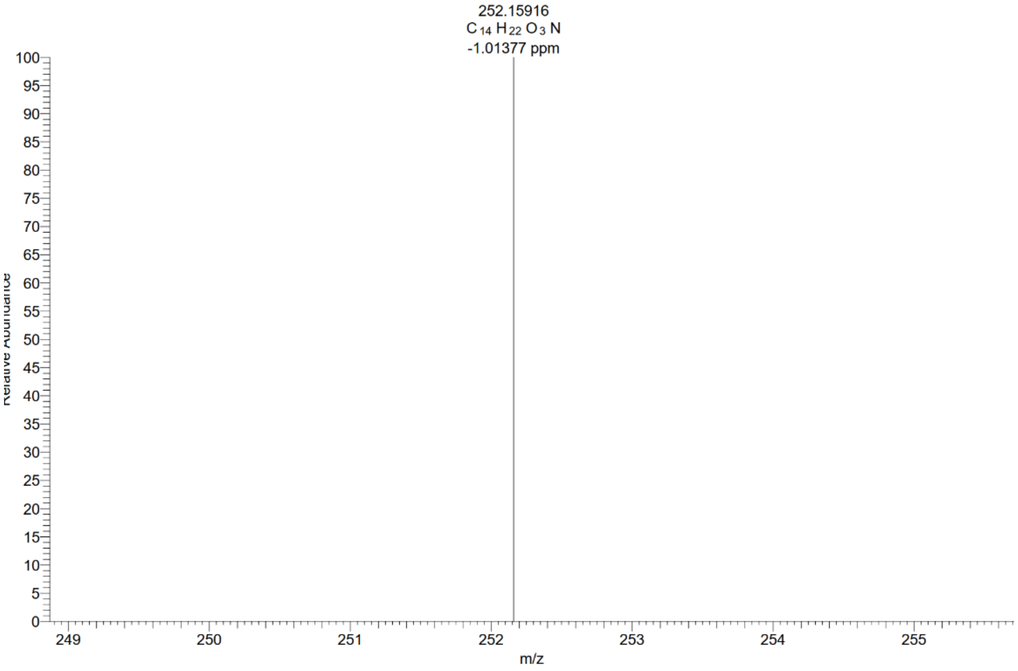

MS data of compound **1**

2. ^{1}H-NMR spectra and MS data of compound **2**

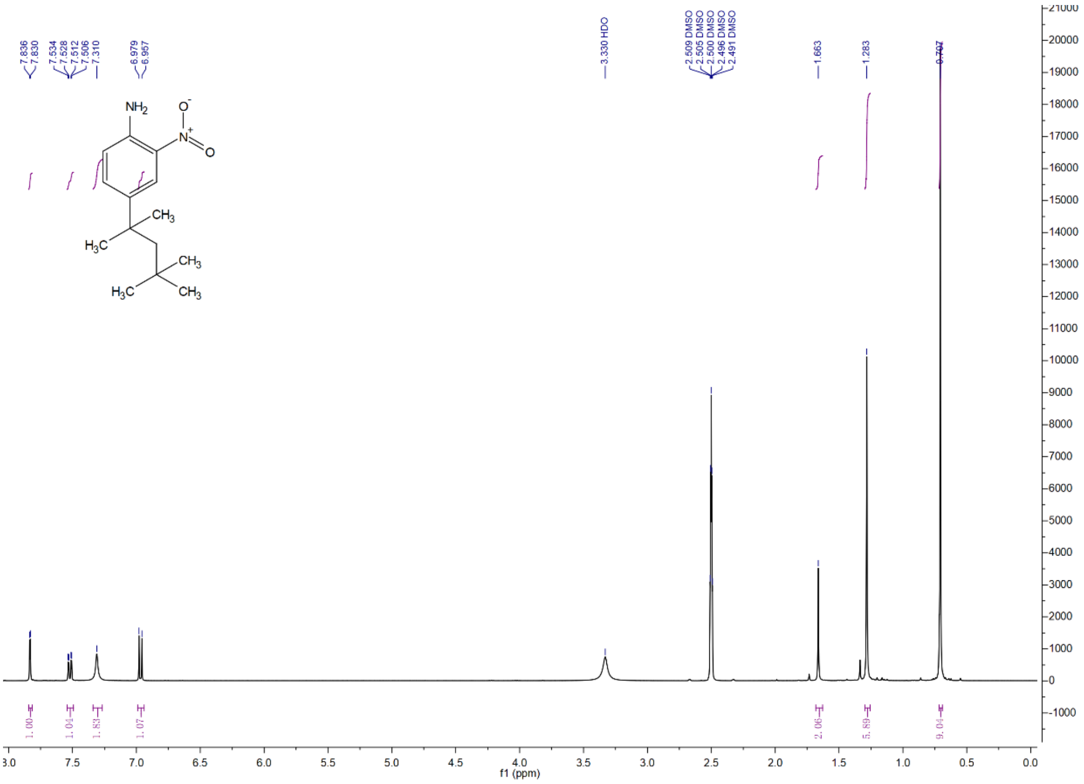

^{1}H-NMR spectrum copies of compound **2**

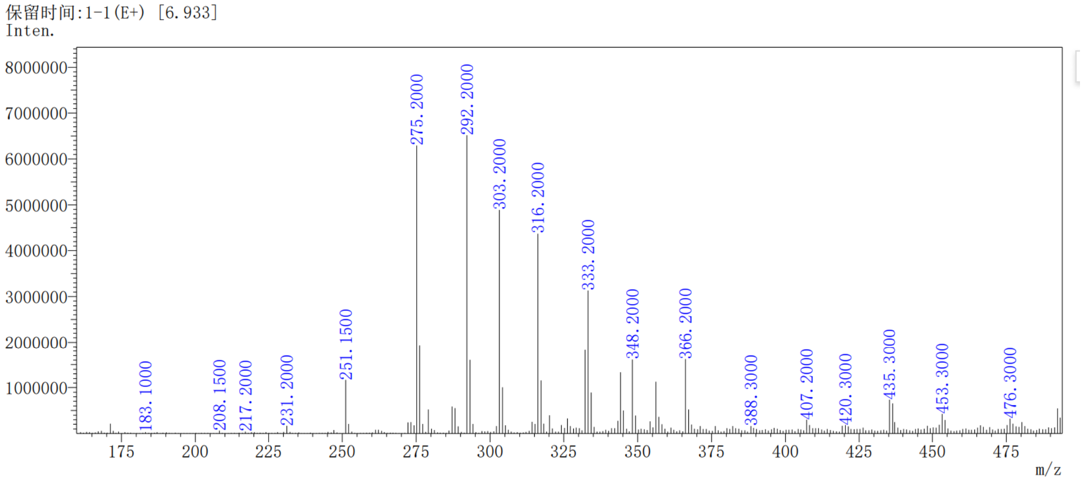

MS data of compound **2**

3. ^{1}H-NMR spectra and MS data of compound **3**

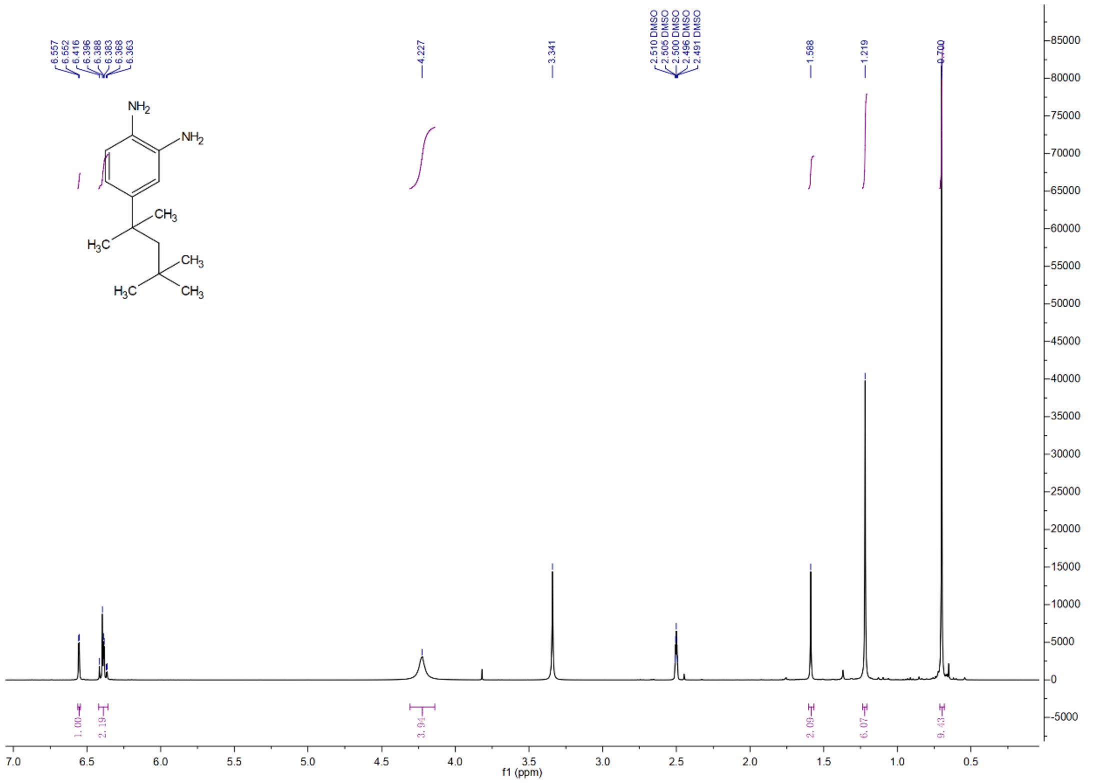

^{1}H-NMR spectrum copies of compound **3**

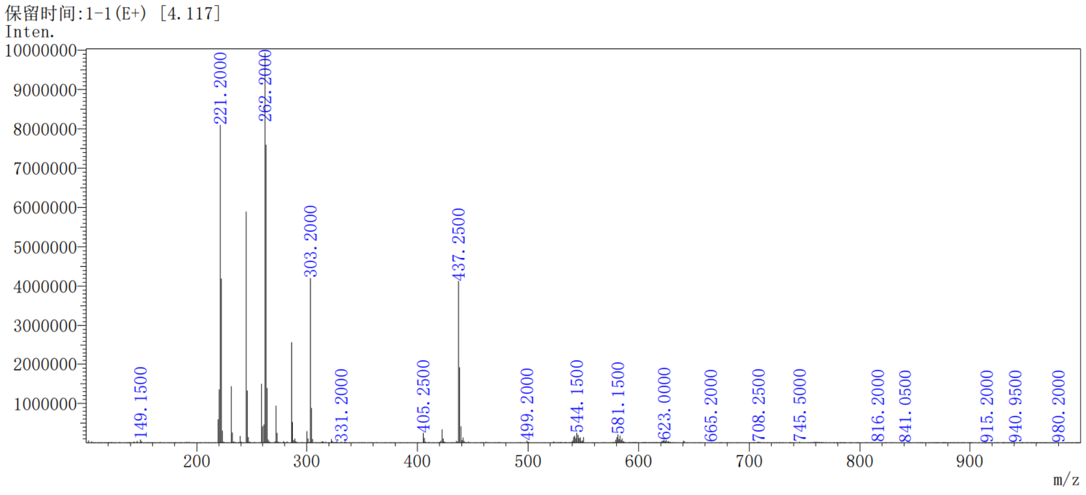

MS data of compound **3**

4. ^{1}H- and ^{3}C-NMR spectra, HRMS, and HPLC data of compound **N-8**

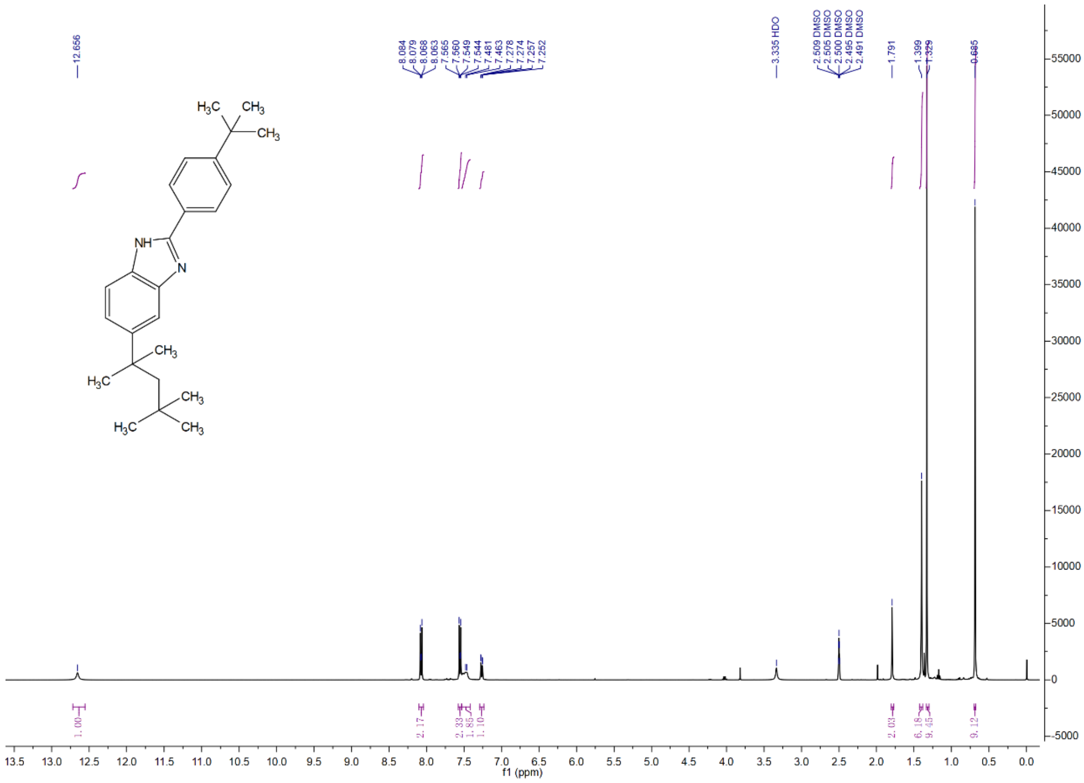

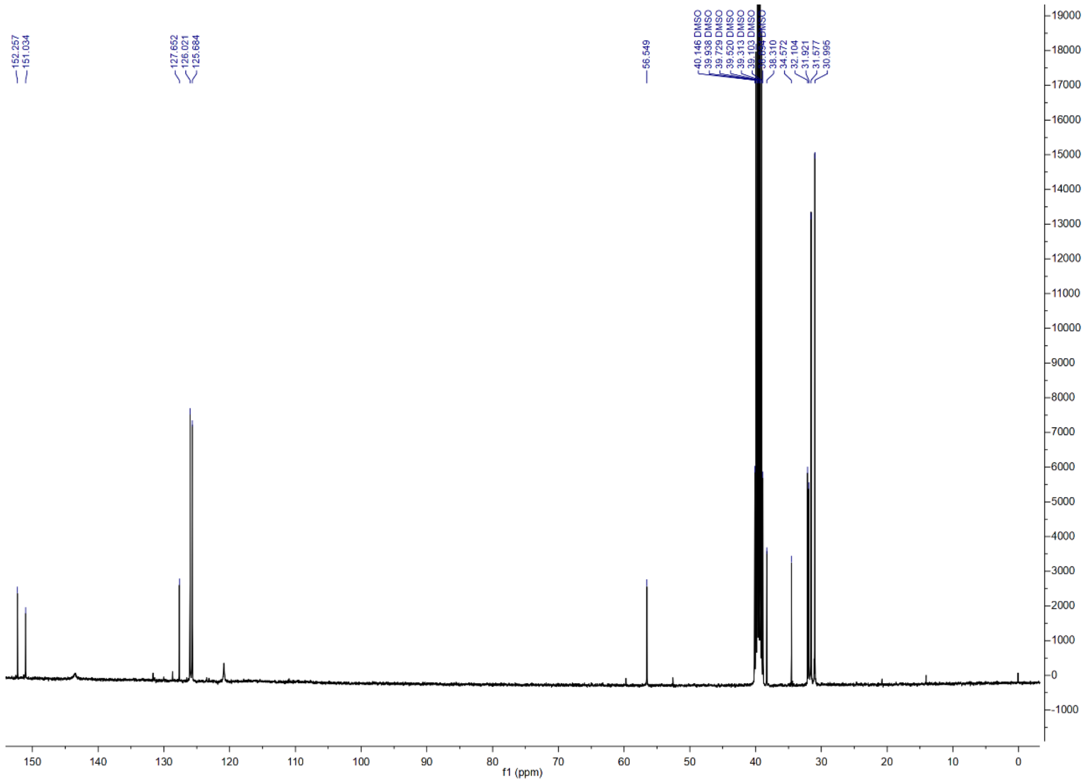

^{1}H- and ^{13}C-NMR spectrum copies of compound **N-8**

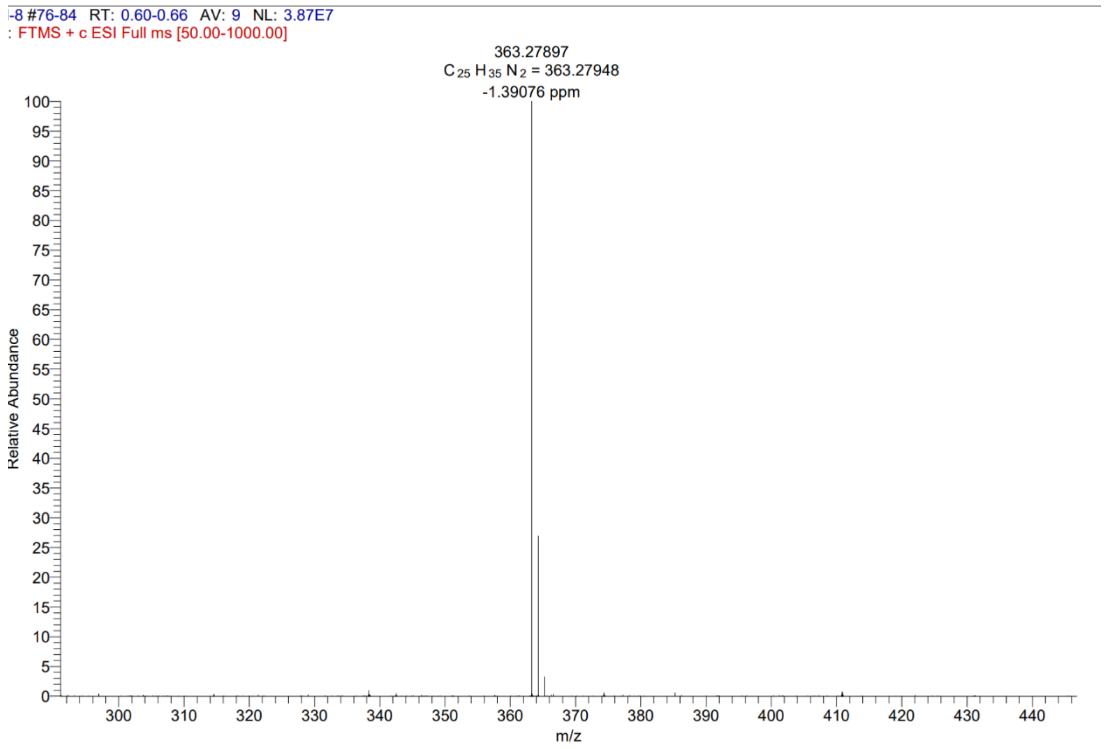

HRMS data of compound **N-8**

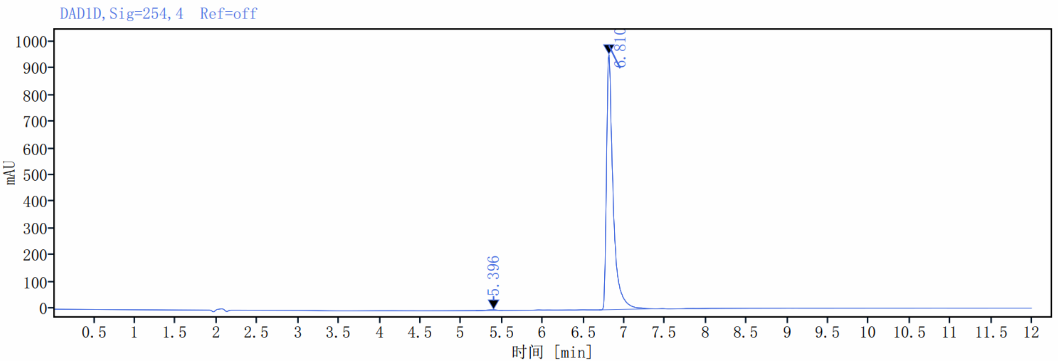

Signal: DAD1D, Sig=254, 4 Ref=off

Time [min]	Type	Width [min]	Area	Height	Area%
5.396	BM m	0.11	17.96	2.23	0.33
6.810	BM m	0.08	5409.45	953.68	99.67
		Total	5427.41		

HPLC data of compound **N-8**

5. The structures of reagents 2-chloroacetamide

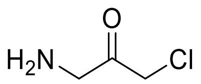

6. The structures of reagents 4-(tert-butyl)benzaldehyde

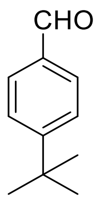
CHO

Supplementary Table 1. Clinical and molecular characteristics of the AML patient

**Category**	**Details**
Diagnosis	Acute Myeloid Leukemia (non-APL), M5b subtype
Cytogenetics	46, XX, inv(9)(p11q13)c[20]
Fusion genes	Negative
Key mutations (VAF%)	-*BCOR* c.510delG p.R170fs (40.0%)<br>-*DNMT3A* c.G2645A p.R882H (39.7%)<br>-*NPM1* c.859_860insTCTG p.L287fs (34.2%)<br>-*NRAS* c.A182C p.Q61P (29.3%)<br>-*FLT3* c.A2027C p.N676T (4.8%)
Treatment status	Relapsed after 2 months of mitoxantrone, cytarabine, venetoclax therapy

Supplementary Table 2. shRNA sequences

Name		Sequences (5’-3’)
CTSD-shRNA	Oligomer 1	GCTGCACAAGTTCACGTCCAT
	Oligomer 2	ACCTCGTTTGACATCCACTAT
	Oligomer 3	CATCACCTTCATCGCAGCCAA
TRIM21-shRNA	Oligomer 1	GGAAGTCACTTCACCATCA
	Oligomer 2	CCTGAAGGACCTGGATATTAC
	Oligomer 3	GAAGAGAGATTTGATAGTTAT

Supplementary Table 3. Quantitative real-time PCR primer sequences

Name		Sequences (5'-3')
human *BCL2*	F	ATCGCCCTGTGGATGACTGAGT
	R	GCCAGGAGAAATCAAACAGAGGC
human *BCL-XL*	F	GCCACTTACCTGAATGACCACC
	R	AACCAGCGGTTGAAGCGTTCCT
human *MCL1*	F	CCAAGAAAGCTGCATCGAACCAT
	R	CAGCACATTCCTGATGCCACCT
human *FBXW7*	F	GTTTGGTCAGCAGTCACAGGCA
	R	CCACACTTTGAGTGTCCGATCTG
human *ITCH*	F	AGCGTAGTCAGCTTCAAGGAGC
	R	AGGTGGCAATGGACCAAGAGGA
human *TRIM21*	F	CAGAACTCAGGAGTGTGTGCCA
	R	TCCAAGCCTCACTTGTCTCCGA
Human *PELI1*	F	AACCAGATCGGCTCAGCAGAGA
	R	ATGCTTCACGGTAGGAGTGTGG
human *GAPDH*	F	GTCTCCTCTGACTTCAACAGCG
	R	ACCACCCTGTTGCTGTAGCCAA
human *ACTB*	F	CACCATTGGCAATGAGCGGTTC
	R	AGGTCTTTGCGGATGTCCACGT

Supplementary methods

Quantitative proteomic analysis

Each sample was lysed in an appropriate volume of SDT lysis buffer (4% SDS, 100 mM Tris-HCl, pH 7.6) for protein extraction. Proteins were digested using the Filter-Aided Sample Preparation (FASP) method with trypsin. The resulting peptides were analyzed using an Orbitrap™ Astral™ mass spectrometer (Thermo Scientific) coupled to a Vanquish Neo UHPLC system (Thermo Scientific) in data-independent acquisition (DIA) mode. DIA data were processed using DIA-NN (version 1.8.1). Protein identification was performed at 99% confidence, with a false discovery rate (FDR) ≤ 1%. Differentially expressed proteins were selected based on a fold change >1.5 and p <0.05, as measured by Student's t-test.

Chemistry

All reagents were obtained from Bide Pharmatech Ltd. (Shanghai, China) and used without further purification. Reactions were monitored by thin layer chromatography using aluminum TLC plates (60F254D, Merck Millipore, Billerica, Massachusetts, USA). Compounds were isolated and purified using a Combiflash Rf+ system (Teledyne Isco, Lincoln, Nebraska, USA) and a Slica Flash Column (Santai Technologies, Xi'an, China). LC-MS analysis was conducted with a Shimadzu LC-MS 2020 system equipped with an electrospray ionization (ESI) source and a single-quadrupole mass analyzer. A Shim-pack VP-ODS column (2.0 mm × 150 mm, 5 μm) was employed, with a solvent

B gradient of 10-90% at a flow rate of 0.5 mL/min (solvent A: 0.1% formic acid in water; solvent B: 0.1% formic acid in acetonitrile). MS spectra were obtained in negative ion mode over a scan range of m/z 100−800. High-resolution mass spectrometry (HRMS) was performed using an LTQ Orbitrap XL (Thermo Scientific, Waltham, Massachusetts, USA). HPLC analysis was carried out with an Agilent 1260 system using an XDB-C18 column (4.6 mm × 150 mm, 5 μm) or an Agilent 1200 system with an EC-C18 column (4.6 mm × 150 mm, 4 μm), utilizing acetonitrile and water as mobile phases at a flow rate of 0.8 mL/min. Analytical HPLC indicated that the purity of all target compounds was greater than 95%. ^{1}H NMR and ^{13}C NMR spectra were recorded in $CDCl_3$ or DMSO-d_6 (Cambridge Isotope Laboratories, Andover, Massachusetts USA) using a Bruker 400 MHz or Bruker 600 MHz spectrometer (Bruker Bioscience, Billerica, Massachusetts, USA).

The target compound **N-8** was synthesised based on the reactions outlined in Scheme 1. Compound **1** was obtained in 85% yield by the nitration of a commercially available compound for 30 min. Compound **1** and 2-chloroacetamide were reacted cross Smiles rearrangement to obtain compound **2**. The synthesis of the key intermediate **3** was achieved in 87% yield by reducing the nitro-compound **2** in the presence of 10% Pd/C and $HCOONH_4$. The reaction of compound **3** and 4-(tert-butyl)benzaldehyde obtained the target compound **N-8** under the conditions of 30% H_2O_2 and $(NH_4)_2Ce(NO_3)_6$.

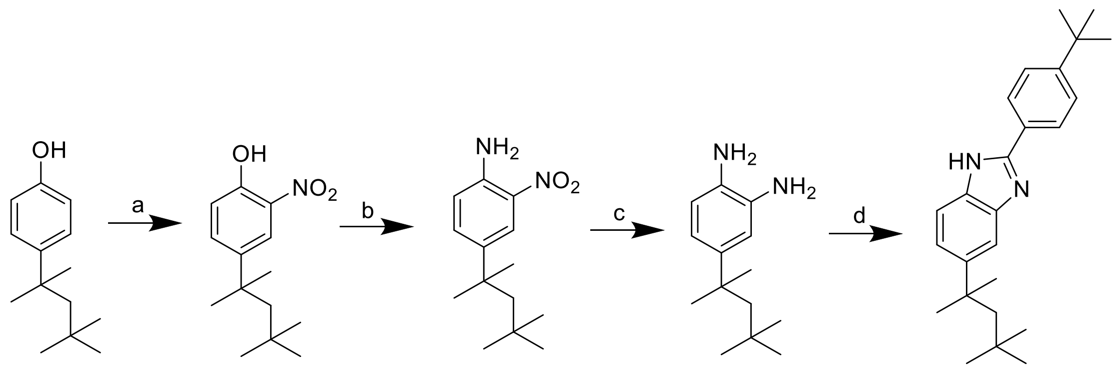

1 2 3 **N-8**

Scheme 1. Synthetic route for target compounds. Reagents and conditions: (a) HNO_3, CH_3COOH, room temperature, 30 min; (b) 2-chloroacetamide, Cs_2CO_3, DMF, 100°C, 14 h; (c) $HCOONH_4$, Pd/C, MeOH, room temperature, 2 h; (d) 30% H_2O_2, $(NH_4)_2Ce(NO_3)_6$, 4-(tert-butyl)benzaldehyde, CH_3CN, 50°C, 2 h.

2-nitro-4-(2,4,4-trimethylpentan-2-yl) phenol (**1**). 4-(2,4,4-trimethylpentan-2-yl) phenol (2.06 g, 10 mmol) was dissolved in acetic acid (20 mL), and concentrated nitric acid (1 mL) was added drop by drop and stirred for 30 min at room temperature. It was distilled under reduced pressure, distilled water (30 mL) was added, and extracted with ethyl acetate (25 mL × 3). The merged organic layer was dried with anhydrous Na_2SO_4, concentrated under reduced pressure, and purified by silica gel to obtain an orange oil (2.13 g, 85% yield). ^1H NMR (600 MHz, Chloroform-*d*) δ 10.47 (s, 1H), 8.05 (s, 1H), 7.63 (d, J = 8.8 Hz, 1H), 7.08 (d, J = 8.9 Hz, 1H), 1.73 (s, 2H), 1.37 (s, 6H), 0.74 (s, 9H).MS (ESI) m/z: 252 $[M + H]^+$.

2-nitro-4-(2,4,4-trimethylpentan-2-yl)aniline (**2**). The compound **1** (1255 mg, 5 mmol) and 2-chloroacetamide (561 mg, 6 mmol) were dissolved in DMF (100 ml), and Cs_2CO_3

(16.3 g, 50 mmol) was added, then the mixture was raised to 100°C for 14 h. It was distilled under reduced pressure, distilled water (50 mL) was added, and extracted with ethyl acetate (40 mL × 3). The merged organic layer was dried with anhydrous Na_2SO_4, concentrated under reduced pressure, and purified by silica gel to obtain a brown solid (787.5 mg, 63%). Mp: 95.3-96.7°C. ^{1}H NMR (400 MHz, DMSO-d_6) δ 7.83 (d, J = 2.3 Hz, 1H), 7.52 (dd, J = 8.9, 2.4 Hz, 1H), 7.31 (s, 2H), 6.97 (d, J = 8.9 Hz, 1H), 1.66 (s, 2H), 1.28 (s, 6H), 0.71 (s, 9H). MS (ESI) m/z: 251 $[M + H]^+$.

4-(2,4,4-trimethylpentan-2-yl)benzene-1,2-diamine (**3**). Compound **2** (500 mg, 2 mmol) was dissolved in methanol (25 mL), ammonium formate (252 mg, 4 mmol) and 30% Pd/C (100 mg, 20% w/w) were added and stirred for 2 h at room temperature. Pd/C was filtered, distilled under reduced pressure, distilled water (30 mL) was added, and extracted with ethyl acetate (25 mL × 3). The consolidated organic layer was dried with anhydrous Na_2SO_4, concentrated under reduced pressure, and purified by column chromatography to obtain a brown solid (365.2 mg, 83% yield). Mp: 98.1-100. 5°C. ^{1}H NMR (400 MHz, DMSO-d_6) δ 6.55 (d, J = 1.9 Hz, 1H), 6.42 – 6.36 (m, 2H), 4.23 (s, 4H), 1.59 (s, 2H), 1.22 (s, 6H), 0.70 (s, 9H). MS (ESI) m/z: 221 $[M + H]^+$.

2-(4-(tert-butyl)phenyl)-5-(2,4,4-trimethylpentan-2-yl)-1H-benzo[d]imidazole (**N-8**). The compound **3** (220 mg, 1 mmol), 4-tert-butylbenzaldehyde (194.4 mg, 1.2 mmol) and $(NH_4)_2Ce(NO_3)_6$ (16.3 g, 50 mmol) were dissolved in CH_3CN (20 ml), and 30% H_2O_2 (0.4 mL, 4 mmol,) was added drop by drop at 0°C, and then the mixture was

raised to 50°C for 2 h. It was distilled under reduced pressure, distilled water (30 mL) was added, and extracted with ethyl acetate (25 mL × 3). The merged organic layer was dried with anhydrous Na_2SO_4, concentrated under reduced pressure, and purified by silica gel to obtain a light-yellow solid (787.5 mg, 63%). Mp:137.7-139.4°C. ^{1}H NMR (400 MHz, DMSO-d_6) δ 12.66 (s, 1H), 8.10 – 8.04 (m, 2H), 7.58 – 7.54 (m, 2H), 7.47 (d, *J* = 7.3 Hz, 2H), 7.27 (dd, *J* = 8.5, 1.8 Hz, 1H), 1.79 (s, 2H), 1.40 (s, 6H), 1.33 (s, 9H), 0.69 (s, 9H). ^{13}C NMR (101 MHz, DMSO) δ 152.26, 151.03, 127.65, 126.02, 125.68, 56.55, 38.31, 34.57, 32.10, 31.92, 31.58, 31.00. HRMS (ESI): calcd for $C_{25}H_{35}N_2$ $[M + H]^+$, 363.27948, found 363.27897.

Surface plasmon resonance (SPR) analysis

BIAcore T200 system (GE Healthcare) was used to analyze the surface plasmon resonance binding between CTSD and the indicated small molecules. In brief, CTSD protein (#RP00214, Abclonal, Wuhan, China) was immobilized onto channel 1 in a CM7 sensor chip (#28953828, Cytiva, Medford, USA) through a standard coupling protocol. To measure the binding kinetics, the indicated small molecule in twofold serial dilutions and a buffer blank for baseline subtraction were sequentially injected, with a regeneration step (glycine, pH 2.5) performed between each cycle. The equilibrium dissociation constant was calculated with Bia Evaluation Software 4.1.
