# Supplementary material for: Cathepsin D promotes acute myeloid leukemia progression through stabilization of the anti-apoptotic proteins
Source: Cell Death Dis. 2025 Aug 12;16(1):611. doi: 10.1038/s41419-025-07949-7 (PMC12343803; doi:10.1038/s41419-025-07949-7)

Original Western Blots

Figure 1


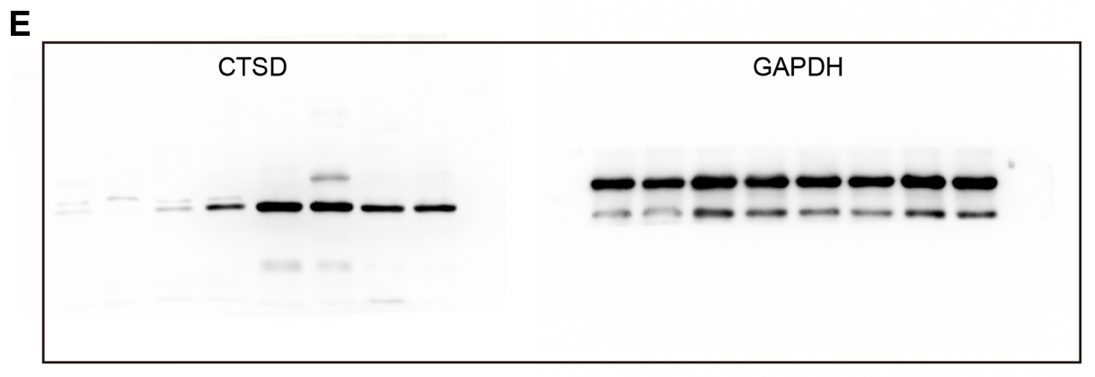


Figure 2


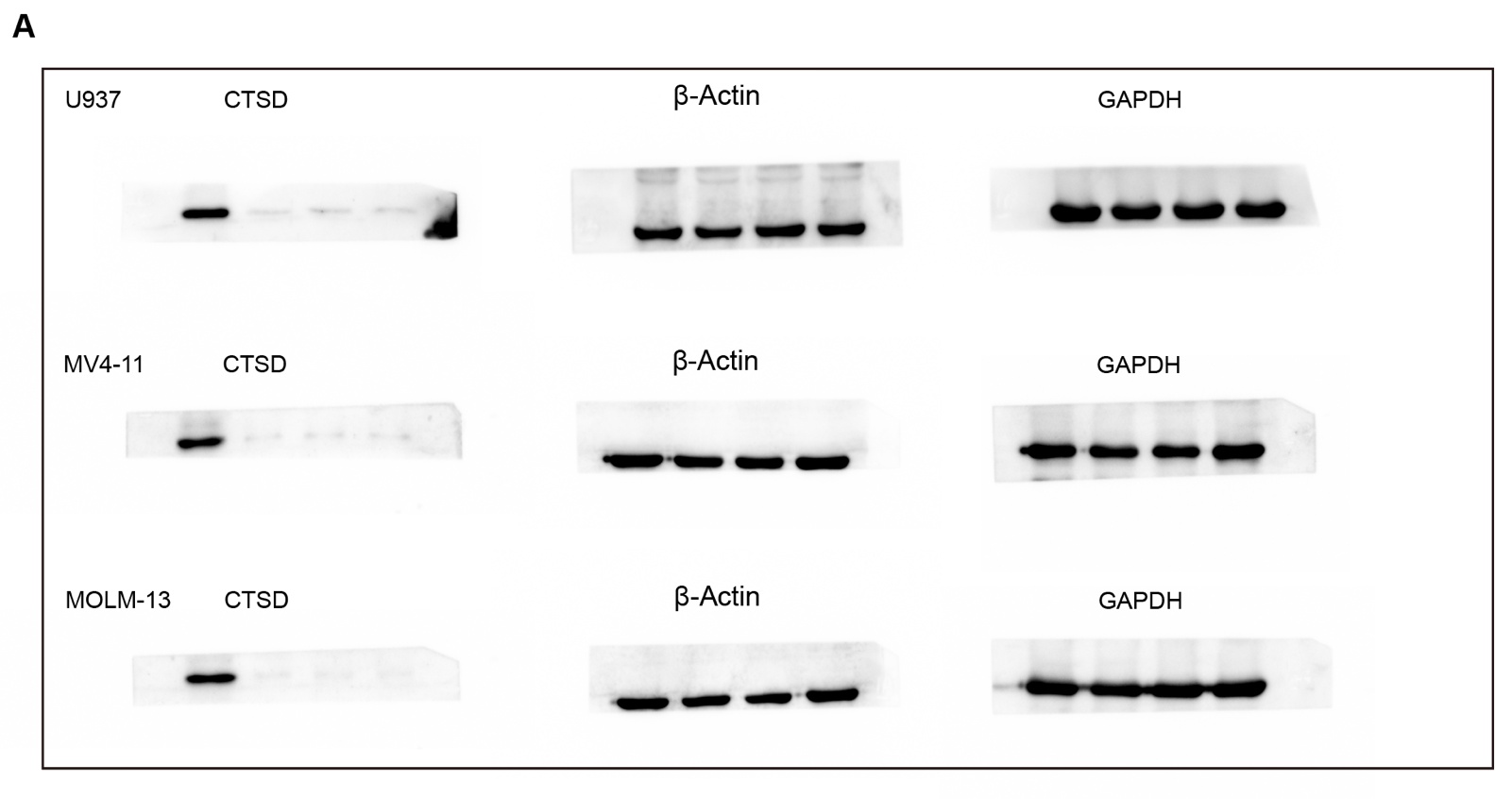


Figure 3


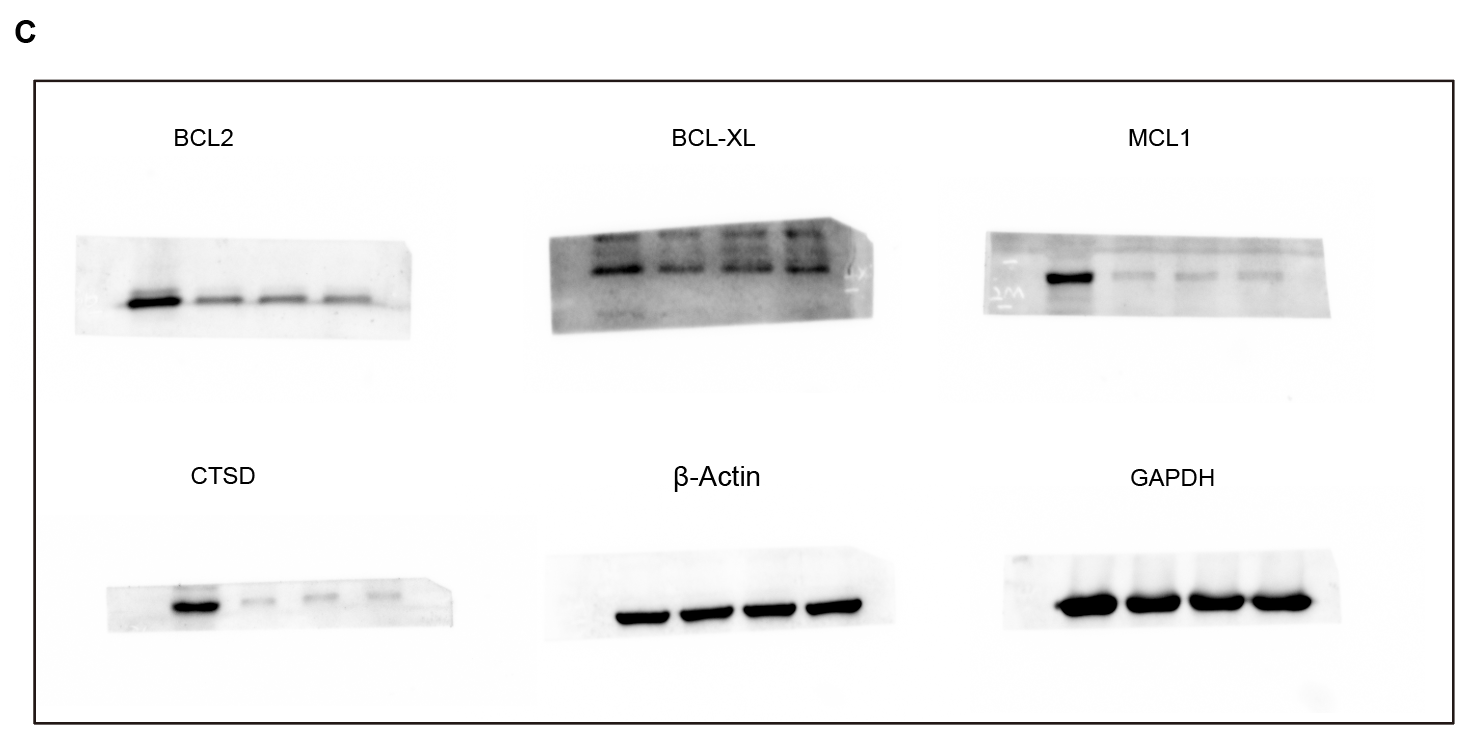


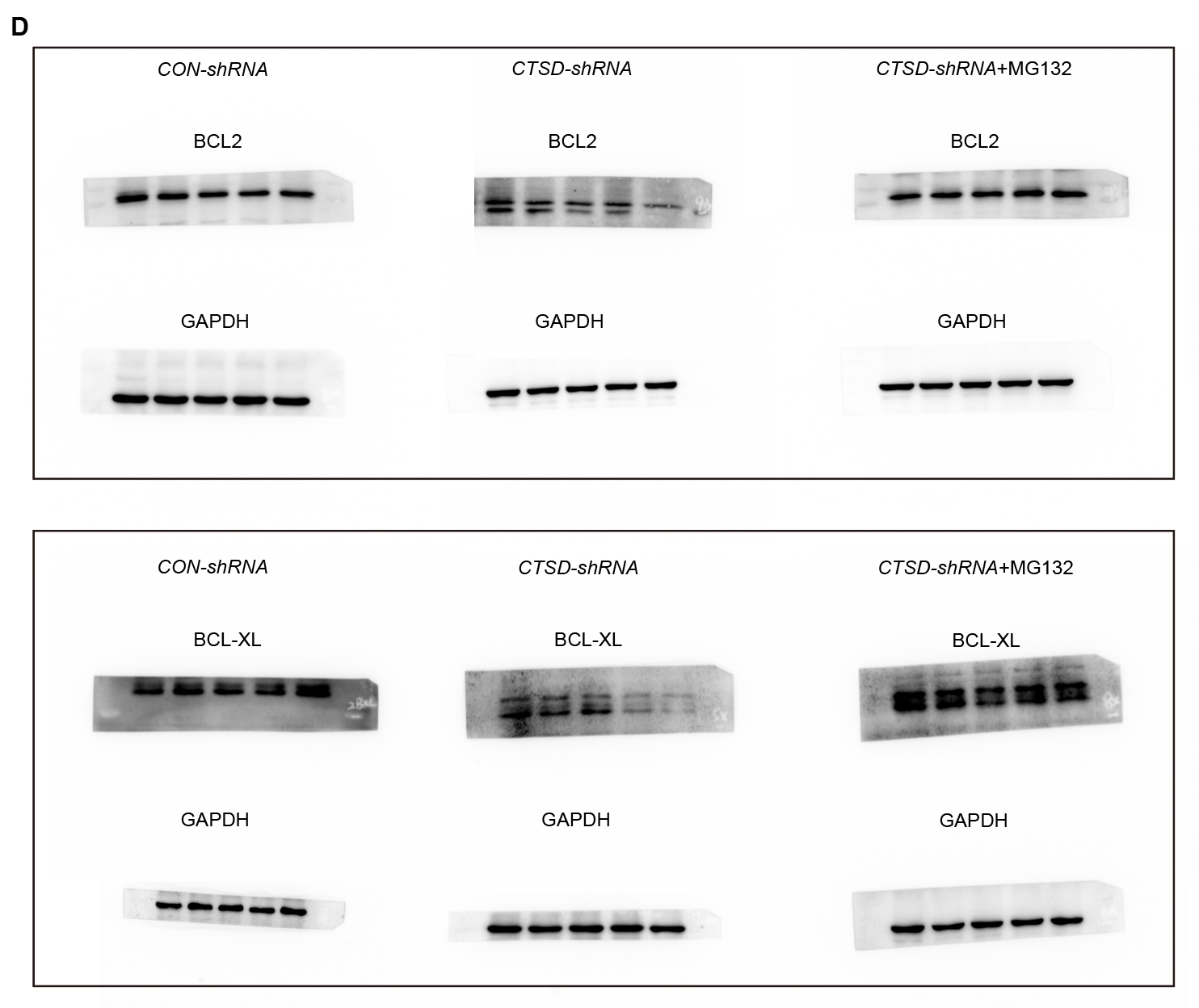


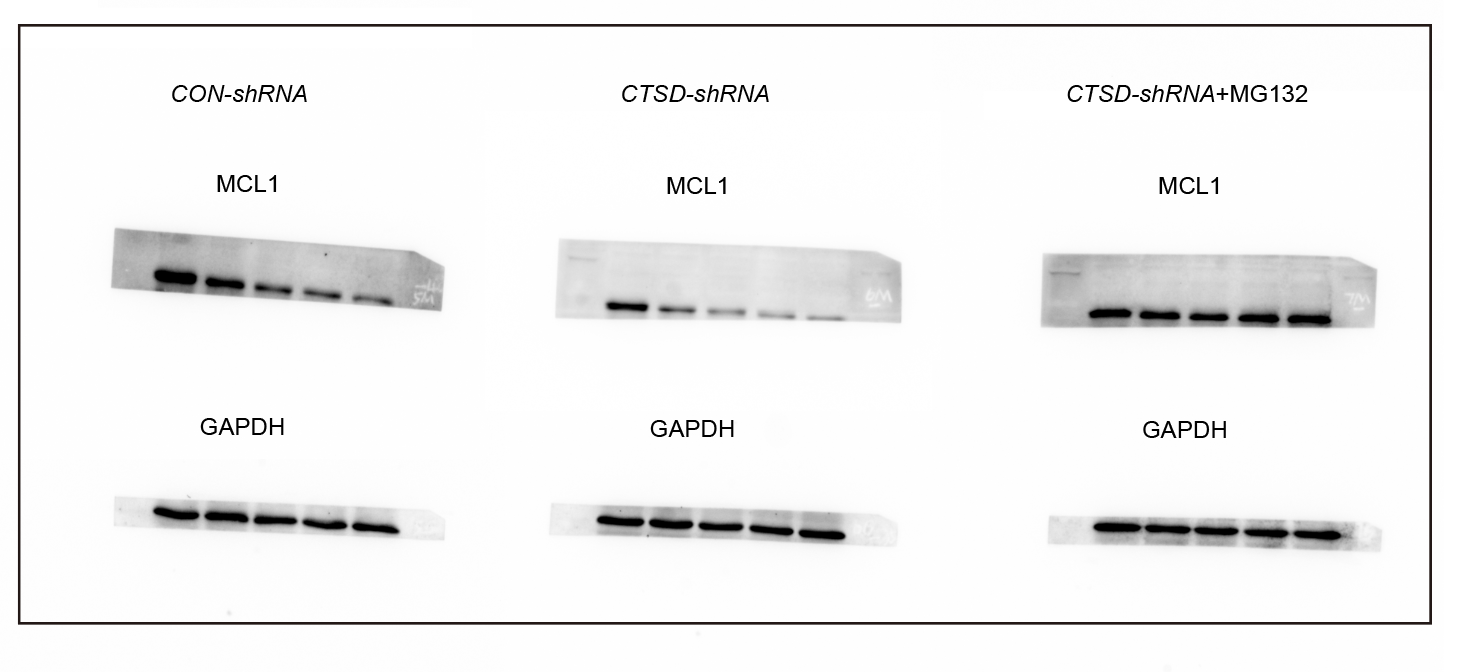


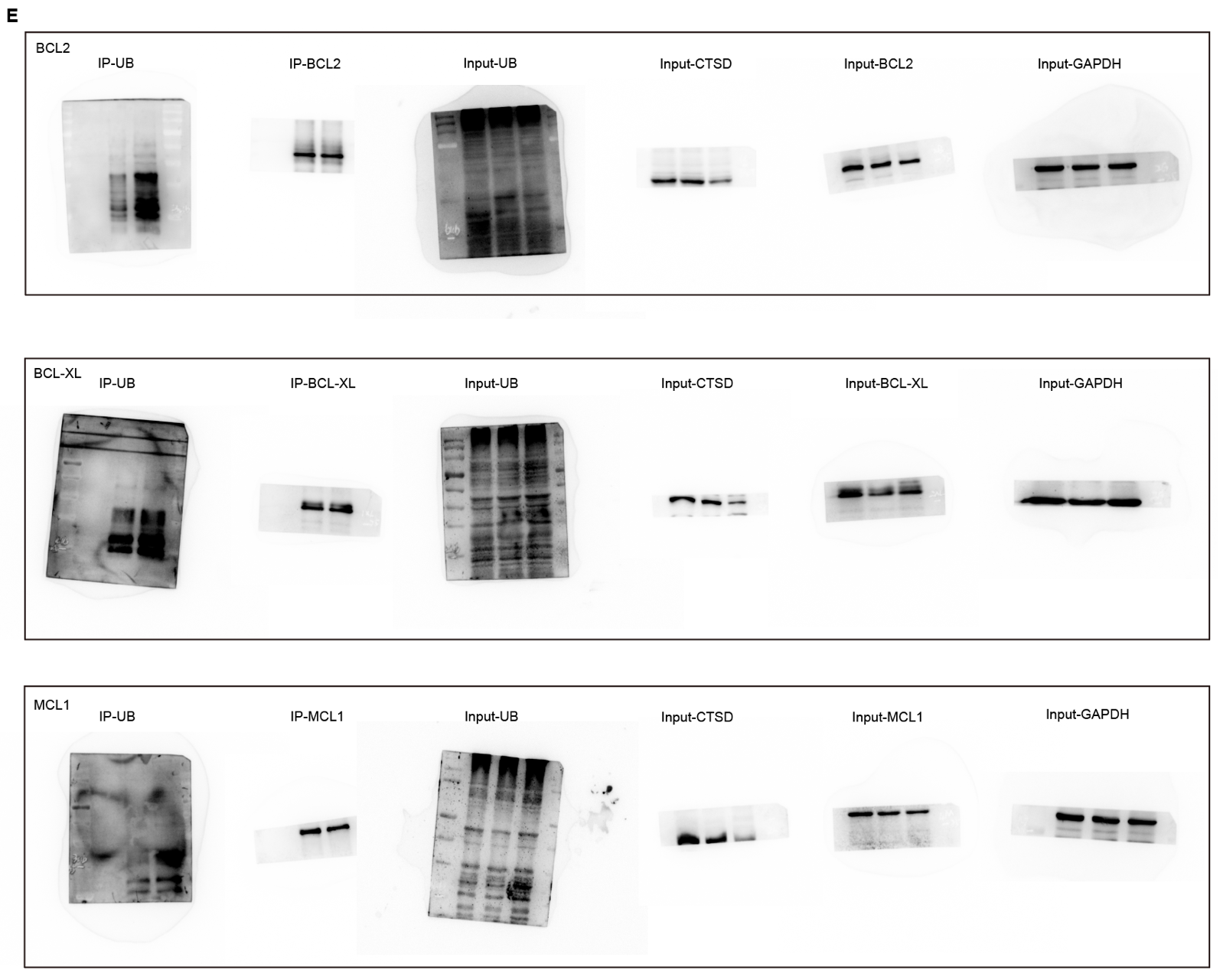


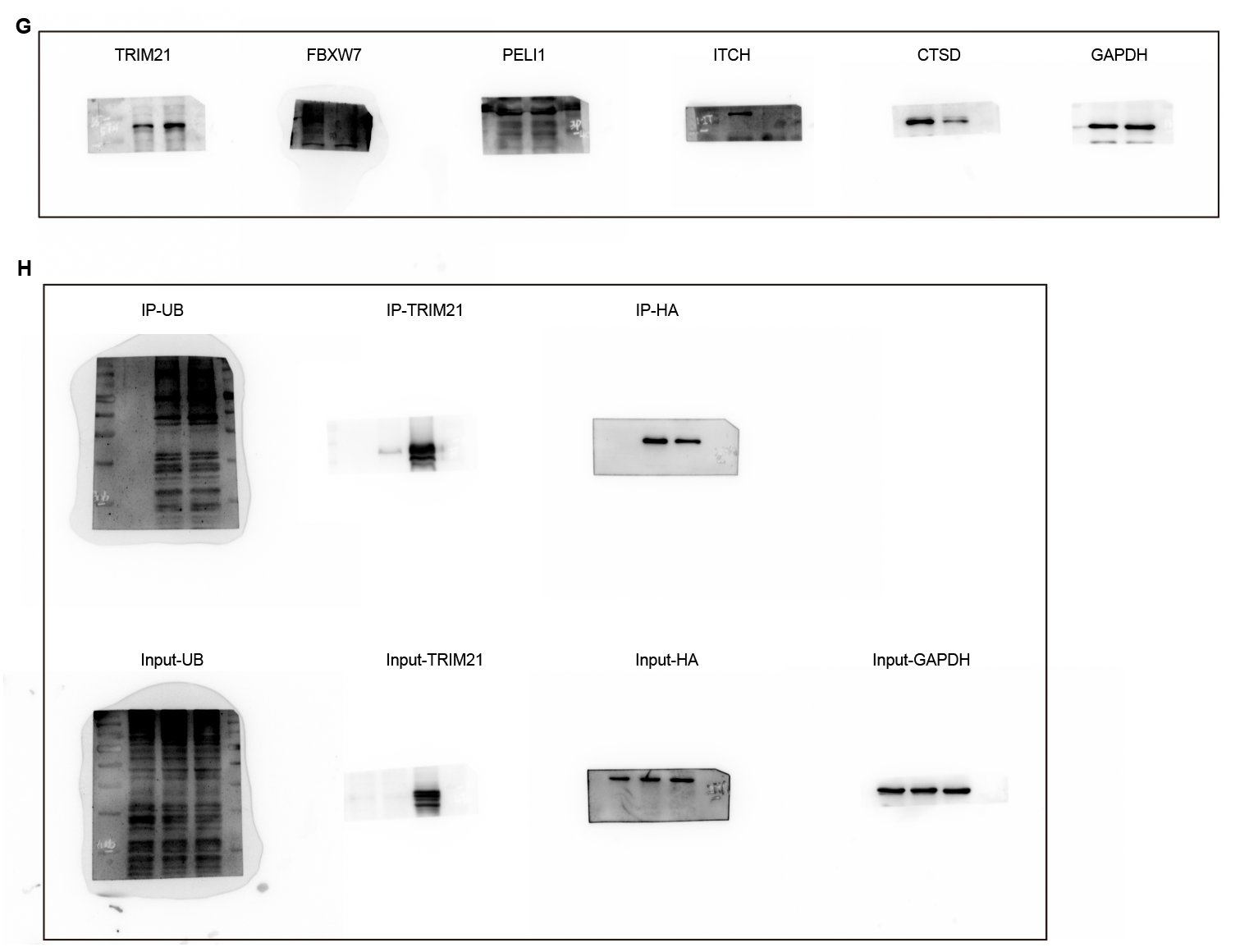


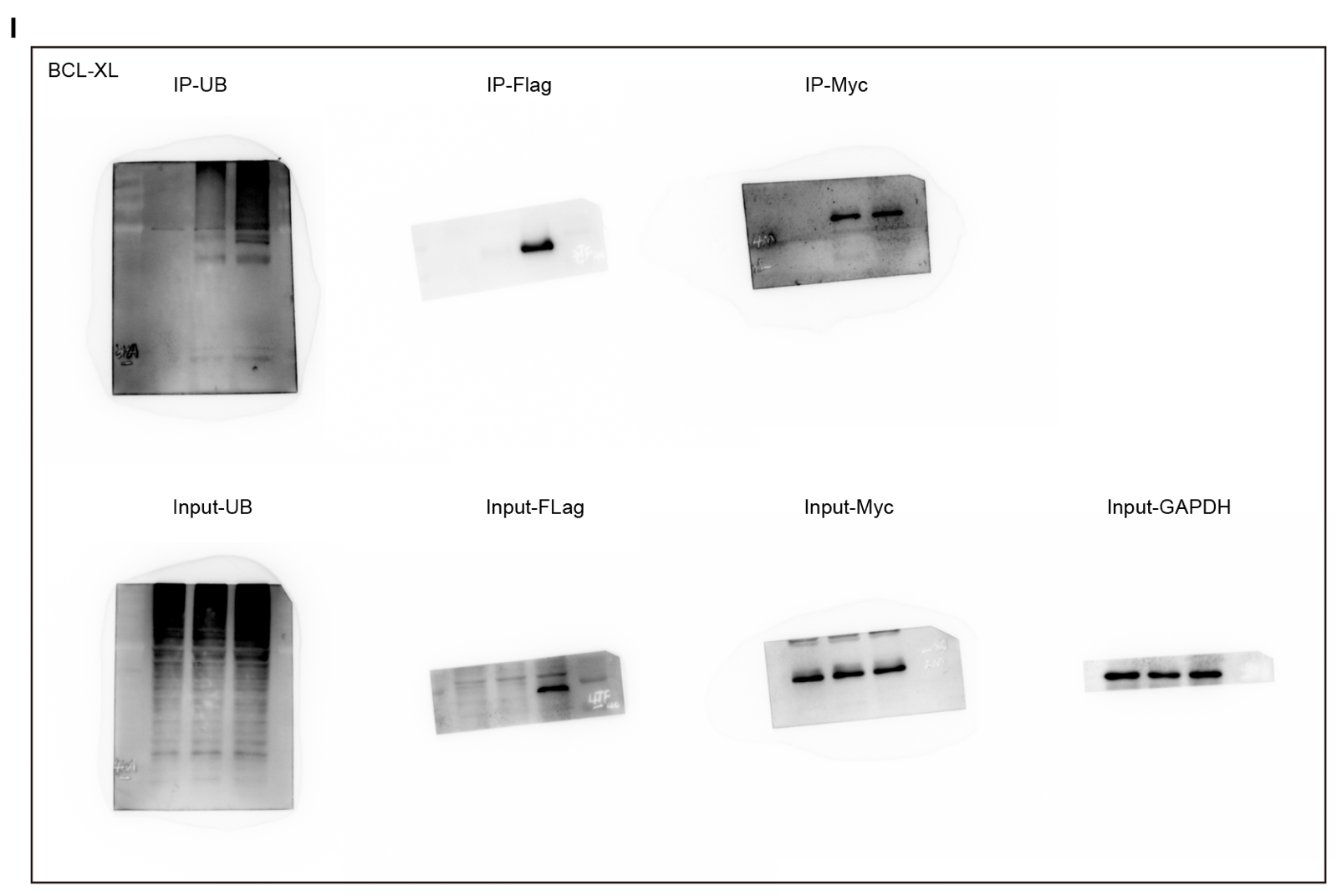


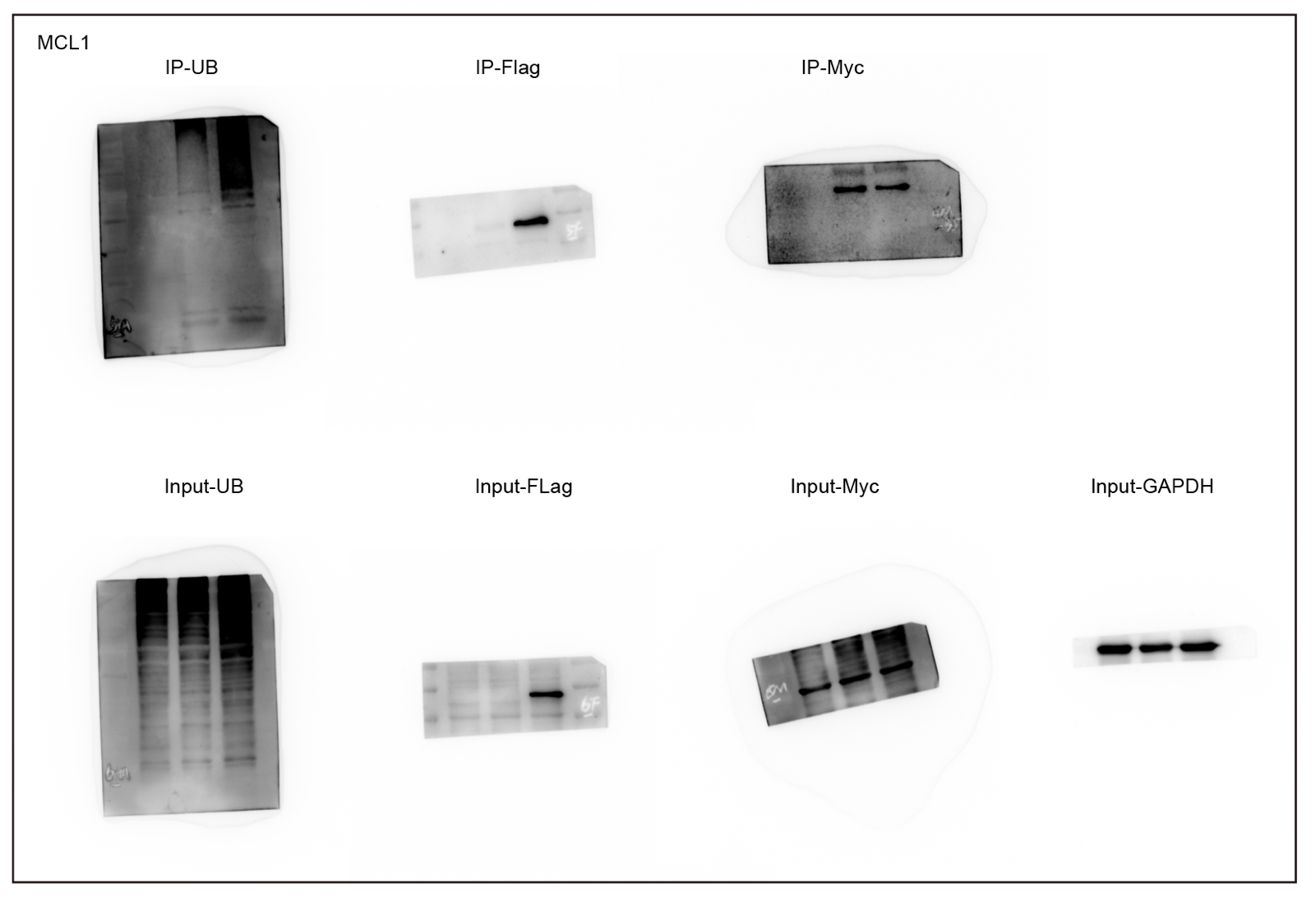


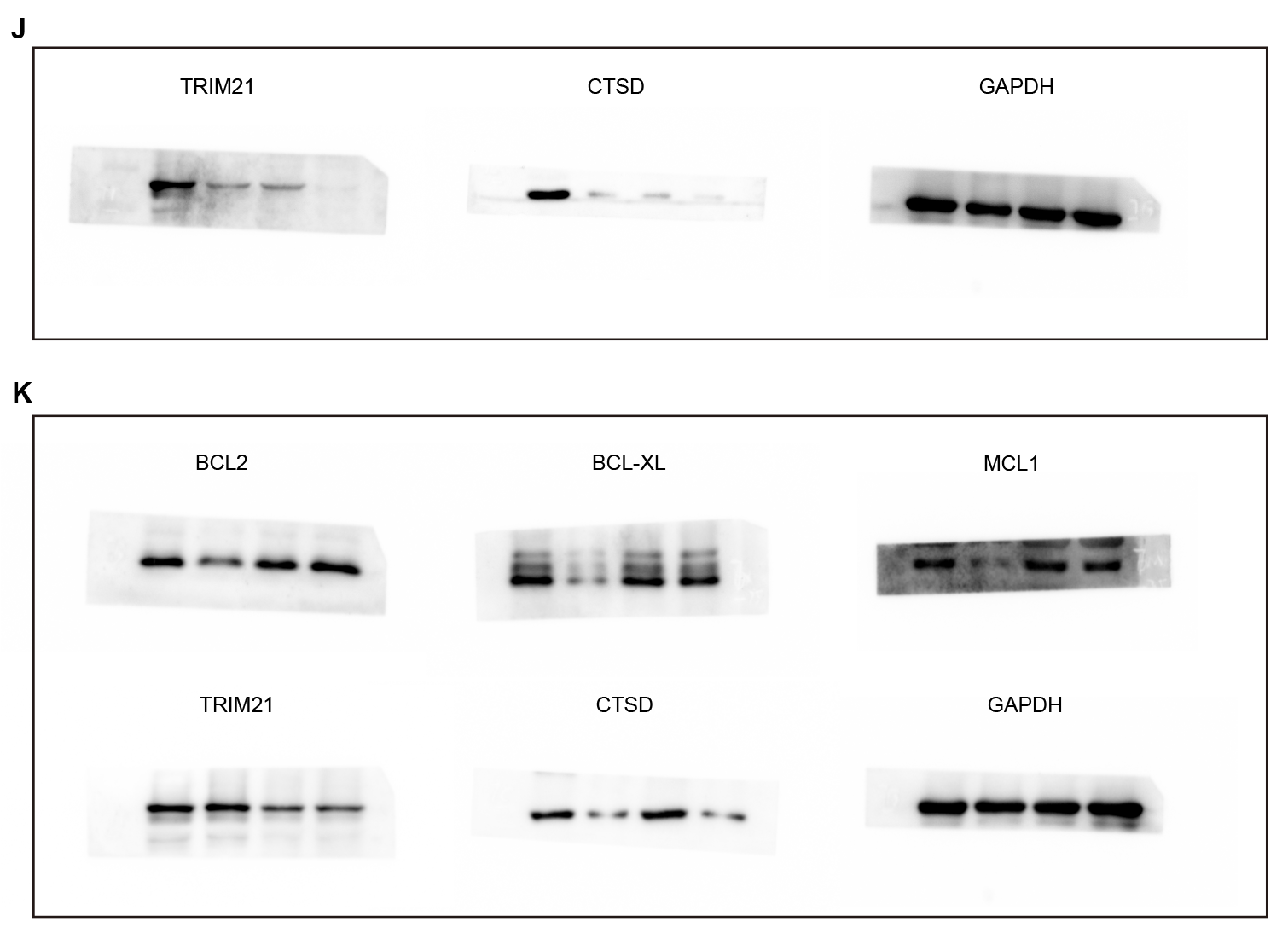


Figure 5


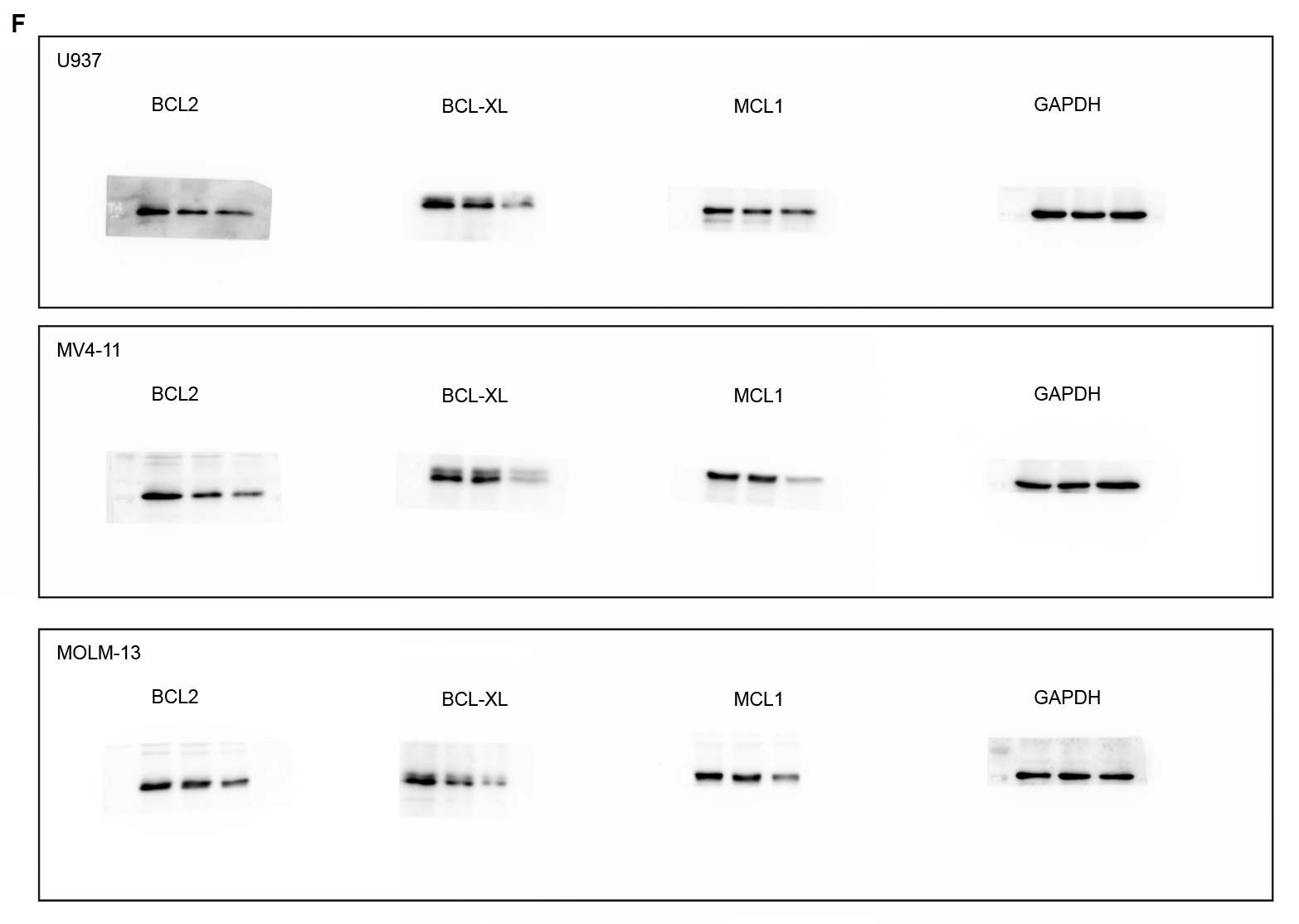


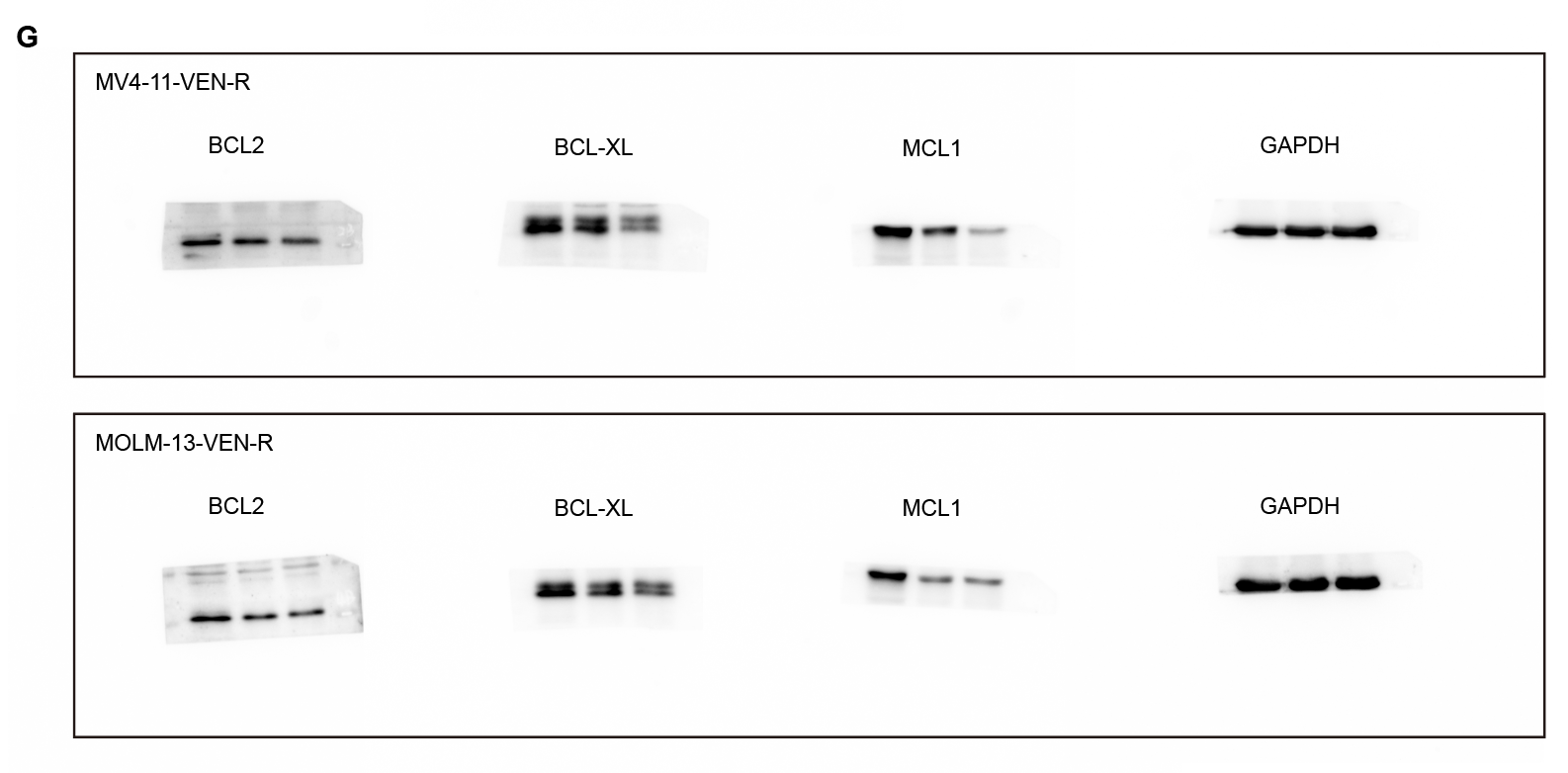


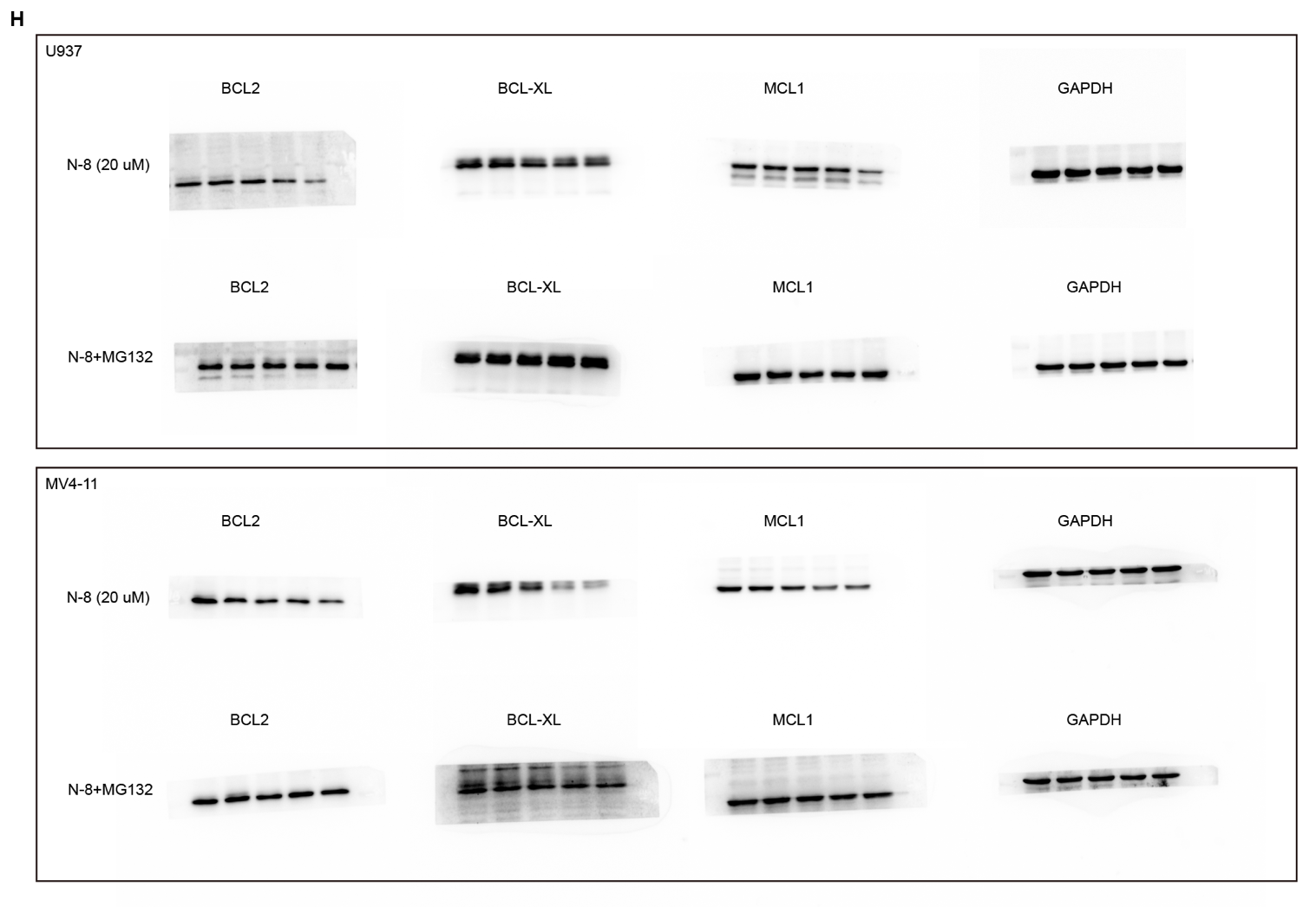


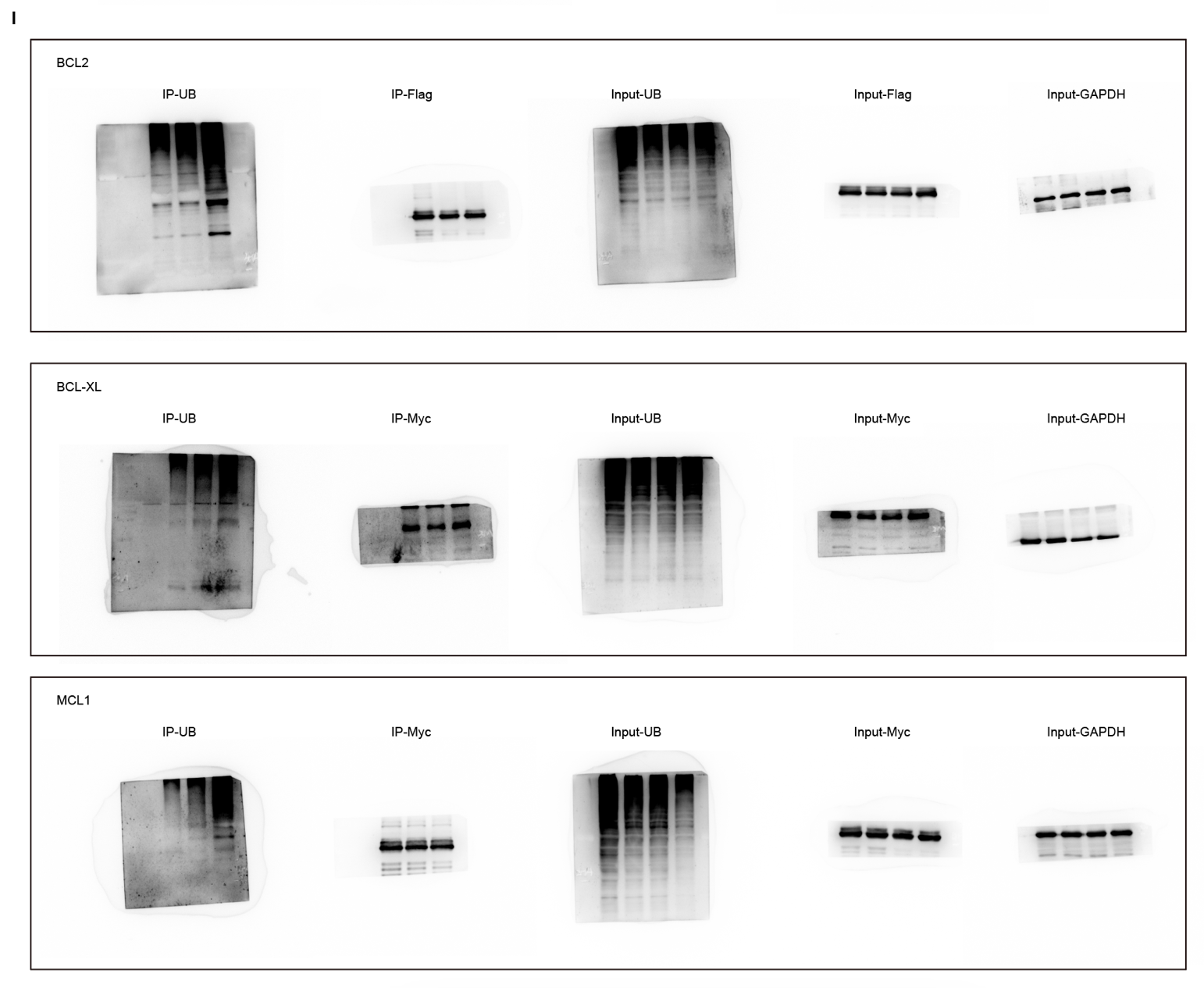


Figure 6


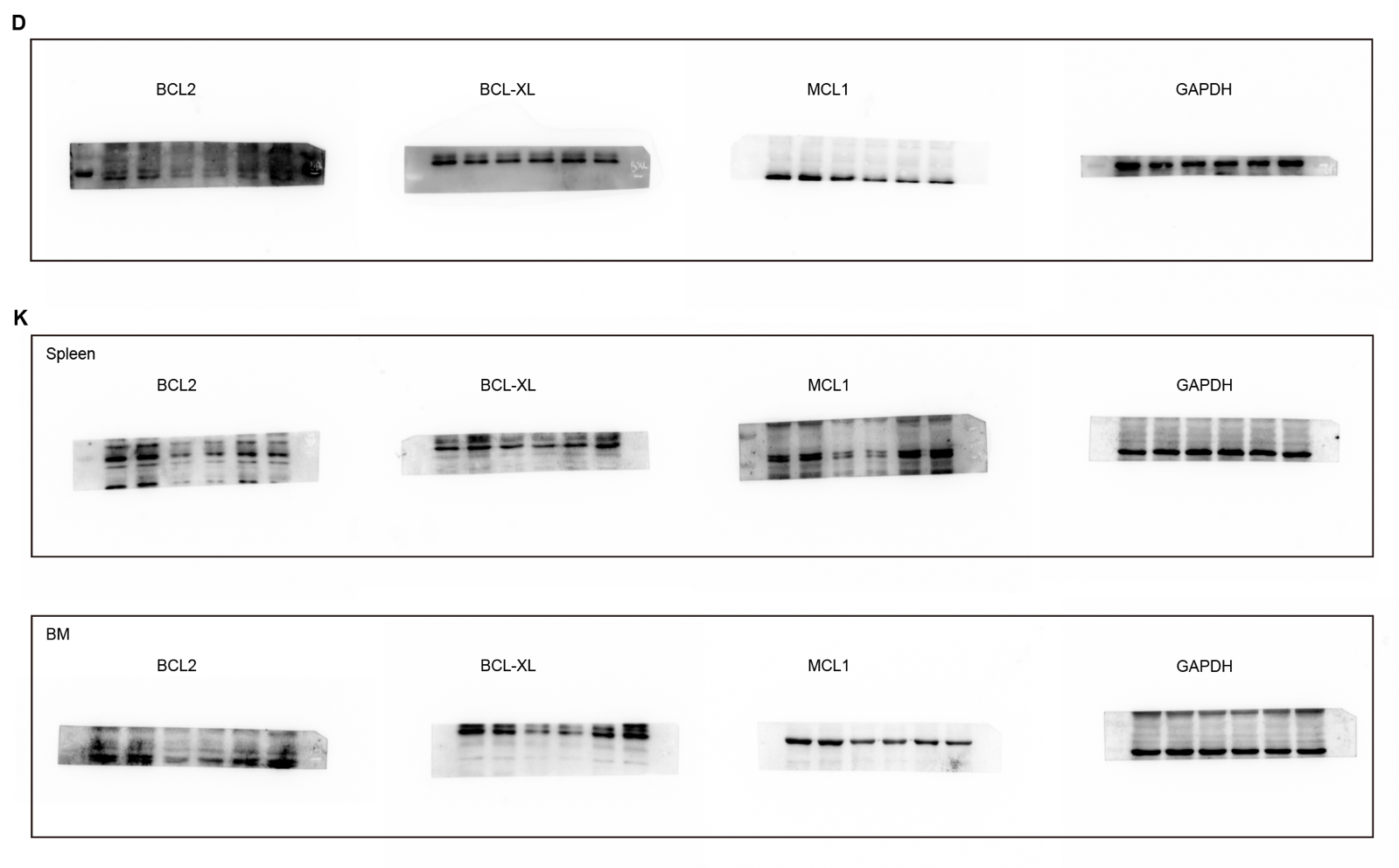


Supplementary Figure 2


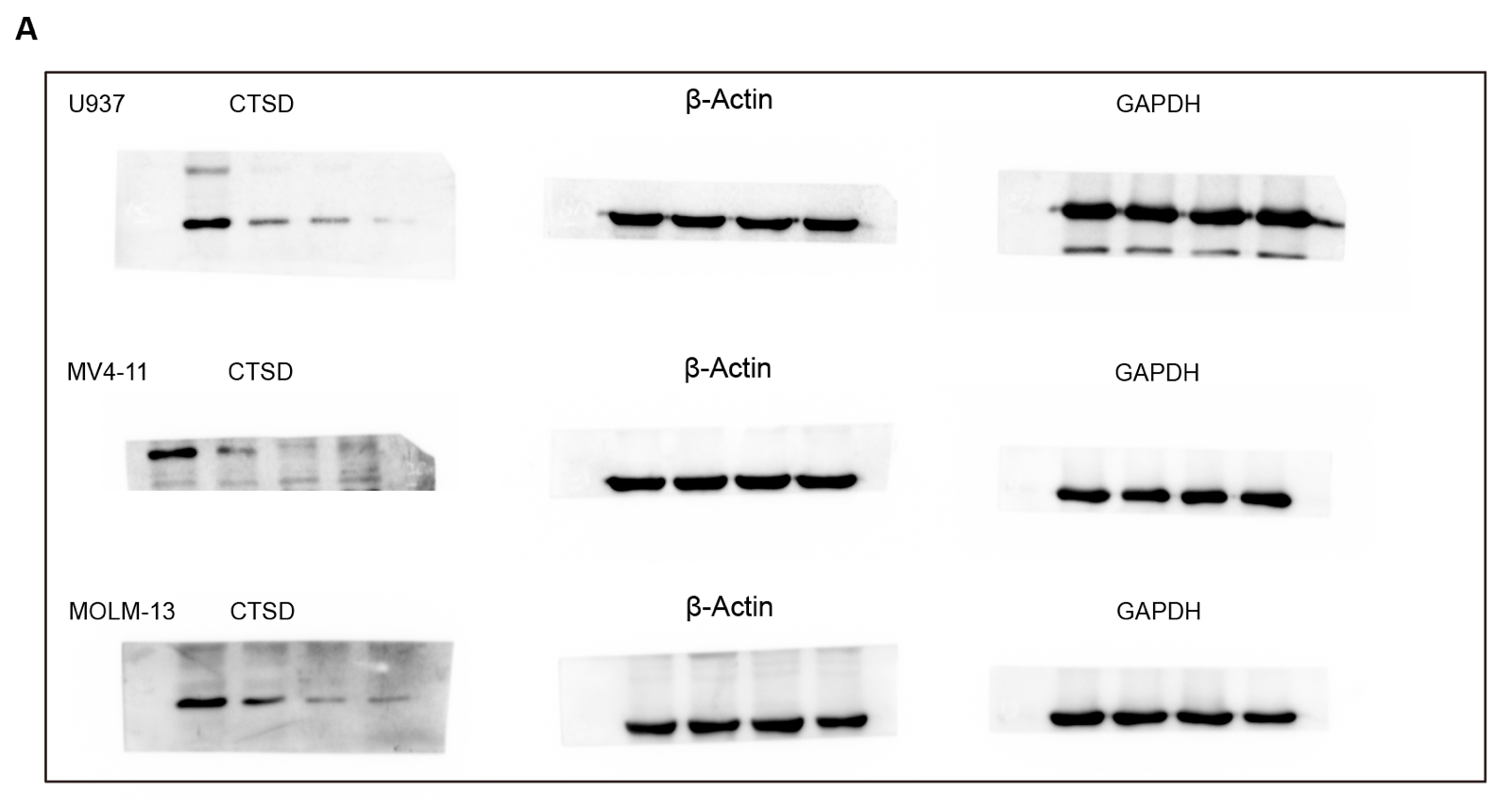


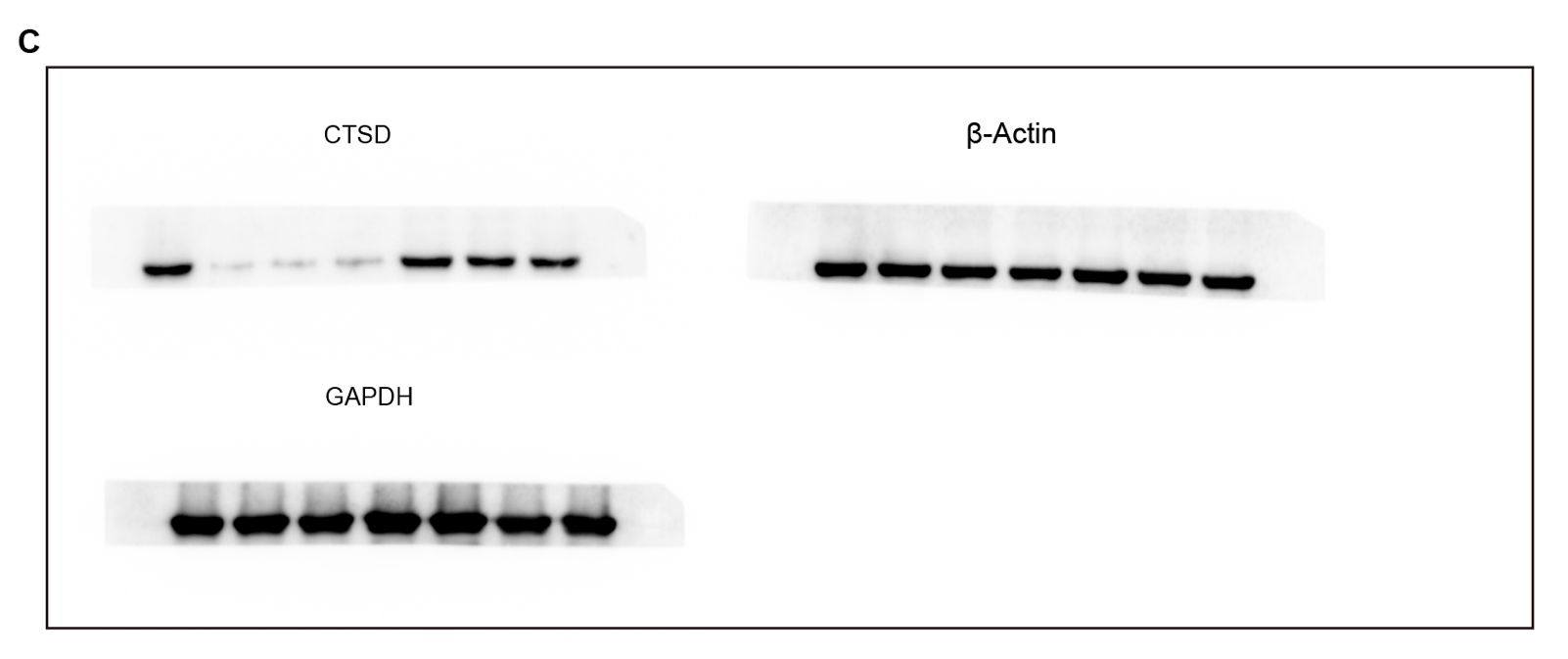


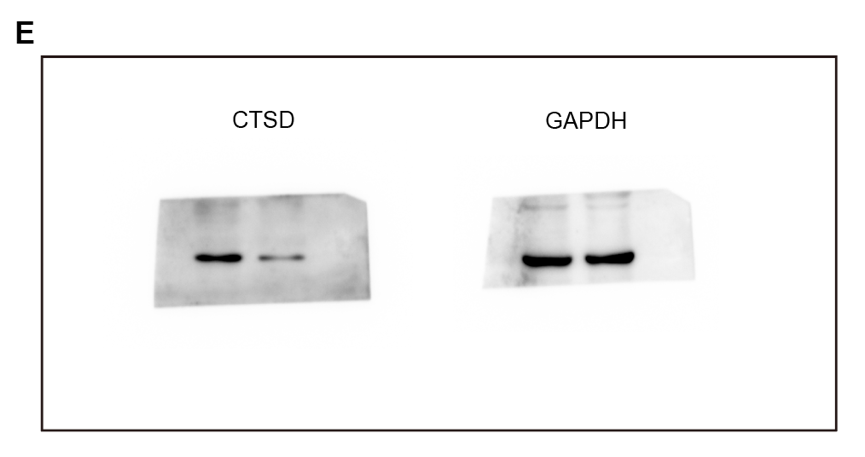


Supplementary Figure 3


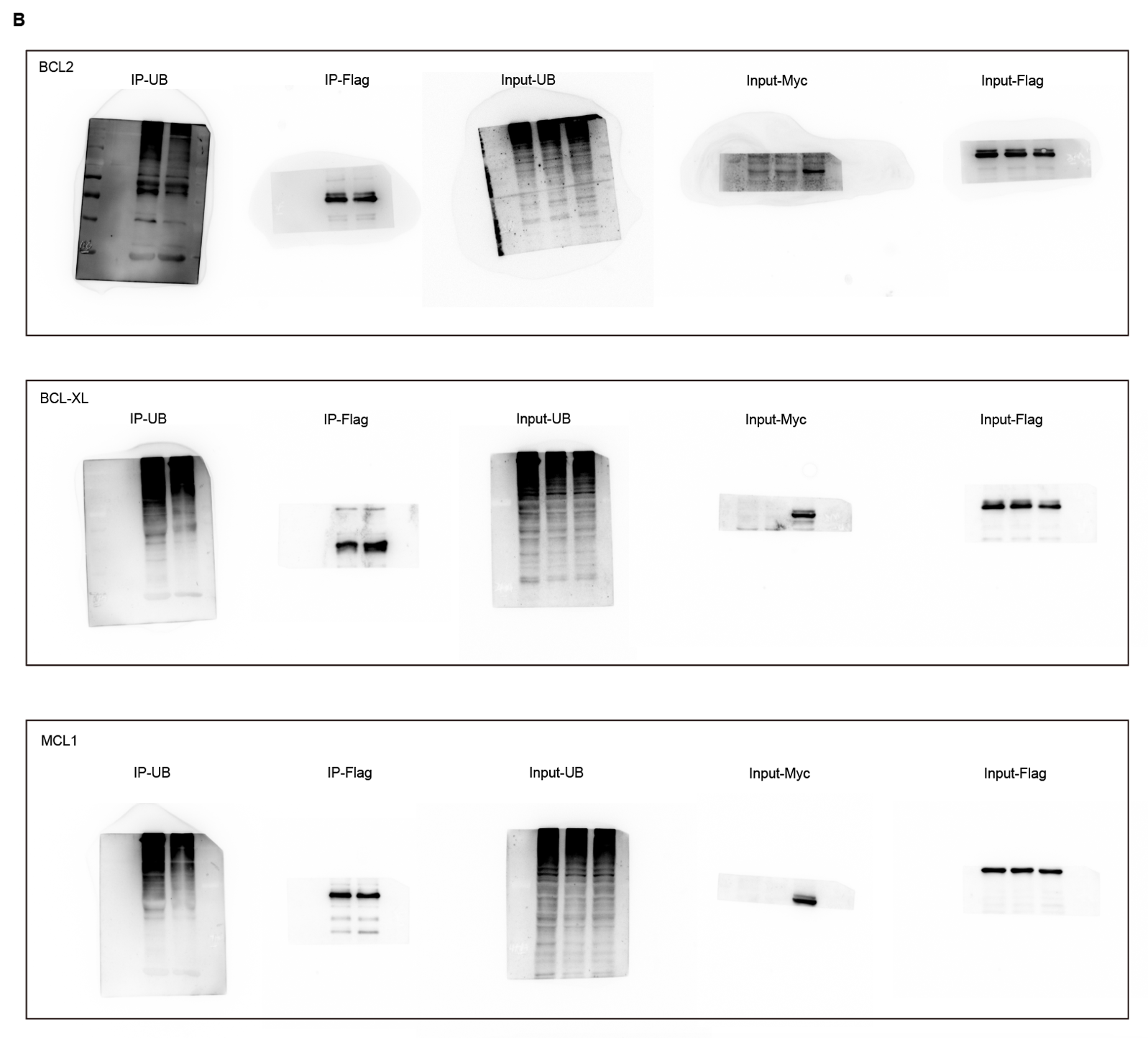


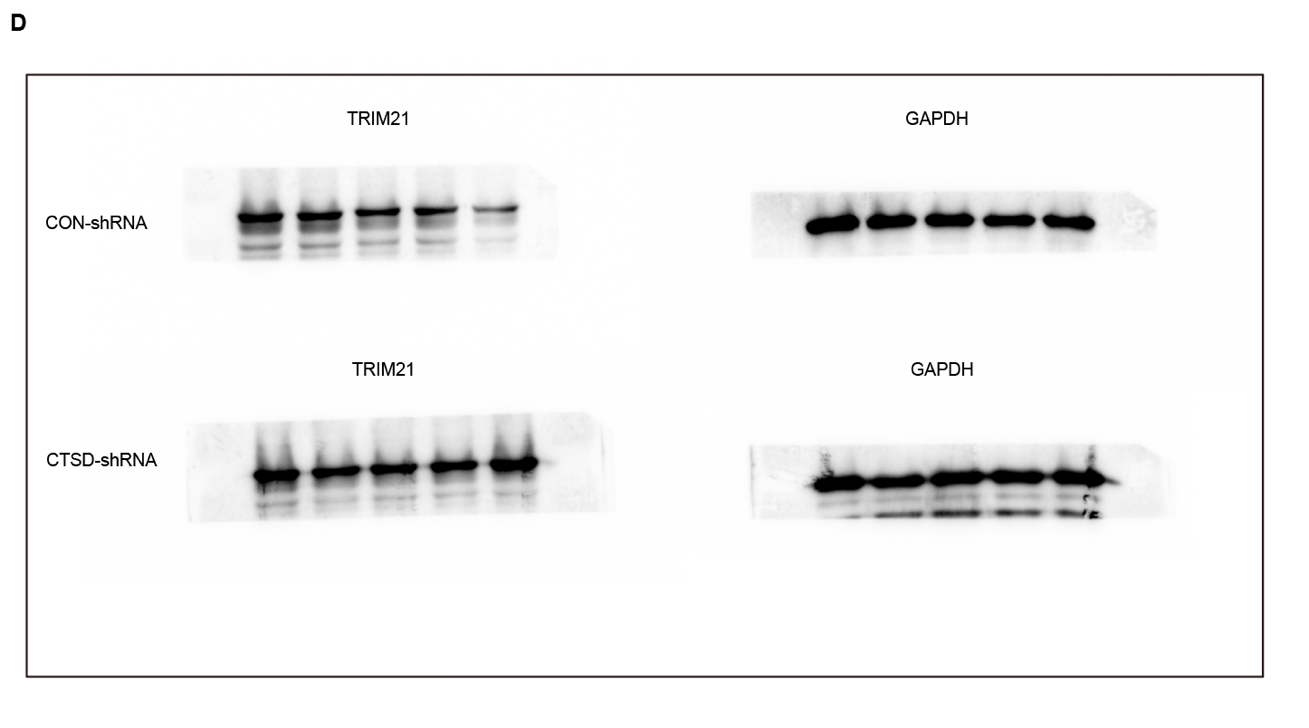


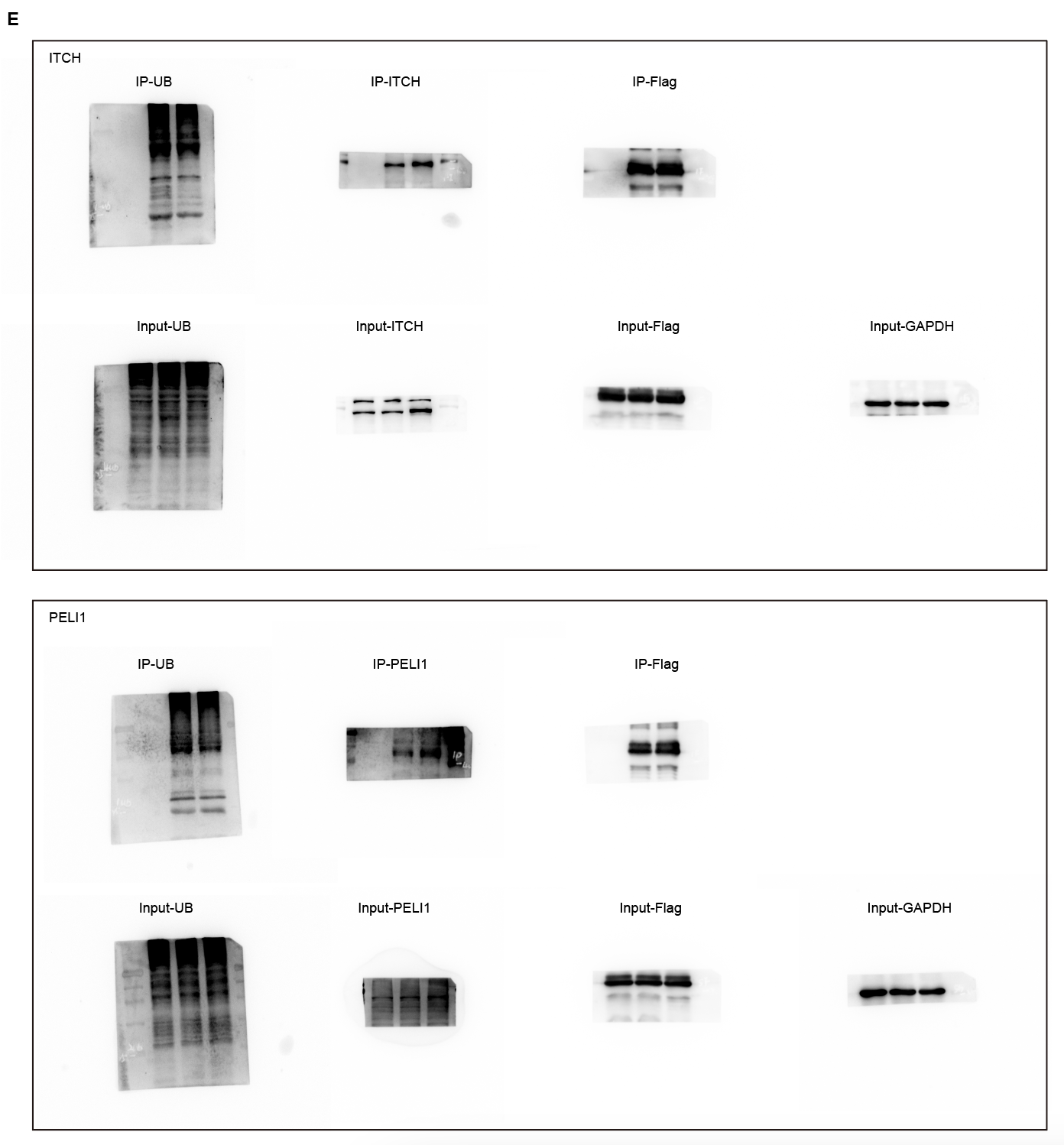


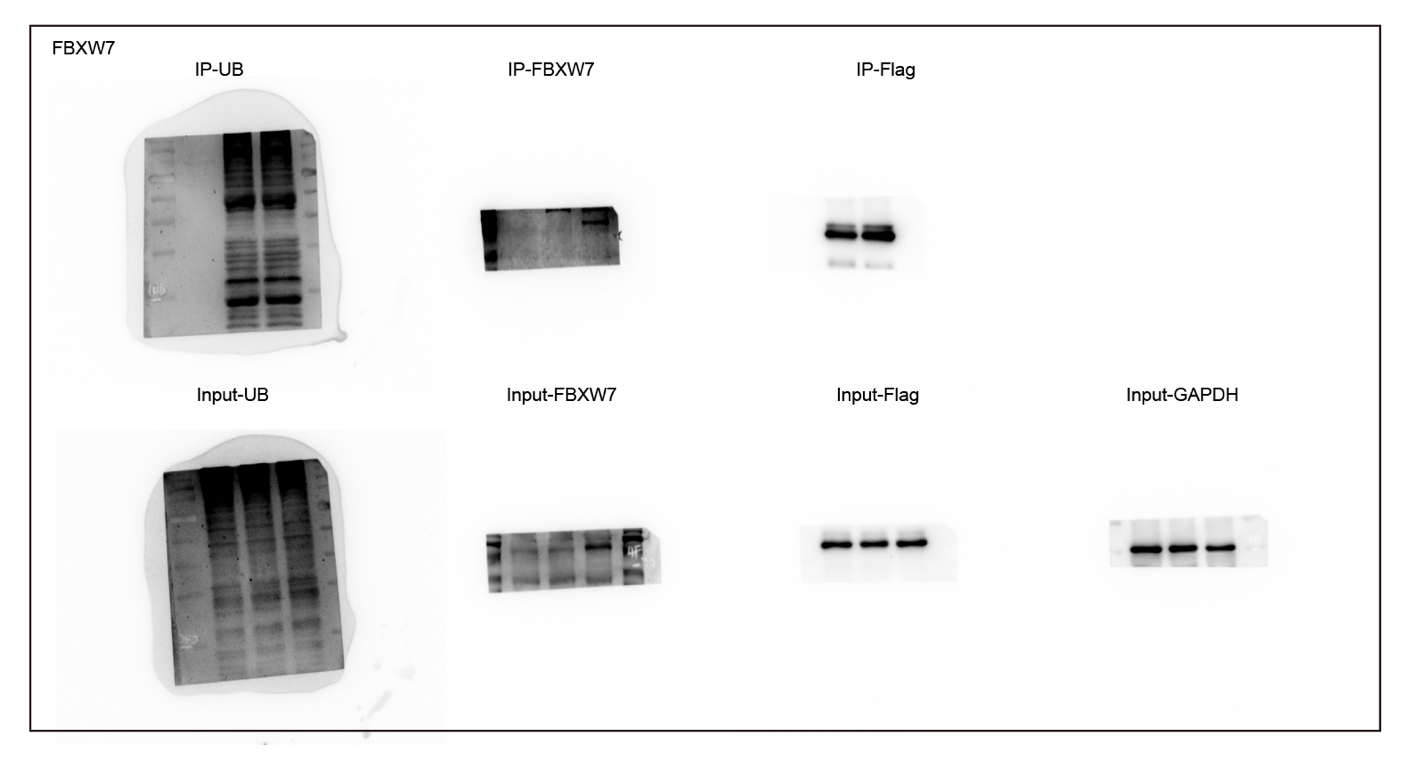


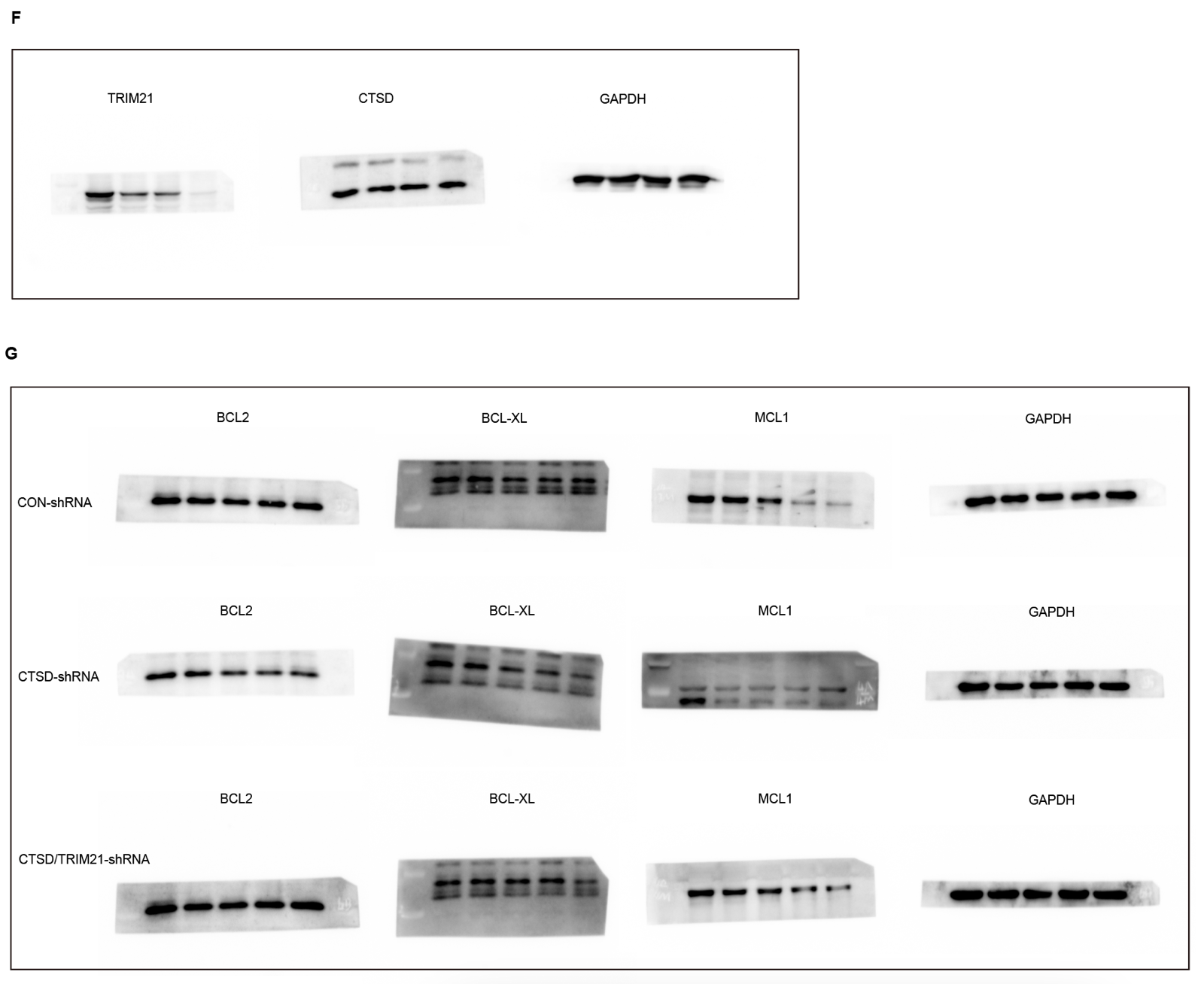


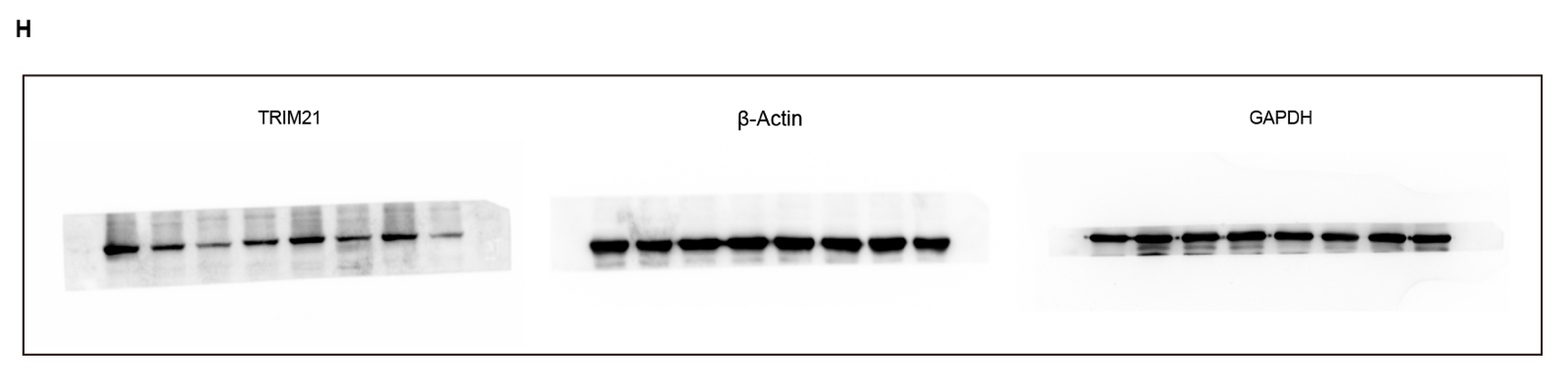


Supplementary Figure 5


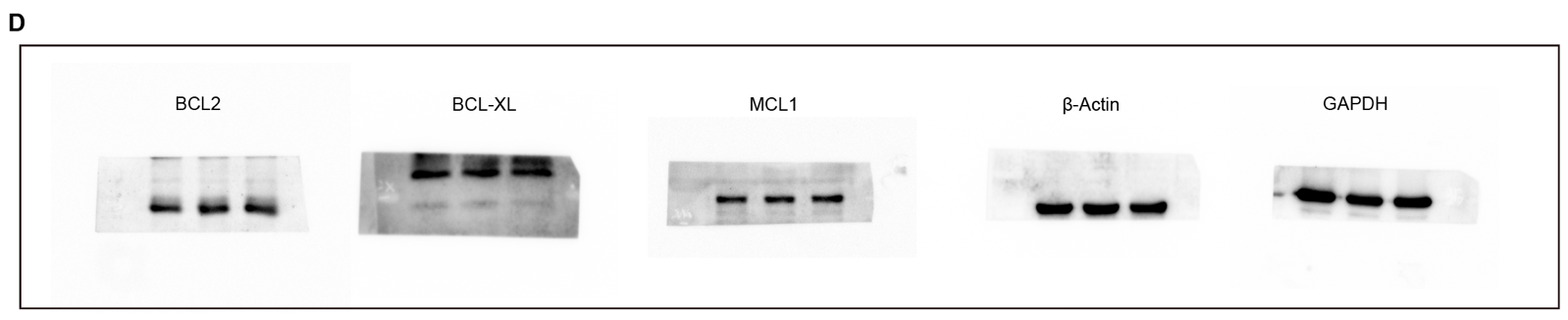

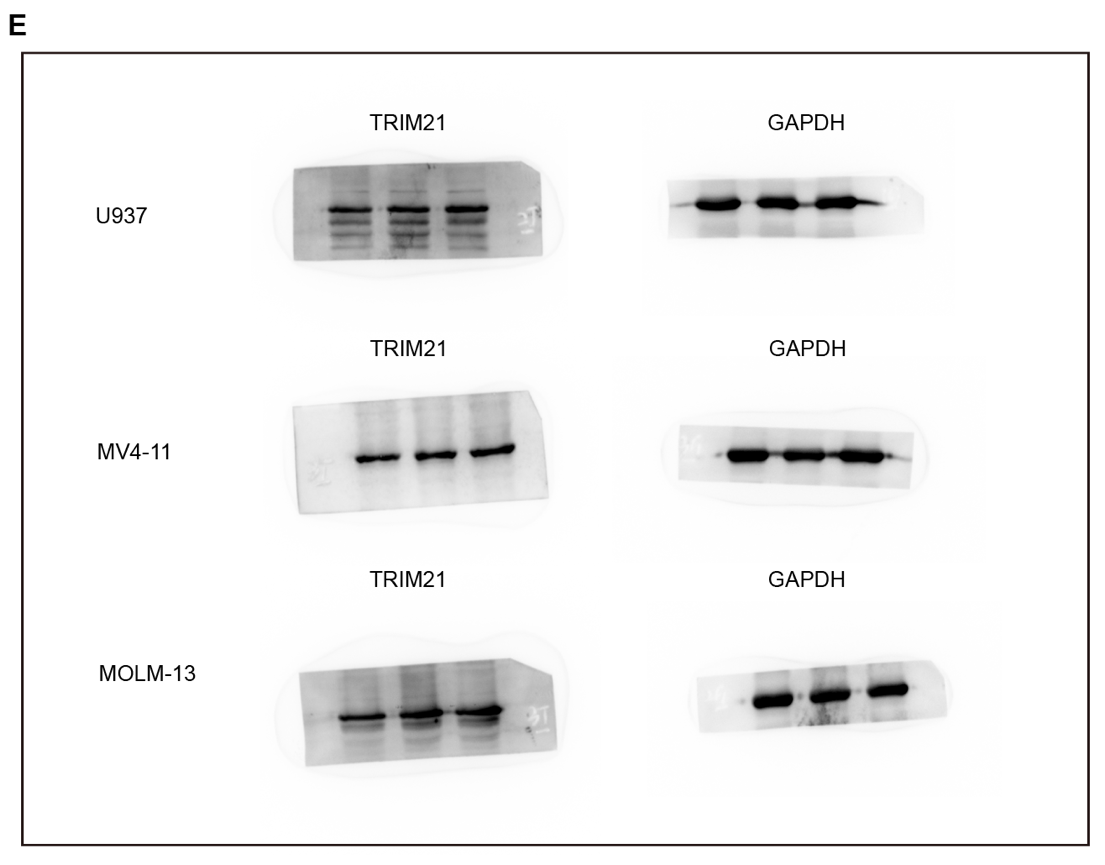


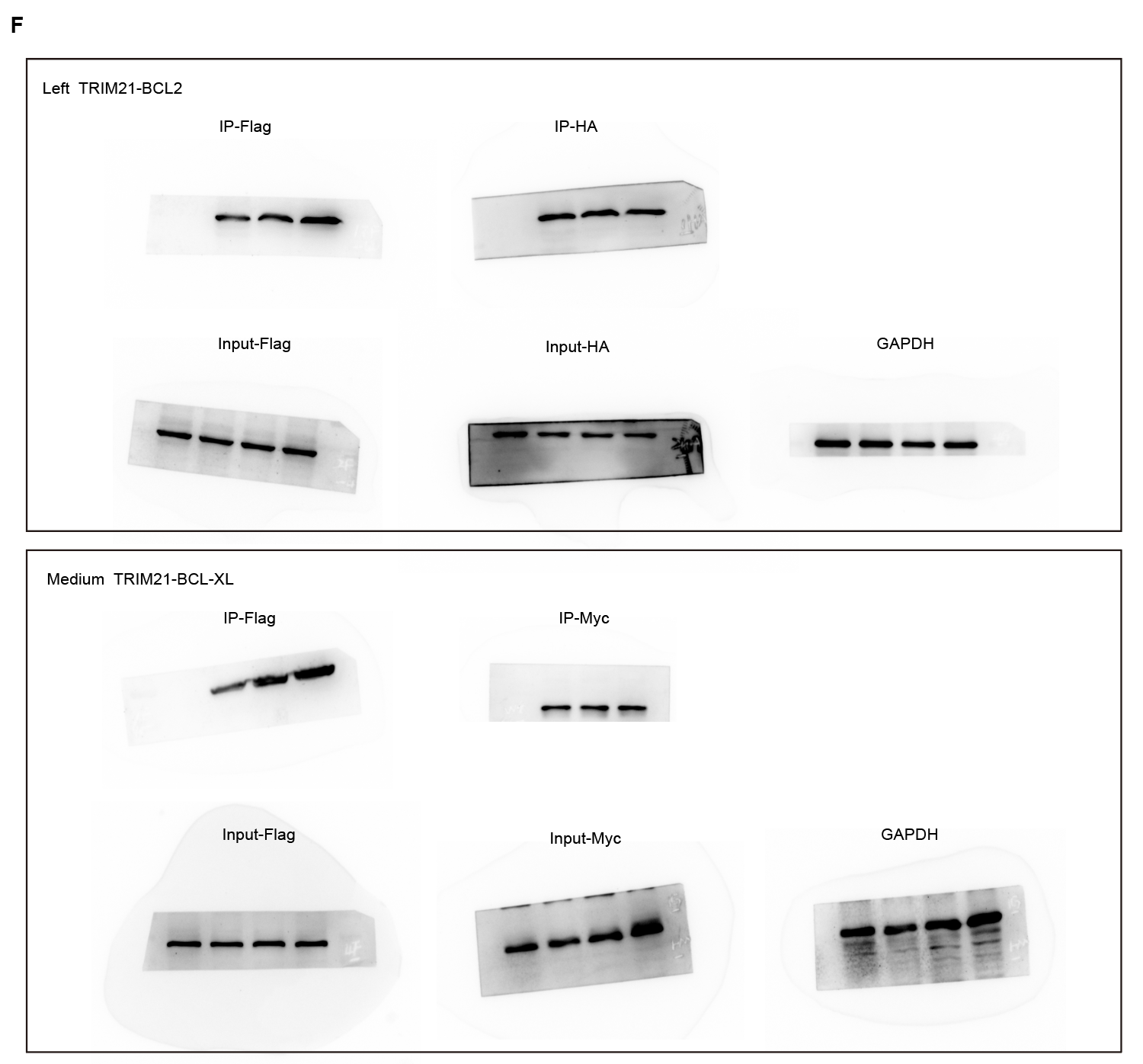


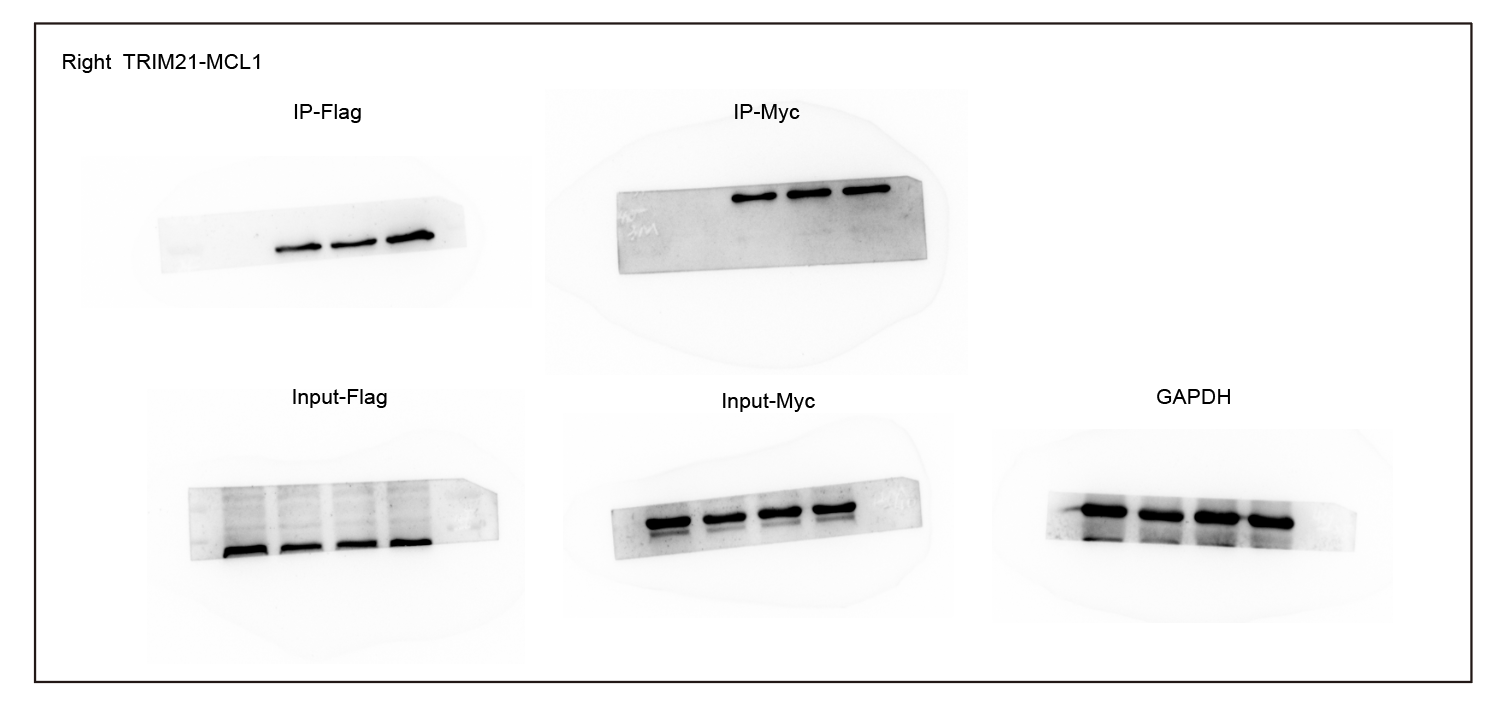

Supplement: Supplementary file 2 — Original Western blots [file 41419_2025_7949_MOESM2_ESM.docx]
